# From helices to superhelices through hierarchical assembly across competing pathways

**DOI:** 10.1038/s41467-026-76656-4

**Published:** 2026-08-11

**Authors:** Kyeong-Im Hong, Jorge S. Valera, Ana M. Garcia, Thomas M. Hermans, Amparo Ruiz-Carretero

**Affiliations:** 1 Instituto de Ciencia de Materiales de Madrid, Consejo Superior de Investigaciones Científicas CSIC, Madrid, Spain; 2 IMDEA Nanociencia, Madrid, Spain; 3 University of Strasbourg, Institute Charles Sadron, CNRS, UPR22, Strasbourg, France; 4 Facultad de Ciencias, Universidad Autónoma de Madrid, Madrid, Spain; 5 Instituto Regional de Investigación Científica Aplicada (IRICA), Universidad de Castilla-La Mancha, Ciudad Real, Spain; 6 Faculty of Chemical Science and Technology, Universidad de Castilla-La Mancha, Ciudad Real, Spain

**Keywords:** Self-assembly, Self-assembly, Optical materials

## Abstract

Understanding how molecular chirality propagates through competing self-assembly pathways remains a central challenge in supramolecular chemistry. We report a chiral π-conjugated quinquethiophene–rhodanine derivative forming four distinct and isolatable supramolecular aggregates from a single enantiomer under identical chemical conditions. These assemblies exhibit different chiroptical signatures, morphologies, hierarchical organization, and are selectively accessed through thermal and mechanical treatment. Calorimetry enables the experimental construction of the energy landscape, while long-term kinetic studies and spectral deconvolution reveal interconversions between helical structures, identifying an off-pathway hierarchical state. Remarkably, the most stable structure forms through a helix-to-superhelix transition governed by secondary nucleation. Cross-seeding experiments demonstrate that different dimensionality assemblies accelerate transformations across hierarchical levels, revealing an unusually interconnected assembly network. These results provide an experimental example where multiple supramolecular structures, their thermodynamic ordering, and kinetic connectivity are established within a single molecular system, showing how pathway selection programs chirality, hierarchy, and morphology in supramolecular materials.

## Abstract (web summary)

Understanding how molecular chirality propagates through competing self-assembly pathways to generate hierarchical structures remains a central challenge in supramolecular chemistry. Here the authors report a chiral π-conjugated quinquethiophene–rhodanine derivative that forms four distinct and isolatable supramolecular aggregates from a single enantiopure monomer under identical chemical conditions.

## Introduction

Helical organization is a fundamental structural motif in Nature, where molecular chirality is amplified across length scales to generate functional architectures such as the α-helix in proteins and the DNA double helix^1,2^. In synthetic supramolecular systems, hierarchical helicity likewise gives rise to emergent properties including circularly polarized luminescence (CPL)^3,4^, chiral charge transport^5^, and chiral-induced spin selectivity (CISS)^4,6,7^. Importantly, recent studies have shown that such functional responses are not determined solely by molecular structure, but also by the supramolecular pathway through which chiral architectures emerge. In particular, pathway complexity and secondary nucleation processes have been demonstrated to influence spin-selective charge transport and chiroptical amplification in supramolecular polymers^8,9^. These observations suggest that controlling hierarchical self-assembly may provide access to multiple functional states from a single molecular scaffold, without requiring chemical redesign. A central objective in supramolecular chemistry is therefore to understand and control how molecular chirality propagates through self-assembly into higher-order structures^10^.

Over the past decade, it has become clear that supramolecular polymerization is inherently pathway-dependent^11^. Competing kinetic and thermodynamic routes can give rise to distinct aggregates from the same molecular building block, and significant progress has been made in identifying the molecular designs and experimental conditions required to control these pathways. While pathway complexity is now widely recognized, experimental access to multiple, well-defined, and stable supramolecular states is still limited. In most systems, alternative assemblies are transient, partially overlapping, or only accessible through chemical modification, photoisomerization, or drastic solvent changes^12–14^. Consequently, supramolecular energy landscapes are often inferred indirectly, and mechanistic relationships between competing aggregates, particularly across different hierarchical levels, remain difficult to resolve.

Chiral supramolecular systems^15,16^ offer unique opportunities to expose hidden assembly pathways^17^, as excitonic coupling and chiroptical signatures provide sensitive structural reporters. Helix-to-superhelix transitions have also been observed, frequently accompanied by inversion of supramolecular chirality in both biological and synthetic contexts^18–20^. However, isolating more than two long-lived chiral supramolecular aggregates from a single enantiopure monomer under identical chemical conditions remains exceptionally rare^8,21,22^. One promising strategy to expand the number of accessible states is to combine pathway complexity with hierarchy in the self-assembly process. In this context, experimental tools that connect assemblies of different dimensionality, such as cross-seeding processes, remain scarce^23–25^, and the controlled use of secondary nucleation to generate hierarchical chiral superstructures is still in its infancy^26–30^.

**Fig. 1:**
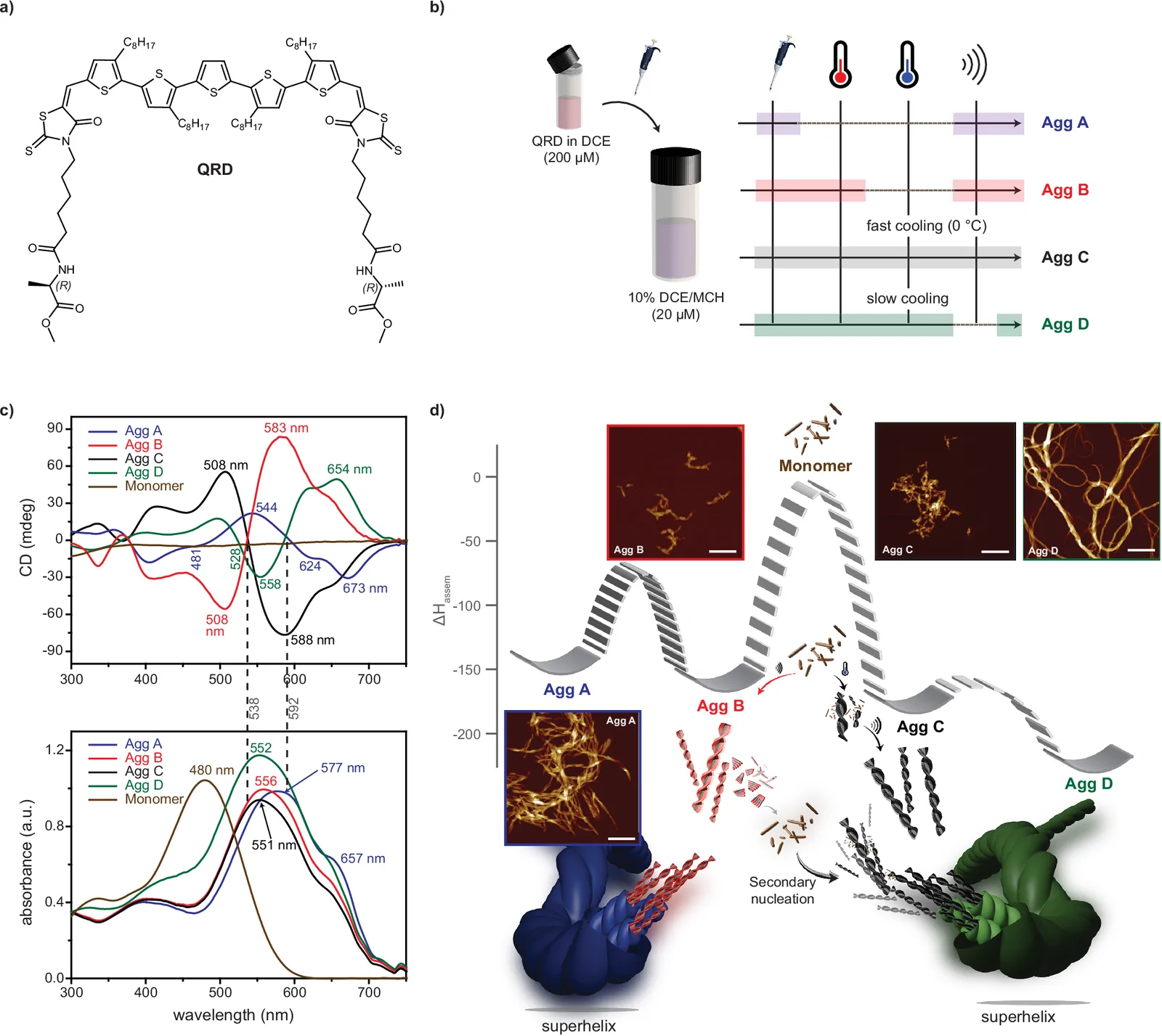
Overview of the QRD system with 4 chiroptical states. **a** Molecular structure of the building-block **QRD**. **b** Preparation protocols of **Agg A**–**D**. **c** Top: CD spectra of **Agg A–D** in solution. [**QRD**] = 20 μM 1:9 DCE/MCH (*l* = 1 cm). Bottom: corresponding UV-Vis spectra at the same conditions. **d** Energy landscape with measured *ΔH*_*assem*_ of each aggregate determined by ITC. AFM images of **Agg A**–**D** on mica substrates (scale bars are 1 μm). The cartoons labeled “secondary nucleation” emphasize the hierarchical assembly pathway leading to **Agg D** (superhelix).

Here we report a chiral π-conjugated quinquethiophene–rhodanine derivative (**QRD**, Fig. 1a) that, through controlled thermal and mechanical protocols, gives access to four distinct and isolatable helical supramolecular aggregates (**Agg A–D**) from a single enantiopure monomer. Each aggregate exhibits a unique combination of chiroptical signature, morphology, and hierarchical organization, yet all are formed in the same solvent environment without chemical modification. The long-lived nature of these states enables direct calorimetric determination of their assembly enthalpies, allowing construction of a quantitative enthalpic energy landscape.

By integrating spectroscopy, concentration-dependent studies, cross-seeding experiments, and thermal/mechanical pathway switching, we experimentally resolve the relationships between these aggregates. We identify kinetically trapped hierarchical states, on-pathway interconversions between one-dimensional helices, and helix-to-superhelix transitions governed by concentration-dependent secondary nucleation. Such hierarchical transitions are particularly attractive because the emergence of superhelical organization can strongly modify excitonic coupling, optical activity, and collective electronic behavior relative to the primary helical state^31^. For example, helix-to-superhelix transformations have recently been associated with enhanced chiroptical responses and tunable circularly polarized emission in π-conjugated supramolecular systems^32^. Remarkably, cross-seeding accelerates transformations between assemblies of different dimensionality, revealing an unusually interconnected energy landscape.

Together, these findings demonstrate that pathway selection alone can program supramolecular chirality and hierarchy, enabling four isolatable chiral structures to emerge from a single molecular building block. Beyond establishing a unique example of mechanistically resolved supramolecular polymorphism, this work highlights how pathway selection can be used to program structurally and functionally distinct chiral states from a single molecular building block. More broadly, these results establish design principles for responsive supramolecular materials in which optical, electronic, or spin-selective properties may be tuned through control over the assembly pathway rather than molecular redesign.

## Results

### Self-assembly assessment – Spectroscopy and microscopy

The self-assembly behavior of **QRD**^33,34^ (see Fig. S1 and Section S2 of the Supplementary Information for all the synthesis and characterization details) was explored using UV-Vis and circular dichroism (CD) spectroscopy in a mixed solvent system composed of a good solvent (1,2-dichloroethane, DCE) and a poor solvent (methylcyclohexane, MCH). Practically, a 200 μM **QRD** stock solution in DCE is diluted with MCH to obtain a 20 μM (unless otherwise specified) solution (in a 1:9 DCE/MCH ratio). Four preparation protocols were used (Fig. 1b), resulting in aggregates **A**–**D** (in order of decreasing elongation enthalpy *ΔH*_*assem*_, as explained below). The first type of aggregate (**Agg A**) is obtained by room temperature sonication of the resulting 20 µM solution for 30 min. When using a traditional heat/cool protocol—i.e., 20 μM **QRD** in 1:9 DCE/MCH solution was heated to 70 °C to fully depolymerize the sample, followed by controlled cooling at 1 K min^–1^ to 15 °C—**Agg D** is formed. Increasingly complex combinations of temperature and sonication are used in the field to facilitate alternative nucleation pathways, disrupt metastable intermediates, and minimize fiber bundling^35–37^. In our hands, this resulted in two additional states, namely, **Agg B**: hot (70 °C) 20 µM **QRD** in 1:9 DCE/MCH solution is placed in a room temperature sonication bath for 30 min, or **Agg C**: hot (70 °C) 20 µM **QRD** in 1:9 DCE/MCH solution is cooled in a 0 °C ice bath for 30 min and then sonicated at room temperature for 30 min. The detailed preparation protocols of **Agg A**–**D** can be found in Section 3 of the Supplementary Information.

All aggregates show a bathochromic (red-)shift of their UV-Vis absorption maxima with respect to the monomer (cf. Fig. 1c, bottom), as well as the appearance of a new J-band ~657 nm, both indicative of J-type aggregation^38,39^. In addition, we confirmed the formation of hydrogen bonds using infrared spectroscopy (Fig. S2). Whereas the N–H stretching band, Amide I and II of **QRD** measured in a good solvent (CDCl_3_) exhibit values of 3435, 1674 and 1564 cm^−1^, respectively, these bands experience a shift toward lower wavenumbers for aggregates **Agg A**–**D** (with values ranging from 3309 to 3307 cm^−1^ for the N–H band, 1647–1645 cm^−1^ for the Amide I band and 1575–1578 cm^−1^ for the Amide II band) proving the formation of intermolecular hydrogen bonding for the four aggregates.

Intriguingly, we can discern 4 distinct CD spectra (Fig. 1c, top). None of **Agg A**–**D** shows a zero crossing in CD that perfectly coincides with the aggregate absorption maximum, which shows that the assembly is more complex than the simple J-aggregation model^30,31^. Instead, **Agg B** and **Agg C** show a blue-shifted zero crossing in CD versus their absorption maximum (–18 and –13 nm, respectively), which shows that additional low-energy states (from extended excitonic delocalization) are present in absorption but not strongly contributing to the excitonic CD signature. In contrast, **Agg A** and **D** show a complex CD signal with multiple zero crossings, with the most intense band crossing (for both) at 592 nm, which is red-shifted by +15 and +40 nm as compared to their absorption maxima. Some nearest-neighbor exciton contributions remain with crossings at 481 nm and 528 nm, respectively. Overall, long-range excitonic delocalization^40^, and therefore the degree of bundling and possible formation of superhelices (see below), for **Agg A** and **D** is greater than that of **Agg B** and **C**.

This is further supported by morphology analysis by dry-state AFM (Fig. 1d, images) as well as solution-state superresolution confocal microscopy (Fig. S3). Indeed, **Agg B** and **C** show aggregates with lengths of 642 ± 278 nm and heights of 29 ± 7 nm, and lengths of 337 ± 201 nm and heights of 22 ± 4.5 nm, respectively. In contrast**, Agg A** and **D** show much larger superhelical bundles of several µm lengths and heights of 42 ± 14 nm and 54 ± 29 nm, respectively (Figs. S4–S7). The helical pitch of **Agg B** has an average size of 14.64 ± 1.31 nm, whereas **Agg C** shows an average helical pitch of 14.99 ± 1.10 nm (Fig. S8). The helical pitches of **Agg A** and **Agg D** were more irregular, with average helical pitches of 203.75 ± 23.52 nm for **Agg A** and of 157.47 ± 34.30 nm for **Agg D**, respectively (Fig. S9). The left-handedness observed for **Agg A** and **C** agrees with the negative bisignate Cotton effect (Fig. S10 and Supplementary Video S1). Similarly, right-handed structures are found for **Agg B** and **D**, consistent with the positive bisignate CD signal (Supplementary Video S2 and Fig. S10)^41^. The sum of the spectroscopic and microscopic characterization unravels that the system is composed of two helical aggregates of smaller sizes (**Agg B** and **Agg C**) and two superhelical aggregates (**Agg A** and **Agg D**) with opposite helicities between each pair of aggregates. **Agg B** and **C** have a higher local order as evidenced by a sharp peak with d-spacing 2.8 Å (Fig. S11), whereas **Agg A** and **D** have broad, undefined Bragg reflections pointing to a more amorphous packing.

### Determination of the energy landscape

Having established the morphology and structural basis of **Agg A**–**D**, we sought to understand the energy landscape from the monomer to each aggregate. Such landscapes are often only qualitative^42^ or based on simplified (one-dimensional polymerization) models^43,44^. In our case, given the presence of bundles and/or superhelices, 1D models would be a clear over-simplification. Therefore, we used isothermal titration calorimetry (ITC) to determine the enthalpies of assembly (see Section 5 in Supplementary Information for all the ITC sample preparation details and control experiments, Figs. S12–S19 and Tables S1 and S2)^45–47^. Specifically, we injected aliquots of 5–10 µM **Agg A**–**D** solutions (separately) into the parent solvent (1:9 DCE/MCH mixture) at 25 °C, leading to dilution by a factor of 70 and thus, full disassembly inside the calorimeter (as verified by CD spectroscopy and plotting the molar ellipticity for each of the aggregates, Fig. S15). Disassembly is an endothermic process resulting from the breaking of stacking, as well as bundling interactions, visible as positive heat peaks in the thermograms (Table S2 and Figs. S16–S19). We integrate the dilution peak (kJ) and divide by the number of moles (mol) of that specific injection, resulting directly in the enthalpy of disassembly (kJ/mol) without further assumptions. To allow comparison to other systems, we present the enthalpy of assembly Δ*H*_*assem*_ = –Δ*H*_*diss,calor*_ in Fig. 1d.

Our analysis shows that **Agg A** (Δ*H*_*assem,****A***_ = –139.9 ± 3.2 kJ/mol) is the least stable. It is followed by **Agg B** (Δ*H*_*assem,****B***_ = –159.1 ± 1.7 kJ/mol), **Agg C** (Δ*H*_*assem,****C***_ = –176.7 ± 3.5 kJ/mol), and finally **Agg D** is the most stable (Δ*H*_*assem,****D***_ = –221.2 ± 13.3 kJ/mol), likely due to its formation via slow cooling. Although **Agg D** displays the most favorable assembly enthalpy, the pronounced thermal hysteresis and the time-dependent evolution of the CD signal after cooling indicate that its formation proceeds through kinetically controlled, nucleation-limited pathways rather than immediate thermodynamic equilibration (Fig. S20)^48,49^. Thus, these measurements indicate that **Agg D** is much more stable than **Agg A**, whereas **Agg C** is slightly more stable than **Agg B**, with differences in energy that disclose potential transitions between these aggregates (Fig. 1d, cartoons), as well as the presence of competing pathways.

### Spontaneous conversions between Agg A and Agg D

**Fig. 2:**
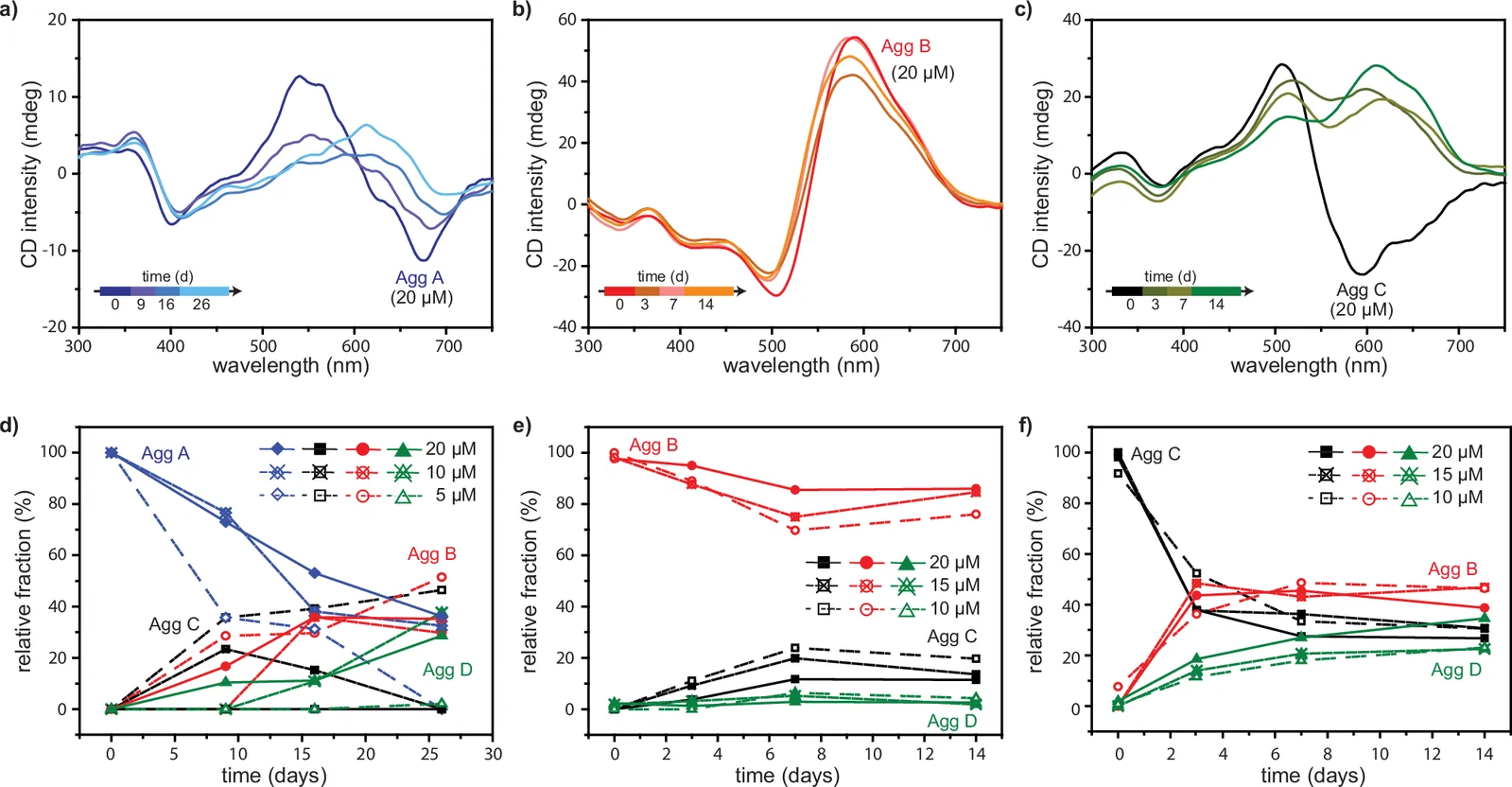
Off/On-pathway spontaneous conversions. CD spectral changes of **a** **Agg A** (20 μM, 1/9 DCE/MCH) over the course of 26 days at room temperature, **b** **Agg B** (20 μM, 1/9 DCE/MCH), and **c** **Agg C** (20 μM, 1/9 DCE/MCH) over the course of 14 days at room temperature. The relative aggregate fractions obtained by spectral deconvolution (see Section 6 in Supplementary Information) at different concentrations for **d** **Agg A**, **e** **Agg B**, and **f** **Agg C**.

Next, we aim to understand spontaneous transitions toward lower energy states, as one would expect on the path toward more thermodynamically favored (i.e., lower Δ*H*_*assem*_) states (Fig. 2). All transitions were followed by CD spectroscopy, where we used the individual spectra of Fig. 1c to perform spectral deconvolution (see Section 6 in Supplementary Information). This allows us to quantify the % fraction of each aggregate (**Agg A**–**D**) at any point in time. A priori, we expected **Agg A** to evolve to **Agg B,** **Agg C**, or **Agg D**. To investigate this, we performed time-dependent experiments on **Agg A** at different concentrations (5, 10, and 20 μM) in 1:9 DCE/MCH at room temperature (Fig. 2a, d and Table S3). After preparation of **Agg A** under standard sonication conditions, CD spectra were recorded over 26 days. The time-resolved CD spectra were subsequently deconvoluted using linear combinations of the reference spectra of **Agg A**–**D** (Section 6.1 in Supplementary Information), allowing us to quantify the relative fraction of each aggregate as a function of time. The results show clear concentration dependence: at 5 μM, **Agg A** decreases relatively rapidly. The spectral deconvolution reveals that **Agg A** mainly converts into **Agg B** and **Agg C**. However, no significant direct formation of **Agg D** is observed at early stages. The conversion from **Agg B/C** to **Agg D** remains slow under these dilute conditions. At 10 μM, a similar trend is observed, although the conversion is slower than at 5 μM. Finally, at 20 μM, **Agg A** persists longer, and over time, a measurable fraction of **Agg D** appears. At this higher concentration, the conversion from intermediate **Agg B/C** toward **Agg D** becomes more significant.

Importantly, in none of the experiments did we observe a direct, rapid conversion of **Agg A** into **Agg D**. Instead, **Agg A** first evolves into **Agg B** and/or **Agg C**, and only subsequently, under conditions where the **Agg B/C → Agg D** pathway is operative, does **Agg D** emerge. This behavior implies that **Agg A** is not on the direct pathway toward **Agg D** but rather requires partial depolymerization and reorganization before accessing the **Agg B/C → Agg D** *on-pathway* landscape. As a reminder, *“on-pathway”* is a stepping stone toward the intended state, whereas *“off-pathway”* denotes a detour that traps monomers in a non-productive state. **Agg A** is thus *off-pathway* toward **Agg D**^12,13^.

The situation changes for **Agg B**, which slowly converts by ~20% to yield mostly **Agg C**, and a trace amount of **Agg D**, over the course of 14 days, this transition being slightly more pronounced at lower concentrations (Fig. 2b, e and Section 6.2 in Supplementary Information). When starting from **Agg C,** **Agg D** is formed to a greater extent (~ 25%), and interestingly, **Agg B** is also formed (~40%, Fig. 2c,f). These two findings show that **Agg B** and **C** can interconvert (which makes sense given the fact that they have relatively close Δ*H*_*assem*_) and are *on-pathway* toward **Agg D** (Tables S4 and S5 and Figs. S21 and 22). No changes are detected when starting from **Agg D**, even after 1 month.

So far, these spontaneous conversions suggest that **Agg B** is *off-pathway* to **Agg C** (this transition occurs via the monomer, Fig. 1d, cartoons), given the slow transition between these aggregates and the fact that this conversion is slightly enhanced at lower concentrations. Furthermore, it seems that high concentrations of **Agg C** favor the transition of this supramolecular entity to **Agg D**, which agrees with a hierarchical process, i.e., a consecutive pathway from the helical structure toward a species of higher dimensions (superhelix). From the evolution and variable concentration experiment results shown by **Agg A**, it can also be envisioned that this aggregate constitutes a hierarchically organized species highly connected to **Agg B**.

### Seeding experiments, secondary nucleation, and thermal and mechanical treatments

The use of seeds has been reported to accelerate the transformation between aggregates of different stabilities at the same level of organization^23,42,48–50^, and seldom between aggregates of different dimensionalities^23–25^. In our system, given the effect that sonication exerts in the preparation of the different aggregates, the formation of seeds through ultrasonication, as usually reported in the literature, was not feasible. Thus, here we refer to “seeding” as the addition of aliquots of **Agg B**–**D** as prepared per the protocols explained above (see Section 7 of the Supplementary Information for details). The lengths of **Agg B** and **Agg C** (around 300 nm and 600 nm, respectively) are higher than most of the seeds employed in the literature (30–100 nm). However, notable exceptions with longer lengths have also been utilized (>800 nm)^51,52^, which have actually helped trigger other assembly phenomena, such as secondary nucleation.

Thus, we first attempted to accelerate the conversion of **Agg C** into **Agg D** with different (**Agg D**) cross-seed amounts, but no rate change was observed (Fig. S23). At this moment, we suspected that in **Agg D** formation, there was a large contribution of secondary nucleation due to the protocol applied (slow cooling rates and plausible supersaturation conditions). To assess the contribution of secondary nucleation in this aggregate, we followed the formation of **Agg D** from the monomer after applying a quick cooling rate (10 K min^−1^), and fitted the resulting kinetics to the distinct models of the Amylofit software^53^. The slope, corresponding to the scaling factor γ, obtained after representing log t50 versus log monomer was of *γ *= −2.777, showing lower mean residual error for the secondary nucleation model compared to fragmentation and nucleation-elongation models (Figs. S24–S26), which further reinforces this interpretation. The Amylofit software evaluates the transition of the monomer to the aggregate state (with or without seeds), and therefore, the available models are not suitable to analyze the rest of the aggregates. Furthermore, due to the unique combination of sonication, temperature, and cooling rates required to prepare most of the aggregates, we could not obtain real-time spectroscopy of transitions of **Agg A**–**Agg C**, making kinetic analysis impossible.

**Fig. 3:**
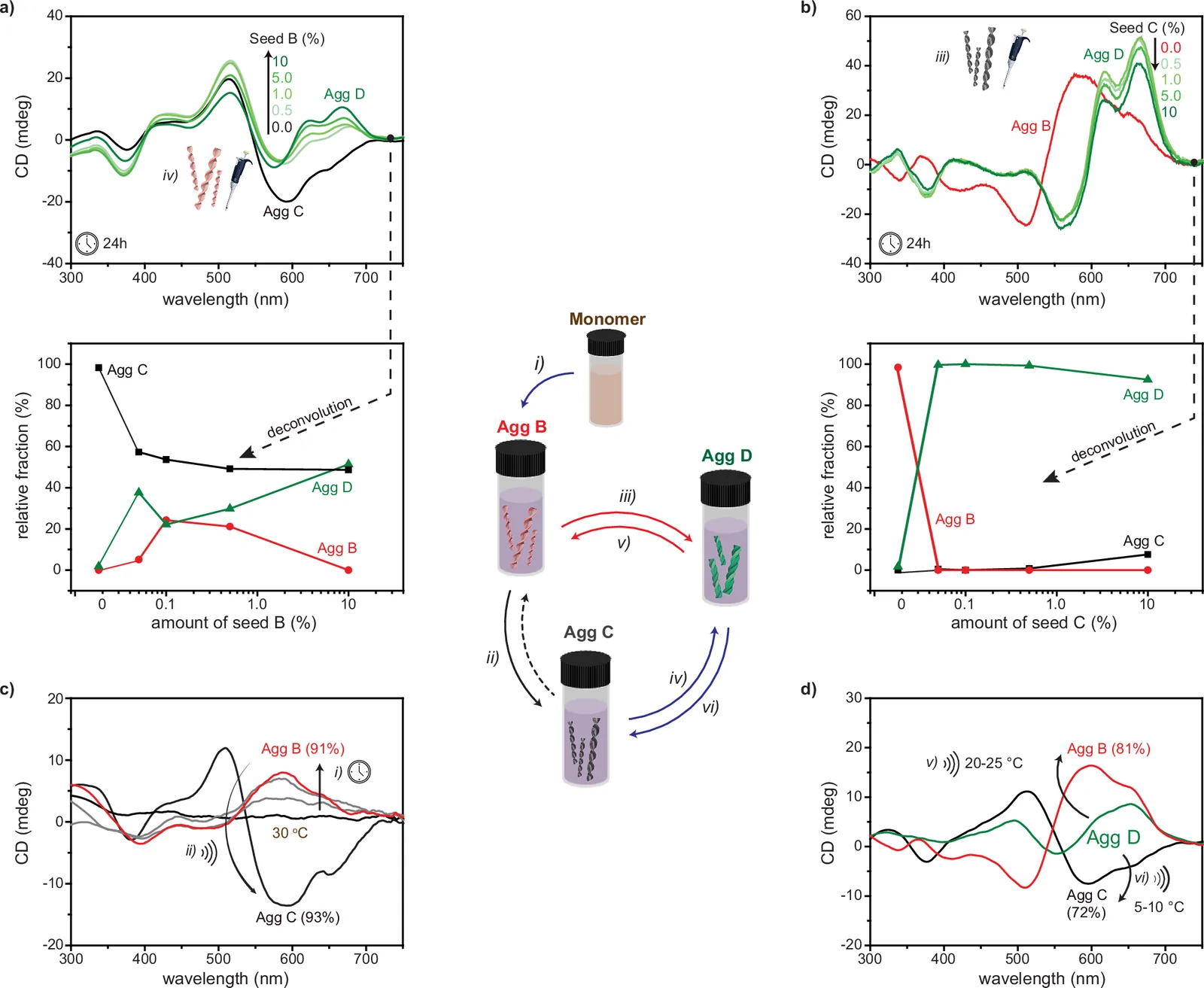
Interconversions of Agg B–D. **a** **Agg C** (10 μM, 1:9 DCE/MCH) evolves to **Agg D** by the addition of **Agg B** seeds, and **b** **Agg B** (10 μM, 1:9 DCE/MCH) is converted to **Agg D** in the presence of **Agg C** seeds. **c** **Agg B** (10 μM, 1:9 DCE/MCH) formed at 30 °C is transformed to **Agg C** by applying sonication. d **Agg D** (10 μM, 1:9 DCE/MCH) are converted into **Agg B** and **Agg C** by sonication at different temperatures.

Surprisingly, the cross-seeding of **Agg B** (0.5–10%) into **Agg C** results in up to 50% **Agg D** being formed in 24 h (Tables S4 and S5, Fig. S27)—as compared to ~30% after 14 days without seeding (cf. Fig. 2c, f). The latter corresponds to a rate enhancement of ~27 times (assuming pseudo-first order conversion as we are in elongation-dominated conditions). The cross-seeded acceleration is even more shocking when seeds of **Agg C** are added to **Agg B**, where >99% conversion to **Agg D** was found after 24 h (Fig. 3b, seeds <1%). Compared to the unseeded (Fig. 2a, d) case, the rate enhancement is a factor of ~2000. Thus, it can be inferred from these experiments that, whereas **Agg D** is not able to surface catalyze its own formation, **Agg C** is more efficient in this task. This interpretation is in agreement with certain amyloid systems^54^, where fibril thickness and hierarchically organized species have been shown to decrease secondary nucleation^55–57^, or it can be a cause of the size and shape of the seed itself^58^.

Taken together, the (un-)seeded experiments suggest the following: (i) **Agg D** is a superhelix of **Agg C** (Fig. 1d, cartoons), since the latter is accompanied by chirality inversion^19,27^ and we do indeed observe a flipping of the CD signal (compare green and black spectra in Fig. 1c, top); In a similar argument, **Agg A** should be a superhelix of **Agg B**, but cannot be formed since it is energetically uphill (cf. Figure 1d); (ii) therefore, **Agg B**—unable to convert to **Agg D**—acts as monomer reservoir accelerating **Agg C** conversion into superhelix **Agg D** (Fig. 1d, cartoons and Fig. 2c,d); (iii) this explains why the conversion from **Agg B** to **Agg D** is very fast in presence of seeds of **Agg C** (Fig. 3b), whereas the opposite experiment (**Agg C** to **Agg D** with seeds of **Agg B**, see Fig. 3a) is much slower (by a factor ~2000, see above)^59^.

The inversion of chirality observed during the transition from helices to superhelices may appear counterintuitive at first. However, such behavior is in fact common in hierarchical chiral assemblies. In many supramolecular polymers that evolve from primary helices into superhelical bundles or higher-order chiral structures, an inversion of handedness between the two hierarchical levels has been reported^18–20^. This phenomenon has generally been rationalized as a consequence of the relaxation of torsional stress present in the primary supramolecular helices, which can be relieved when the fibers coil around each other^18^. Nevertheless, the origin of the resulting chirality inversion has rarely been fully clarified in artificial supramolecular systems.

In contrast, analogous behavior has been extensively studied in the context of DNA supercoiling^60^. In such systems, twisting of a primary helix generates supercoils that often adopt the opposite handedness because this configuration more efficiently relaxes torsional strain. Although supercoiling can in principle occur in either direction, opposite-handed coiling typically provides more effective stress relaxation and is therefore energetically favored. Nevertheless, the transition from helices of **Agg B** (Δ*H*_*assem,****B***_ = –159.1 ± 1.7 kJ/mol) to superhelices of **Agg A** (Δ*H*_*assem,****A***_ = –139.9 ± 3.2 kJ/mol) is associated with an enthalpic penalty. We rationalize this result considering the molecular chirality of the monomer **QRD**. The helicity of **Agg D** is enthalpically favorable considering the chiral center in monomer **QRD**. We experimentally validated it by checking the mirror-imaged derivative **QRL** (Fig. S28). For the latter, all the helicities of the aggregates are opposite to those shown in Fig. 1c. Therefore, a match between molecular chirality and superhelicity of **Agg D** is the most plausible explanation. Considering the distinct level of organization in the self-assembly of **Agg C** and **Agg D** and the seeding experiments results, it is reasonable to propose that **Agg D** is a superhelical structure formed through secondary nucleation of **Agg C** (Fig. 1d, cartoons). The conditions that produce **Agg D** (i.e., supersaturation combined with slow cooling) are consistent with hierarchical growth processes that favor secondary nucleation and subsequent fiber bundling. Similarly, the formation of **Agg A** under rapid solvophobic quenching conditions suggests that this superstructure may originate from secondary nucleation processes involving **Agg B** and **Agg C**. Further support for this hypothesis comes from concentration-dependent experiments: when the preparation protocol used for **Agg D** is applied at lower concentrations (3.5 μM), **Agg C** is initially formed and subsequently evolves partially into **Agg D**. Together, these observations support a hierarchical pathway in which helical aggregates (**Agg B/C**) act as precursors for the formation of superhelical assemblies (**Agg A/D**).

More broadly, the preparation protocols themselves act as pathway-selection variables. Cooling rate, thermal treatment, and mechanical perturbation modify local supersaturation and nucleation kinetics, thereby leading to competing assembly routes. Under slow cooling, gradual equilibration favors hierarchical growth and secondary nucleation processes that ultimately yield **Agg D**. In contrast, rapid sonication-assisted protocols combined with thermal treatment (**Agg A**–**C**) likely generate alternative nucleation environments and debundling that stabilize **Agg B**–**C** before hierarchical reorganization occurs, emphasizing the crucial role of the preparation protocols in achieving complex but controllable systems.

Lastly, we combined thermal and sonication procedures to access aggregate interconversions that do not occur spontaneously, nor via cross-seeding. Cooling a **QRD** 10 μM 1:9 DCE/MCH solution from 70 to 30 °C at a 1 K min^−1^ cooling rate led to a null CD signal that in 1 h evolved to form **Agg B** (Fig. 3c). Sonication at 10 °C for 30 min provoked the conversion of **Agg B** to **Agg C**. This transition agrees with **Agg B** being off-pathway to **Agg C**. Considering the largest and most hierarchical of all **Agg D**, sonication at controlled temperatures yields either **Agg C** (5–10 °C) or **Agg B** (20–25 °C, see Fig. 3d). This demonstrates that **Agg B** and **Agg C** are close in energy—as we previously showed (Fig. 1d), with a kinetic barrier which still requires the use of ultrasonication. Also, this “debundling” from **Agg D** to **Agg B** must proceed via **Agg C** and subsequent monomer formation, which would explain the higher temperatures required to fully depolymerize the sample and favor this transition. We further perform the deconvolution of the spectra, determining the amounts of the conversion from **Agg D** to **Agg B** and to **Agg C**, of 81% and 72%, respectively (Fig. S29). All in all, this sum of experiments clarifies the close relationships between **Agg C** and **Agg D** as well as that of **Agg B** with **Agg C**, further proving the off-pathway nature of **Agg B** toward **Agg C** and the direct involvement of **Agg C** in **Agg D** formation.

## Discussion

Control over supramolecular assembly pathways provides a powerful route to program chirality and hierarchical organization in molecular materials. In this work, a chiral π-conjugated quinquethiophene–rhodanine derivative gives rise to four distinct and isolatable supramolecular aggregates from a single enantiopure monomer under identical chemical conditions. These assemblies differ in morphology, chiroptical response, and thermodynamic stability, yet remain sufficiently long-lived to enable a detailed experimental analysis of their relationships. In this sense, there are several design features potentially responsible for the molecular origin of such interesting behavior. The A-D-A (acceptor-donor-acceptor) of the rhodanine-quinquethiophene conjugated segment promotes the formation of J-type aggregates to maximize the D-A interactions^61^. In addition, the amide functionalities enhance the cooperative character of the assembly process, favoring the formation of helical supramolecular structures. Furthermore, we hypothesize that locating the chiral center relatively far from the π-conjugated core likely reduces the efficiency of the transfer of asymmetry, making the two primary helical conformations relatively close in energy. Combined with the presence of a relatively long and flexible alkyl spacer that promotes the competition between highly directional H-bonding and π-stacking, resulting in the formation of competing structures that lead to long-lived, metastable species^62^.

By combining calorimetry, long-term kinetic measurements, spectral deconvolution, cross-seeding experiments, and controlled thermal and mechanical treatments, we experimentally mapped the interconnected supramolecular energy landscape linking these four states. This analysis reveals both on-pathway and off-pathway relationships between aggregates, including the identification of **Agg A** as a hierarchical off-pathway state that reorganizes through intermediate helices before accessing the most stable structure.

Most importantly, the revised mechanistic analysis reveals that the formation of the most stable aggregate proceeds through a helix-to-superhelix transition governed by concentration-dependent secondary nucleation. Cross-seeding experiments further demonstrate that assemblies of different dimensionality can accelerate transformations across hierarchical levels, highlighting an unusually interconnected assembly network.

Together, these results provide one of the few experimentally resolved examples in which multiple supramolecular polymorphs, their thermodynamic ordering, and their kinetic connectivity can all be directly established within a single molecular system. More broadly, this work shows how pathway selection alone can program supramolecular chirality, hierarchy, and morphology, offering new strategies for controlling complex energy landscapes in self-assembled materials.

Such mechanistic insight into hierarchical self-assembly may enable the rational design of responsive chiral materials with programmable optical, electronic, and spin-selective properties, where supramolecular structure can be tuned not by molecular redesign but by controlling the assembly pathway itself.

## Methods

All reagents and solvents for synthesis were obtained from commercial suppliers and purified or dried according to standard procedures. Column chromatography was performed on silica gel (VWR Silica 60, particle size 0.040–0.063 mm). The high-resolution mass was obtained in a MICROTOF II focus (Bruker) with an ESI (electro spray ionization) source. The software used for ion detection is Package Compass Data Analysis (Bruker Daltonics, Bremen, Germany). ^1^H and ^13^C spectra were recorded in CDCl_3_ on a Bruker Avance 400 MHz spectrometer and/or Bruker Avance III HD 500 MHz spectrometer. Solvents for spectroscopic studies were of spectroscopic grade and used as received.

**UV/Vis** measurements were performed in a conventional quartz cell (light pass 10 mm) on a Jasco J1500 Spectropolarimeter.

**Circular dichroism (CD)** spectra were acquired using a 10 mm quartz cell on a Jasco J1500 Spectropolarimeter, with 1 s integrations, 1 accumulation, and a step size of 0.5 nm with a bandwidth of 1 nm over a range of wavelengths from 300 to 800 nm at the indicated temperature in each case (Peltier).

**Ultrasonication** was performed in an Elmasonic S 60 H (Elma Schmidbauer GmbH) sonicator with 550 W power at 37 kHz.

**AFM** (NX10, Park Systems) measurements were carried out on films prepared by spin coating 50 μL of 20 μM **Agg A**–**D** solutions at 3000 rpm for 60 s onto a freshly cleaved mica substrate (1 cm diameter) in tapping mode with Multi75AL-G as a probe.

**Isothermal Titration Calorimetry (ITC)** experiments were performed at 25 °C using a Microcal VP-ITC titration microcalorimeter with a sample cell volume of 1.4 mL. The concentrations for **Agg A**–**C** were 10 μM and 5 μM for **Agg D** due to precipitation at 10 μM during the duration of the measurement. A 283 μL injection syringe was used with stirring at 155 rpm.

### High-resolution confocal microscopy

Superresolution images were acquired on a Zeiss LSM980 confocal microscope with an Airyscan2 superresolution detector. A 63×/1.4 Plan Apochromat immersion objective was used. Zen 3.8 software with Airyscan deconvolution was used to achieve below diffraction resolution.

### Powder X-Ray diffraction (PXRD)

**Agg A**–**D** were prepared following the described protocols in Section 3 of the Supplementary Information (20 µM, 10% DCE/MCH), and the CD signal was confirmed prior to measuring. The samples were deposited onto a zero-background silicon holder (Silicon low background sample holder for small sample amounts, Ø 51.5 mm, Ø 25 mm Si crystal, Bruker). Powder X-ray diffraction (PXRD) patterns were collected over the 2θ range of 5–40° using a step size of 0.016° and a counting time of 1.5 s per step. Measurements were performed on a Bruker D8 Advance powder diffractometer equipped with Cu Kα radiation and a LynxEye position-sensitive (PSD) rapid detector.

## Supplementary information

Supplementary Information

Description of Additional Supplementary Information

Supplementary Video S1

Supplementary Video S2

Transparent Peer Review file

## Data Availability

The data generated in this study have been deposited in a GitHub database https://github.com/hermanslab/4ChiralAgg. The data structure follows the numbering of the figures in the main text and Supplementary Information. All data are available from the corresponding author upon request.
