# Supplementary material for: From helices to superhelices through hierarchical assembly across competing pathways
Source: Nat Commun. 2026 Aug 11;17:9619. doi: 10.1038/s41467-026-76656-4 (PMC13554182; doi:10.1038/s41467-026-76656-4)
Supplement: Supplementary file 1 [file 41467_2026_76656_MOESM1_ESM.pdf]

SUPPLEMENTARY INFORMATION

From Helices to Superhelices through Hierarchical Assembly across Competing Pathways

Dr. Kyeong-Im Hong,[a,b,c,#] Dr. Jorge S. Valera,[b,d,#] Dr. Ana M. Garcia,[c,e] Prof. Thomas M. Hermans,[b*] and Dr. Amparo Ruiz-Carretero[a,c*]

[a]Instituto de Ciencia de Materiales de Madrid, Consejo Superior de Investigaciones Científicas CSIC, Sor Juana Inés de la Cruz 3, 28049, Madrid, Spain

[b]IMDEA Nanociencia, C/ Faraday 9, 28049, Madrid, Spain

[c]University of Strasbourg, Institute Charles Sadron, CNRS, UPR22, 23 Rue du Loess, 67034, Strasbourg Cedex 2, France

[d]Facultad de Ciencias, Universidad Autónoma de Madrid, Francisco Tomás y Valiente 7, 28049, Madrid, Spain

[e]Instituto Regional de Investigación Científica Aplicada (IRICA), Universidad de Castilla-La Mancha, 13071 Ciudad Real, Spain; Faculty of Chemical Science and Technology, Universidad de Castilla-La Mancha, 13071 Ciudad Real, Spain

#Equal contribution

*Corresponding authors: amparo.ruiz@csic.es, thomas.hermans@imdea.org

Table of contents

1. Materials and Methods **S1**

2. Synthesis **S2**

3. Preparation protocols of Agg A-D **S3**

4. Supplementary figures **S5**

5. ITC experiment's description **S12**

6. Description of spectral deconvolutions **S24**

7. Seeding conversion experiments **S31**

8. Supporting video captions **S35**

1. Materials and Methods

All reagents and solvents for synthesis were obtained from commercial suppliers and purified or dried according to standard procedures. Column chromatography was performed on silica gel (VWR Silica 60, particle size 0.040–0.063 mm). The high-resolution mass was obtained in a MICROTOF II focus (Bruker) with an ESI (electro spray ionization) source. The software used for ion detection is Package Compass Data Analysis (Bruker Daltonics, Bremen, Germany). ^{1}H and ^{13}C spectra were recorded in $CDCl_3$ on a Bruker Avance 400 MHz spectrometer and/or Bruker Avance III HD 500 MHz spectrometer. Solvents for spectroscopic studies were of spectroscopic grade and used as received. Solvents for spectroscopic studies were of spectroscopic grade and used as received.

UV/Vis measurements were performed in a conventional quartz cell (light pass 10 mm) on a Jasco J1500 Spectropolarimeter.

Circular dichroism (CD) spectra were acquired using a 10 mm quartz cell on a Jasco J1500 Spectropolarimeter, with 1 s integrations, 1 accumulation and a step size of 0.5 nm with a bandwidth of 1 nm over a range of wavelengths from 300 to 800 nm at the indicated temperature in each case (Peltier).

Ultrasonication was performed in an Elmasonic S 60 H (Elma Schmidbauer GmbH) sonicator with 550 W power at 37 kHz.

AFM (NX10, Park Systems) measurements were carried out on films prepared by spin coating 50 μL of 20 μM **Agg A-D** solutions at 3000 rpm for 60 seconds onto a freshly cleaved mica substrates (1 cm diameter) in tapping mode with Multi75AL-G as a probe.

Isothermal Titration Calorimetry (ITC) experiments were performed at 25 °C using a Microcal VP-ITC titration microcalorimeter with a sample cell volume of 1.4 mL. The concentrations for **AggA-C** were 10 μM and 5 μM for **Agg D** due to precipitation at 10 μM during the duration of the measurement. A 283 μL injection syringe was used with stirring at 155 rpm.

High-resolution confocal microscopy

Superresolution images were acquired on a Zeiss LSM980 confocal microscope with an Airyscan2 superresolution detector. A 63x/1.4 Plan Apochromat immersion objective was used. Zen 3.8 software with Airyscan deconvolution was used to achieve below diffraction resolution.

Powder X-Ray Diffraction (PXRD)

Agg A-D were prepared following the described protocols in section 2 of thia supporting information (20 μM, 10% DCE/MCH) and the CD signal was confirmed prior measuring. The samples were deposited onto a zero-background silicon holder (Silicon low background sample holder for small sample amounts, Ø 51.5mm, Ø 25mm Si cristal, Bruker). Powder X-ray diffraction (PXRD) patterns were collected over the 2θ range of 5–40° using a step size of 0.016° and a counting time of 1.5 s per step. Measurements were performed on a Bruker D8 Advance powder

diffractometer equipped with Cu Kα radiation and a LynxEye position-sensitive (PSD) rapid detector.

2. Synthesis

Synthesis of QRD and QRL

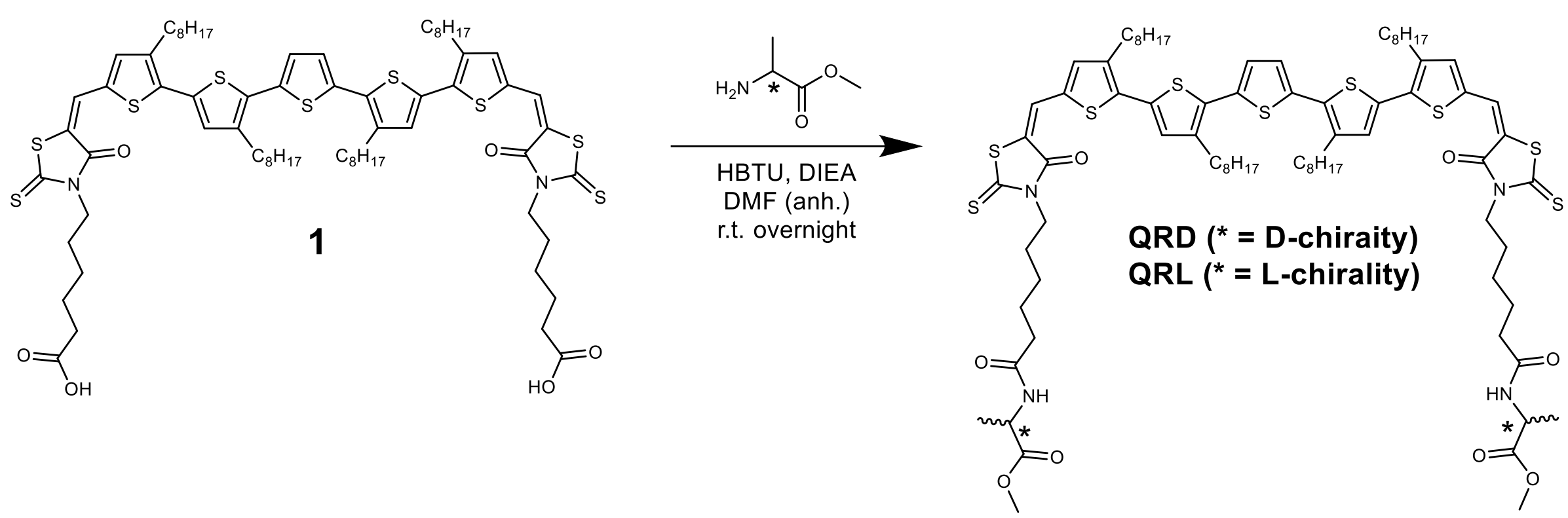

Figure S1. Synthetic route towards **QRD** and **QRL**.

Polythiophene **1** was prepared as previously reported.[1]

Quinquethiophene **1** (150 mg, 0.109 mmol, 1 equiv.), the L- or D-alanine methyl ester hydrochloride (0.0.327 mmol, 3 equiv.) and HBTU were dissolved in dry DMF (3 mL) and stir for 5 minutes under argon atmosphere. Then, diisopropylethylamine (114 μL, 0.654 mmol, 6 equiv.) was added and the mixture was left to react at room temperature overnight. After that time, $CHCl_3$ was added (50 ml) and the crude of reaction was washed with HCl 1 M, brine and distilled water. Then, the organic layer was dried over anhydrous $MgSO_4$. The solvent was eliminated under reduced pressure and the crude was purified by column chromatography in a mixture chloroform/ethanol, starting with 100% chloroform until chloroform/ethanol (20:1). Then, the fraction obtained from the column was precipitate with cold acetone to obtain a crystalline solid of the final compounds **QRD** and **QRL**.

Compound QRD (D-Ala): purple solid (125 mg, 74% yield).

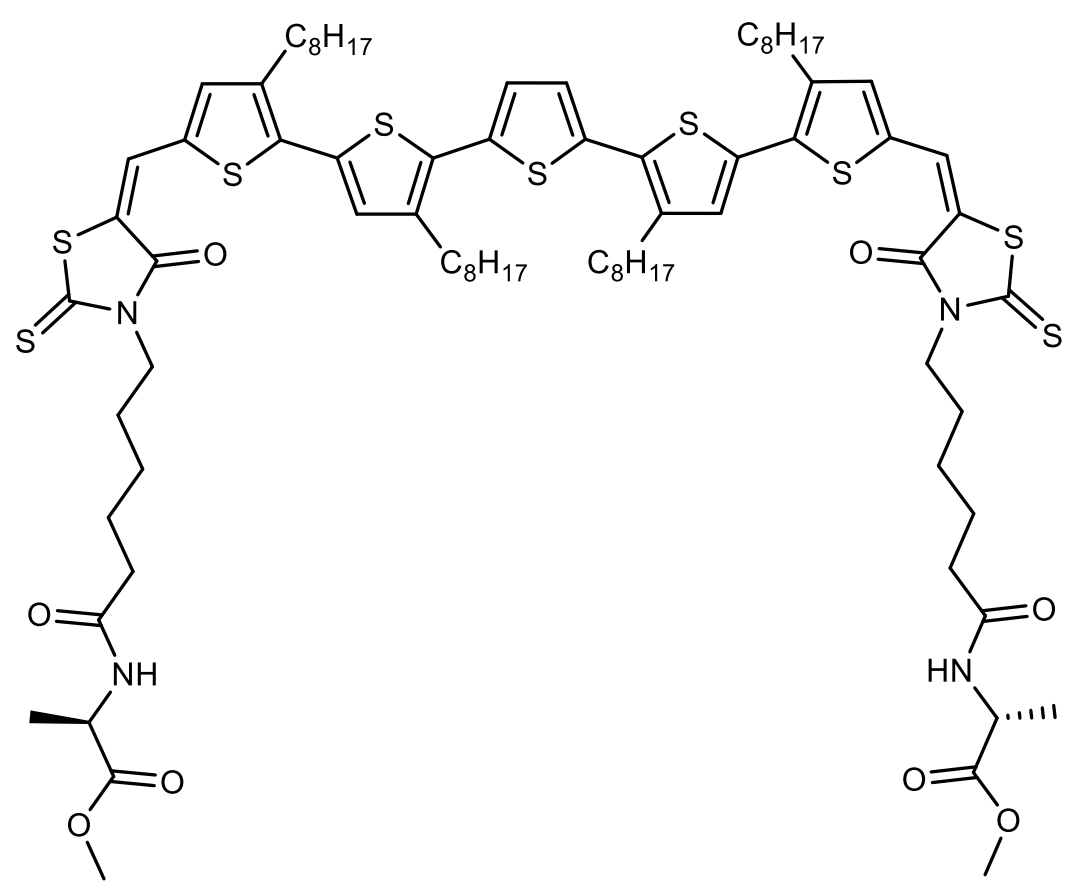

^{1}H NMR (400 MHz, $CDCl_3$) δ: 7.80 (s, 1H, H-double bond), 7.26 (s, 2H, H-thiophene), 7.17 (s, 2H, H-thiophene), 7.15 (s, 2H, H-thiophene), 6.03 (s, 2H, *J* = 7.5 Hz, NH amide), 4.63 (p, 2H, *J* = 7.2 Hz, 1H, Hα alanine), 4.14 (t, *J* = 7.6 Hz, 4H, rhodanine-CH_2), 3.78 (s, 6H, OCH_3 alanine), 2.91 – 2.78 (m, 8H, 4 x CH_2 aliphatic chain thiophenes), 2.31 – 2.20 (m, 4H, CH_2-CONH), 1.84 – 1.59 (m, 16H, aliphatic chain rhodanine CH_2, aliphatic chain thiophenes), 1.51 – 1.22 (m, 51H, CH-CH_3 alanine, aliphatic chain rhodanine CH_2, aliphatic chain thiophene), 0.98 – 0.85 (m, 12H, 4 x CH_3 aliphatic chain thiophenes).

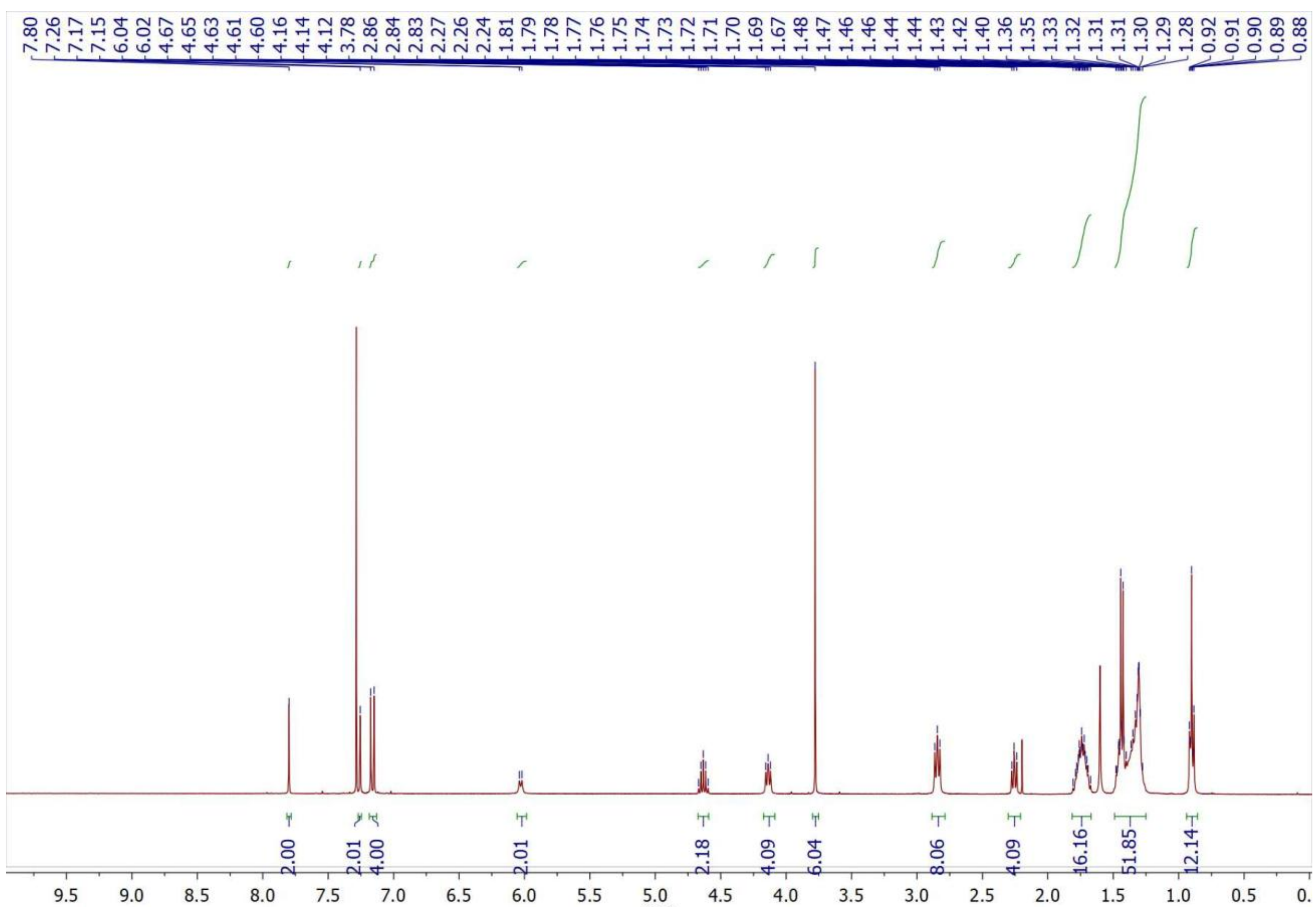

^{13}C-NMR (100 MHz, $CDCl_3$) δ: 192.3 (C=S), 173.7 (C=O), 172.2 (C=O), 167.5 (C=O), 141.1, 140.5, 139.7, 137.5, 135.8, 135.1, 133.0, 132.2, 130.0, 126.5, 125.1, 120.4 (2 x C=C, 10 x C-thiophene), 52.5, 47.9, 44.5, 36.2, 31.9, 31.8, 30.6, 30.3, 29.7, 29.6, 29.5, 29.4, 29.3, 29.3, 26.7, 26.2, 25.0, 22.7, 22.6, 18.6, 14.2 (OC̲H_3 alanine, C̲H-C̲H_3 alanine, 5 x C aliphatic chain rhodanine, 16 x C aliphatic chain thiophenes).

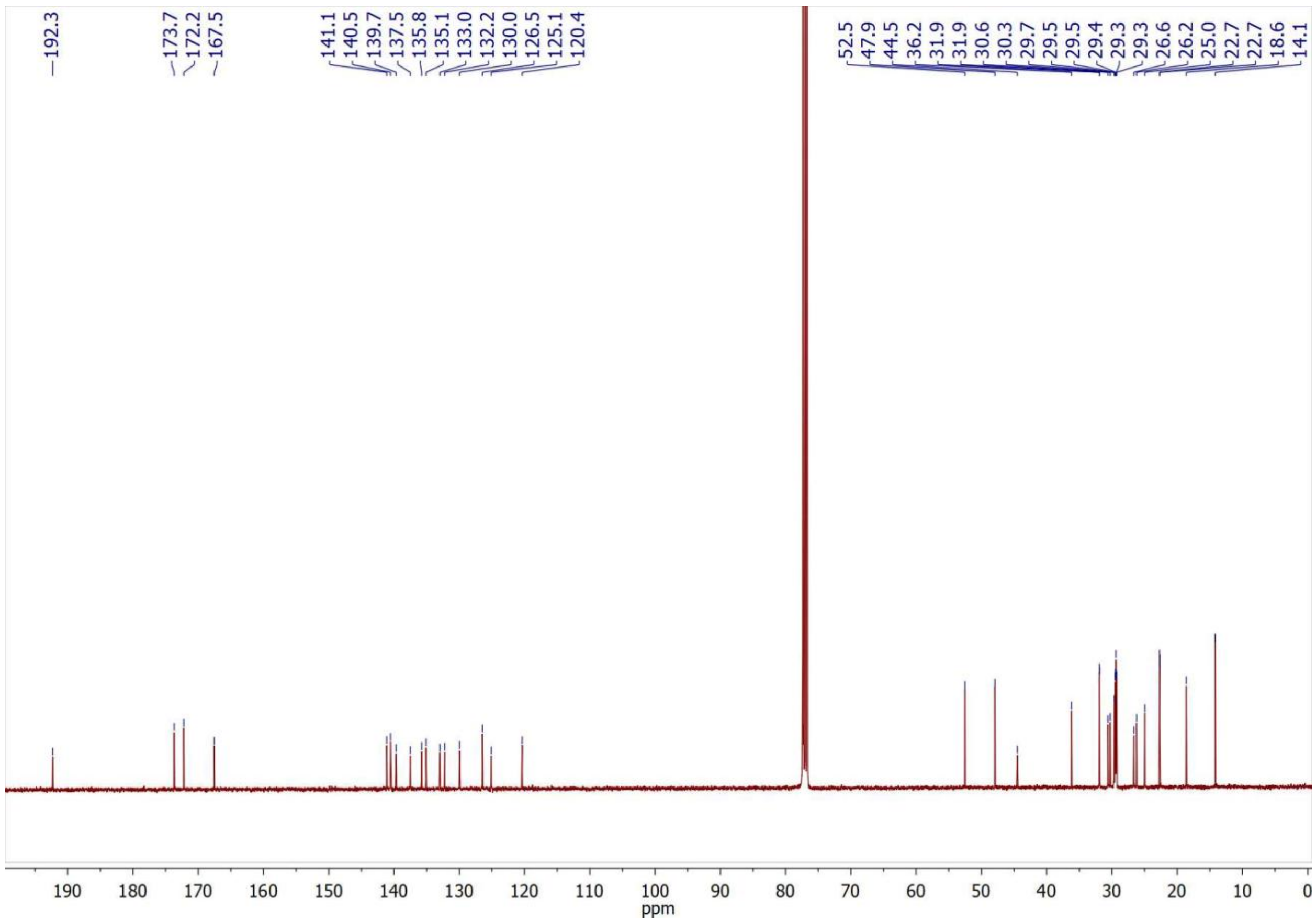

HRMS (ESI+) m/z: 1544.5952 (MH+) ($C_{80}H_{112}N_4O_8S_9$ requires 1544.5961)

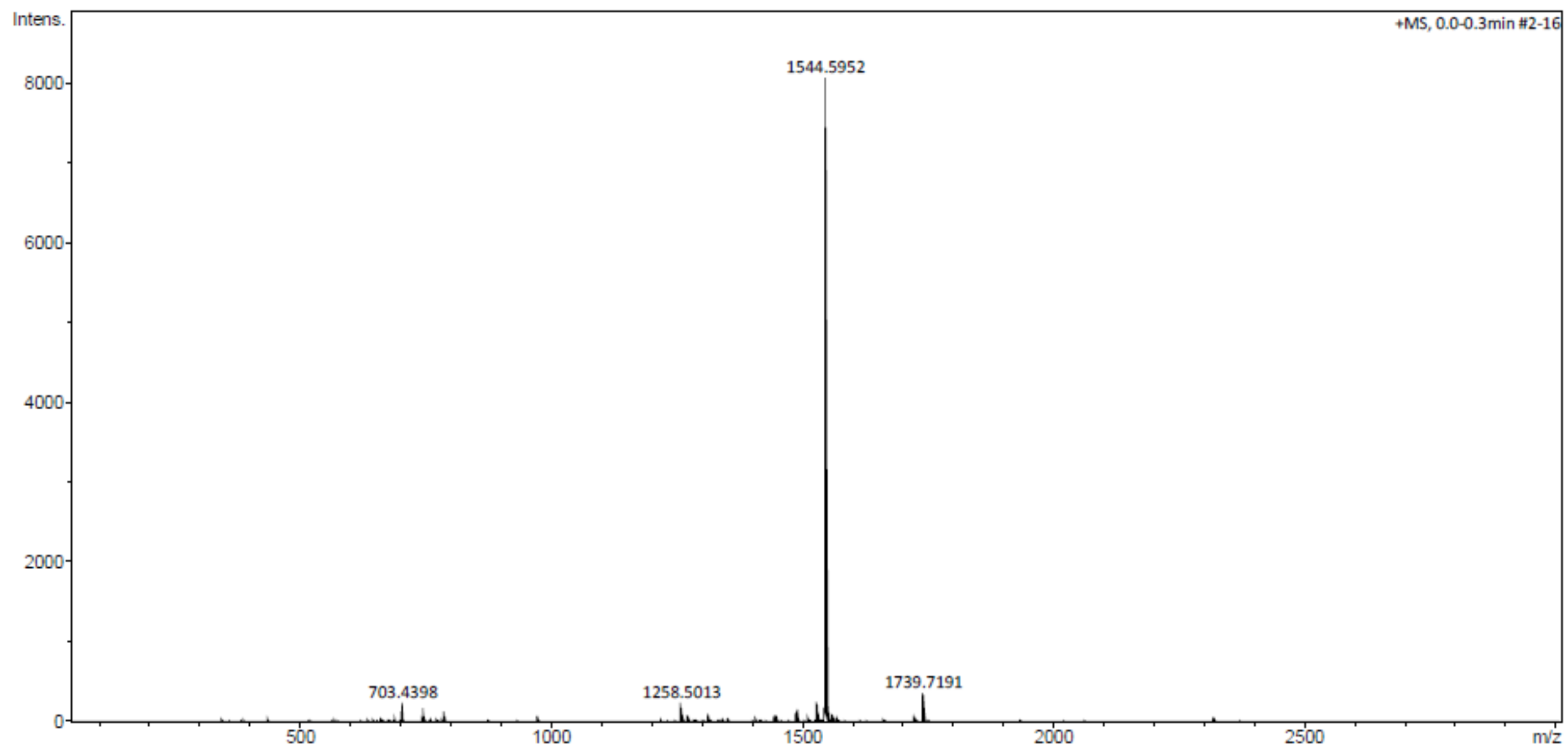
Intens.
+MS, 0.0-0.3min #2-16
1544.5952
703.4398
1258.5013
1739.7191
8000
6000
4000
2000
0
500
1000
1500
2000
2500
m/z

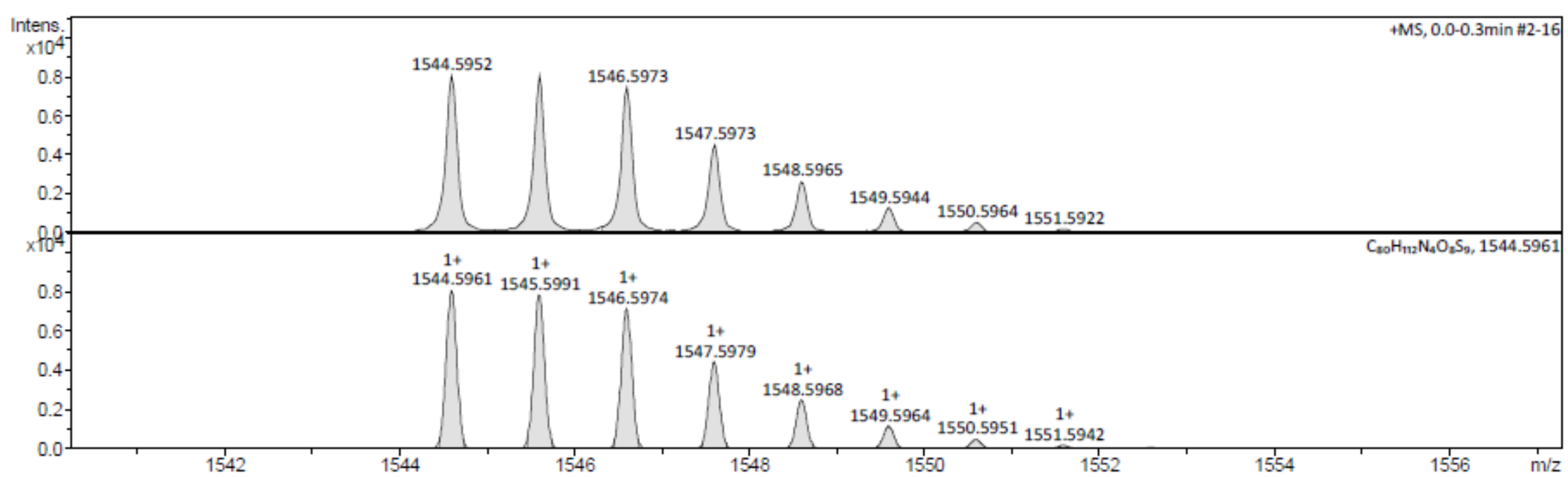
Intens.
x10^4
+MS, 0.0-0.3min #2-16
1544.5952
1546.5973
1547.5973
1548.5965
1549.5944
1550.5964
1551.5922
C80H112N4O8S9, 1544.5961
1+
1544.5961
1+
1545.5991
1+
1546.5974
1+
1547.5979
1+
1548.5968
1+
1549.5964
1+
1550.5951
1+
1551.5942
0.8
0.6
0.4
0.2
0.0
1542
1544
1546
1548
1550
1552
1554
1556
m/z

<u>Compound QRL (L-Ala):</u> purple solid (108 mg, 64% yield).

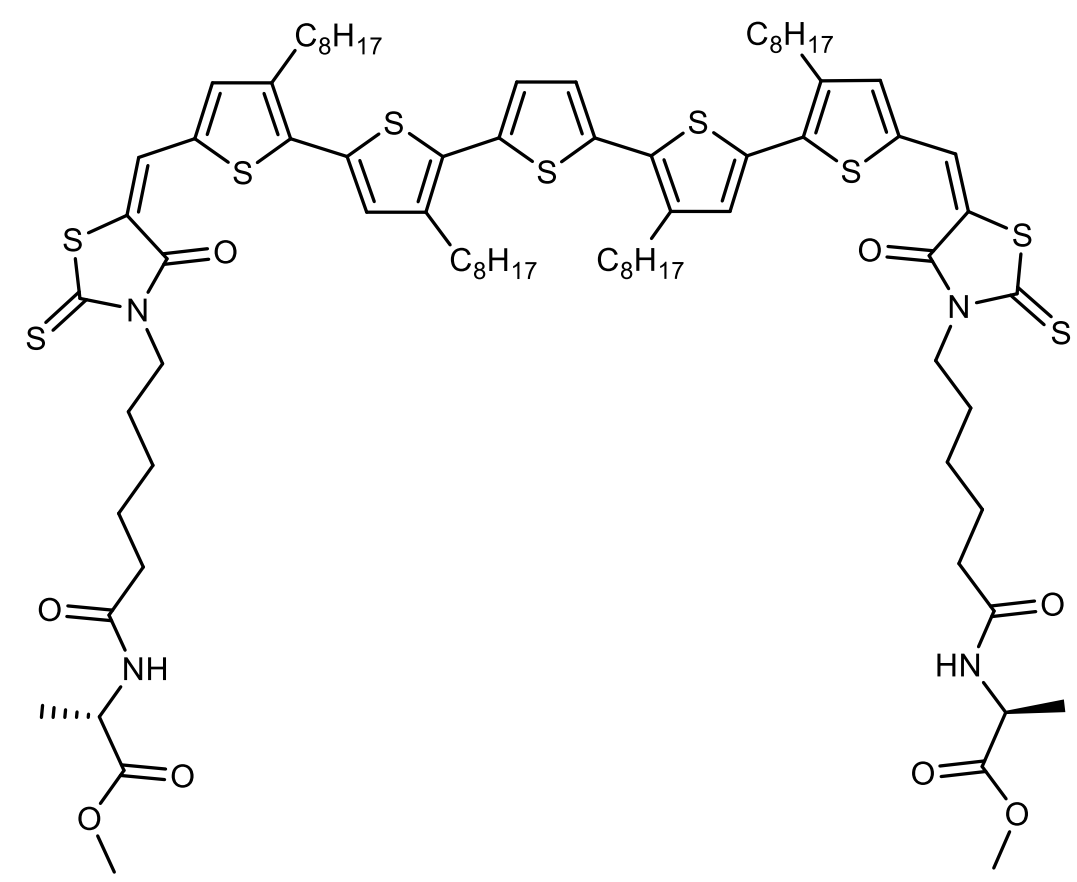

^{1}H NMR (400 MHz, $CDCl_3$) δ: 7.79 (s, 1H, H-double bond), 7.25 (s, 2H, H-thiophene), 7.17 (s, 2H, H-thiophene), 7.14 (s, 2H, H-thiophene), 6.05 (s, 2H, *J* = 7.5 Hz, <u>NH</u> amide), 4.63 (p, 2H, *J* = 7.2 Hz, 1H, Hα alanine), 4.13 (t, *J* = 7.6 Hz, 4H, rhodanine-<u>CH_2</u>), 3.78 (s, 6H, O<u>CH_3</u> alanine), 2.91 – 2.77 (m, 8H, 4 x <u>CH_2</u> aliphatic chain thiophenes), 2.31 – 2.20 (m, 4H, <u>CH_2</u>-CONH), 1.84 – 1.59 (m, 16H, aliphatic chain rhodanine <u>CH_2</u>, aliphatic chain thiophenes), 1.51 – 1.22 (m, 51H, CH-<u>CH_3</u> alanine, aliphatic chain rhodanine <u>CH_2</u>, aliphatic chain thiophene), 0.98 – 0.85 (m, 12H, 4 x <u>CH_3</u> aliphatic chain thiophenes).

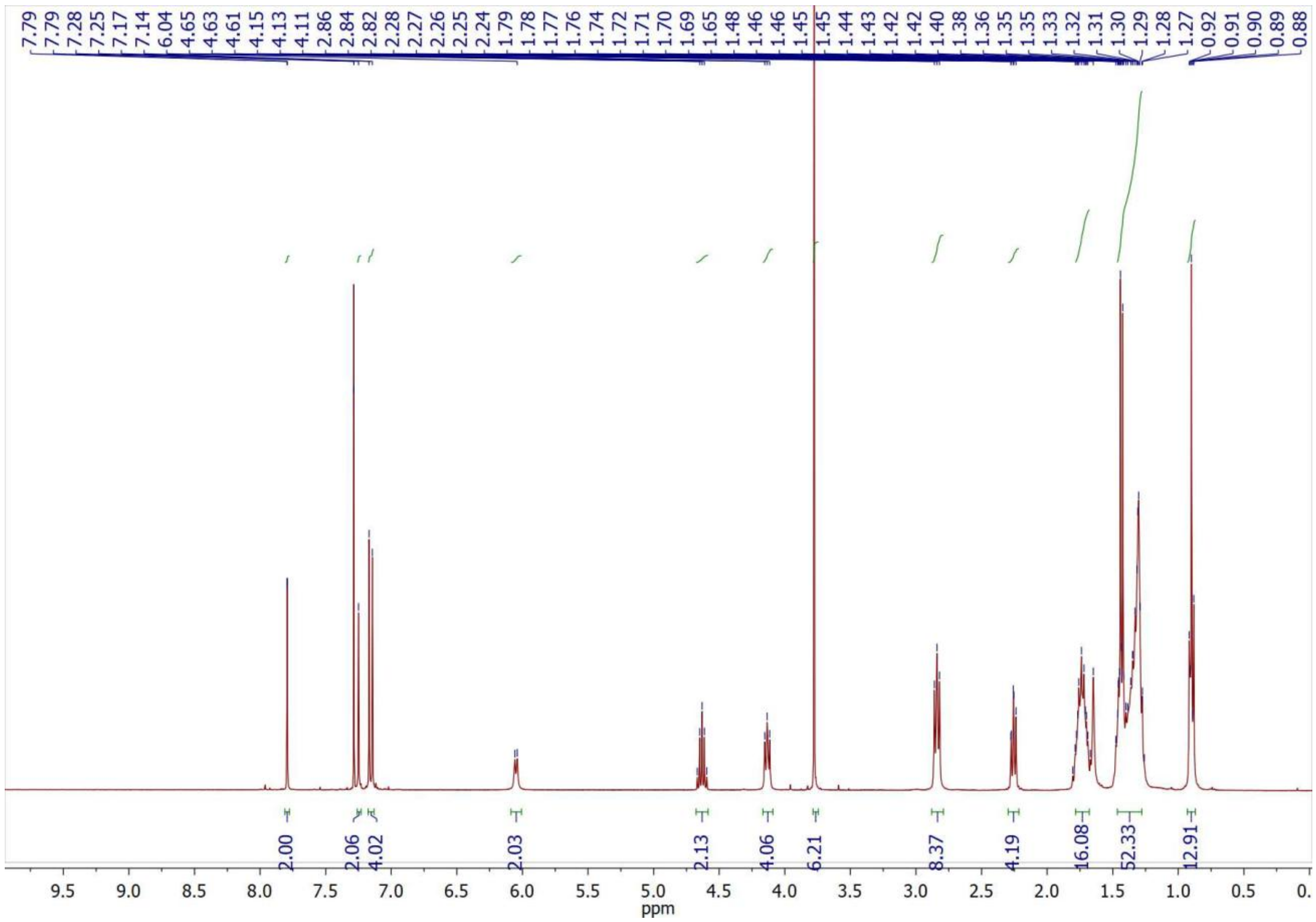

^{13}C-NMR (100 MHz, $CDCl_3$) δ: 192.3 (C=S), 173.7 (C=O), 172.2 (C=O), 167.5 (C=O), 141.1, 140.5, 139.7, 137.5, 135.8, 135.1, 133.0, 132.2, 130.0, 126.5, 125.1, 120.4 (2 x C=C, 10 x C-thiophene), 52.5, 47.9, 44.5, 36.2, 31.9, 31.8, 30.6, 30.3, 29.7, 29.6, 29.5, 29.4, 29.3, 29.3, 26.7, 26.2, 25.0, 22.7, 22.6, 18.6, 14.2 (O<u>C</u>H_3 alanine, <u>C</u>H-<u>C</u>H_3 alanine, 5 x C aliphatic chain rhodanine, 16 x C aliphatic chain thiophenes).

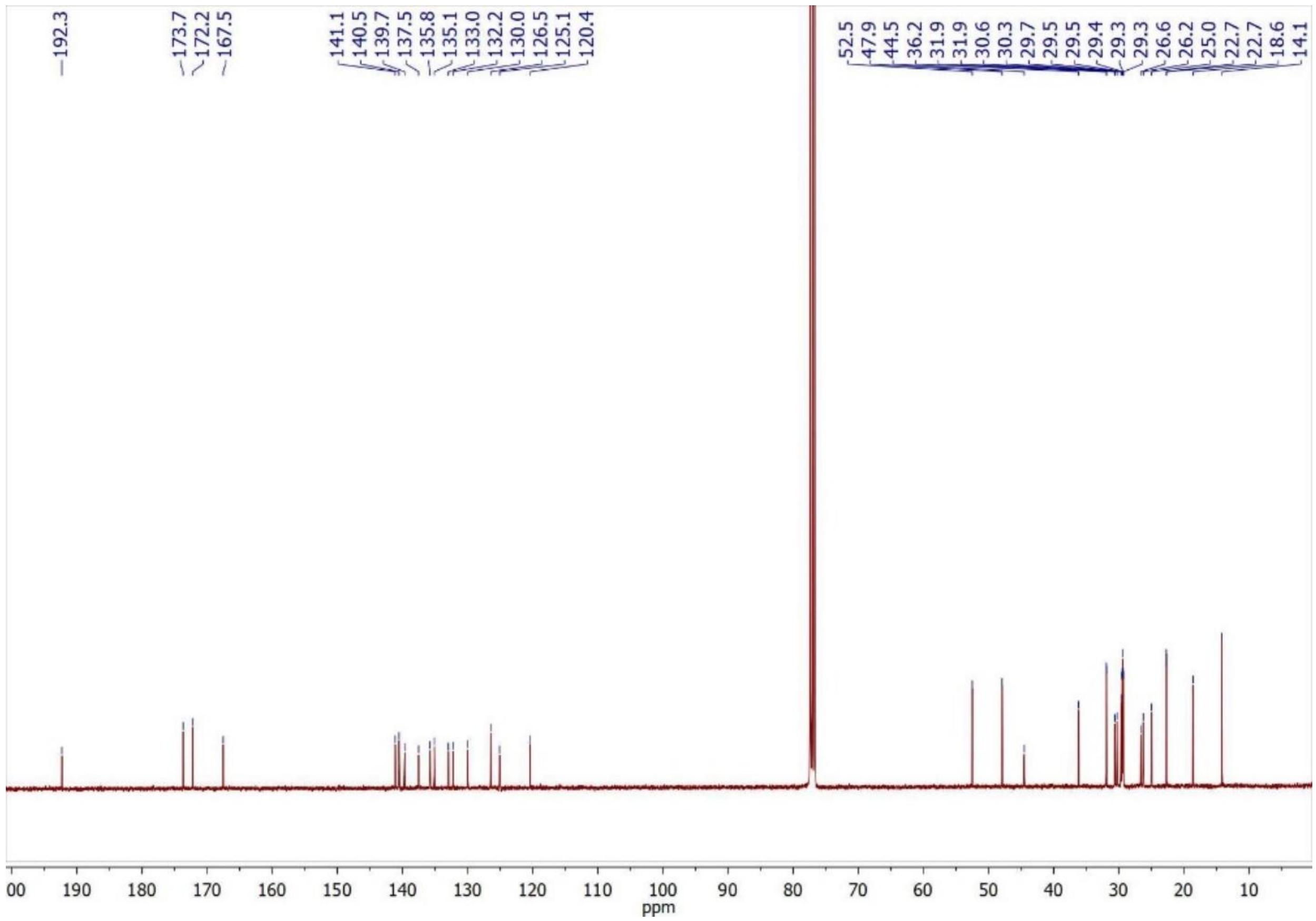

HRMS (ESI+) m/z: 1567.5830 (M+Na) ($C_{80}H_{112}N_4O_8S_9Na$ requires 1567.5859)

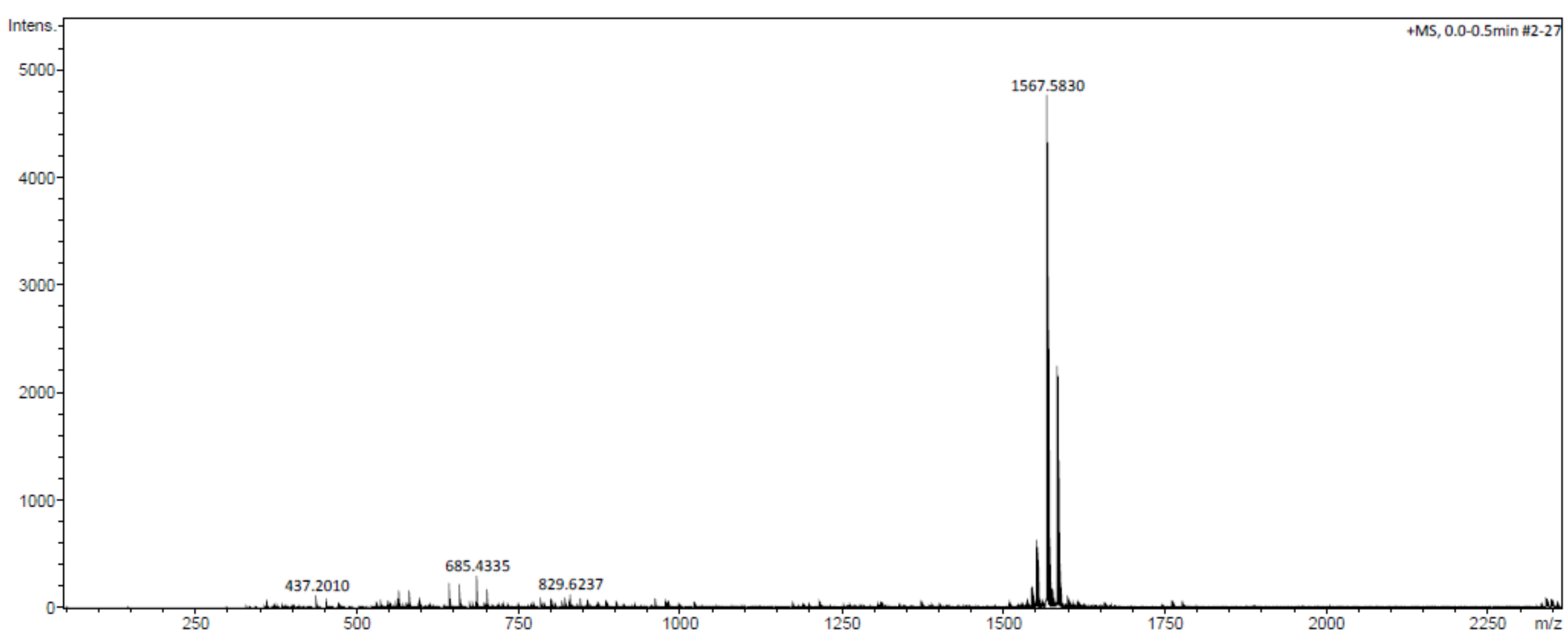

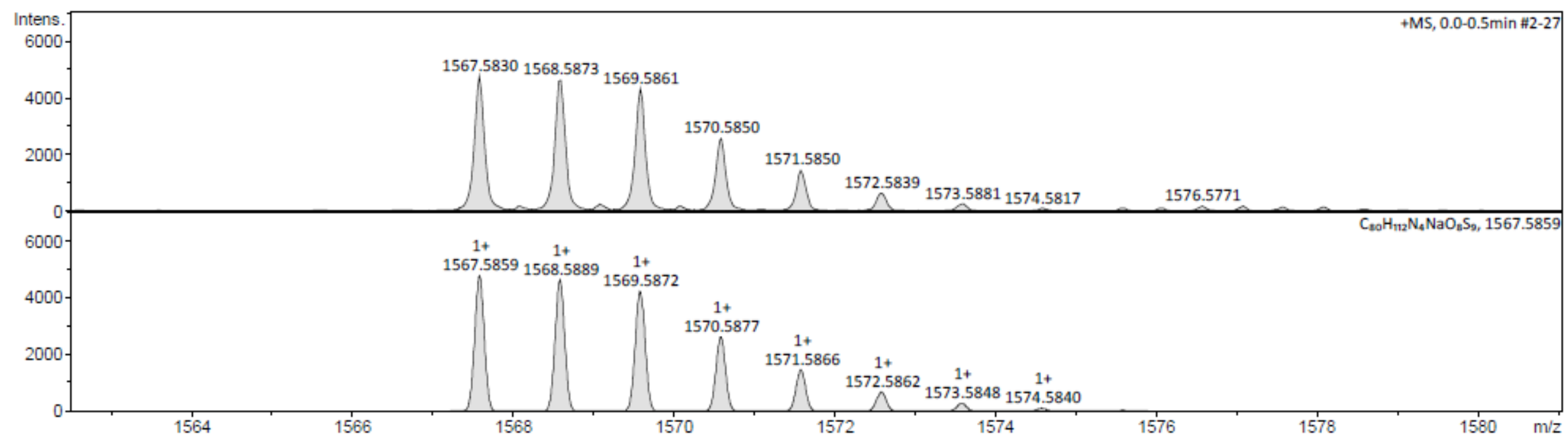

3. Preparation Protocols of Agg A-D

Aggregate A

200 µL of a 200 µM **QRD** stock solution in dichloroethane (DCE) were injected into 1800 µL of methylcyclohexane (MCH) until reaching a 20 µM 1:9 DCE/MCH solution. Then, the 20 µM 1:9 DCE/MCH solution was sonicated for 30 minutes at room temperature, resulting in a deep blue solution. Ice cubes were added into the ultrasonication bath to avoid heating during sonication. The temperature was followed with a thermometer placed directly in the ultrasonication bath.

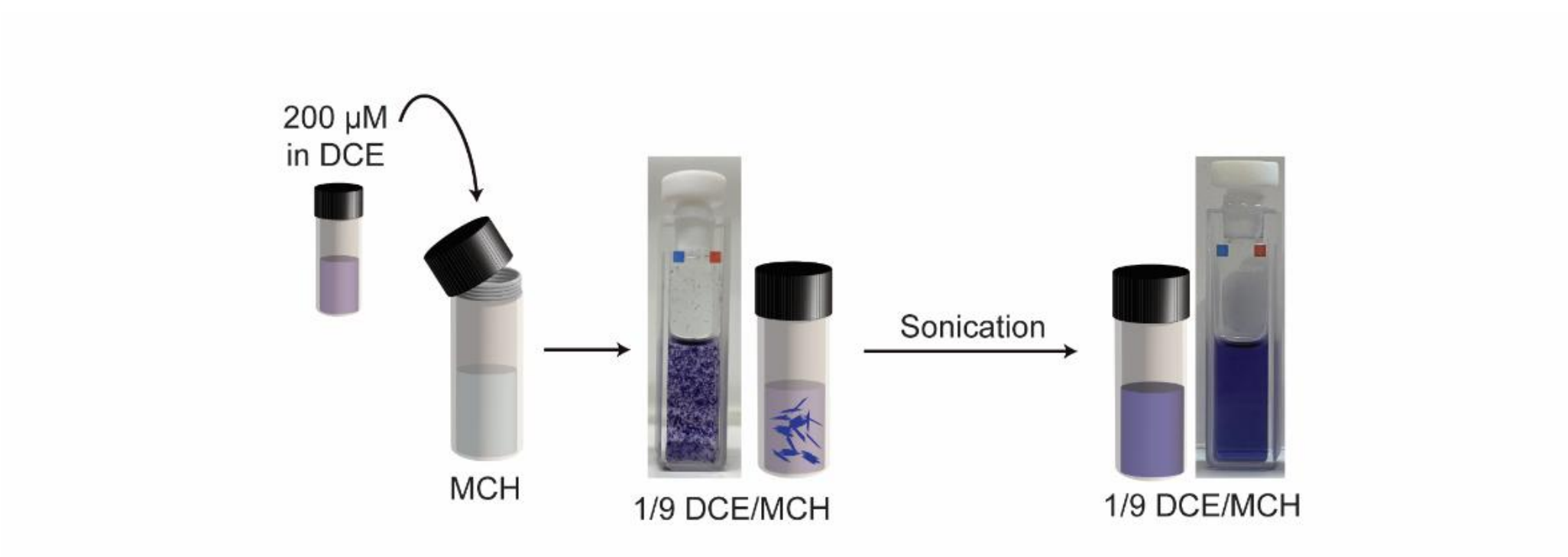

Aggregate B

A 20 µM 1:9 DCE/MCH **QRD** solution was warmed up to 70 °C to reach the molecularly dissolved state for 15 min, obtaining a bright red solution. Then, the hot solution was directly placed in the ultrasound bath and sonicated for 30 minutes. The solution reached room temperature during this time while being sonicated, resulting in a purple solution.

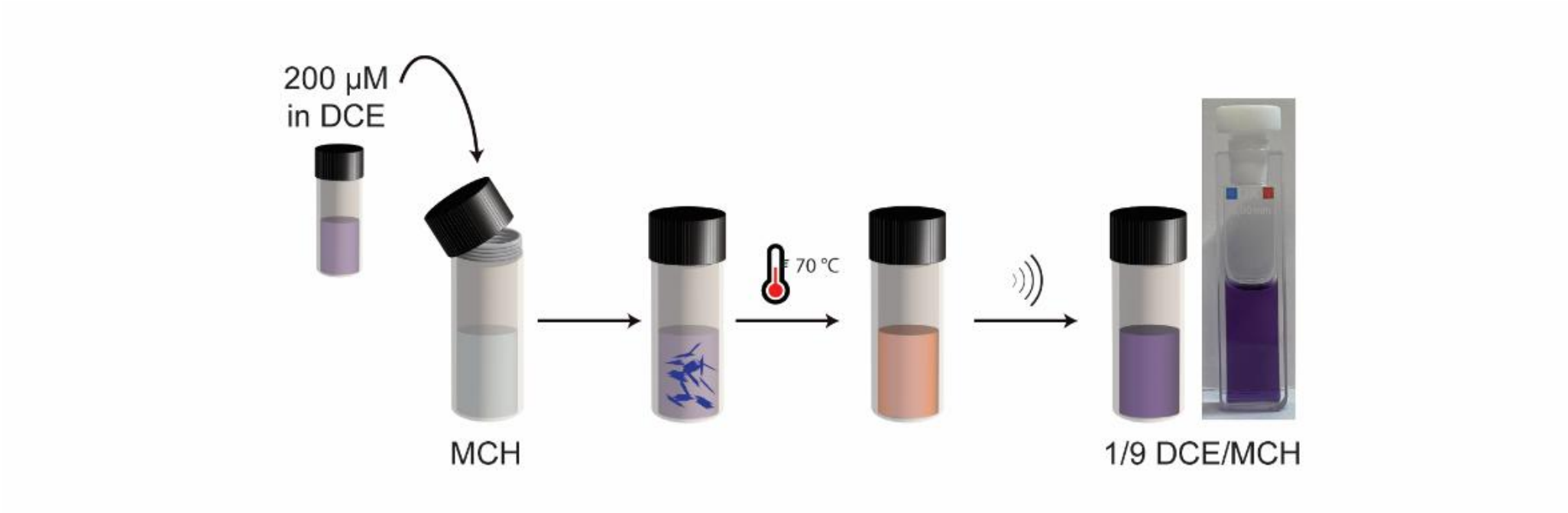

Aggregate C

A 20 μM 1:9 DCE/MCH **QRD** solution was warmed up to 70 °C to reach the molecularly dissolved state, obtaining a bright red solution. Then, the hot solution was placed in an ice bath (fast cooling) for 30 minutes. After this time, a precipitate was observed. The solution with the precipitate was then sonicated for 30 minutes at room temperature, resulting in a purple solution.

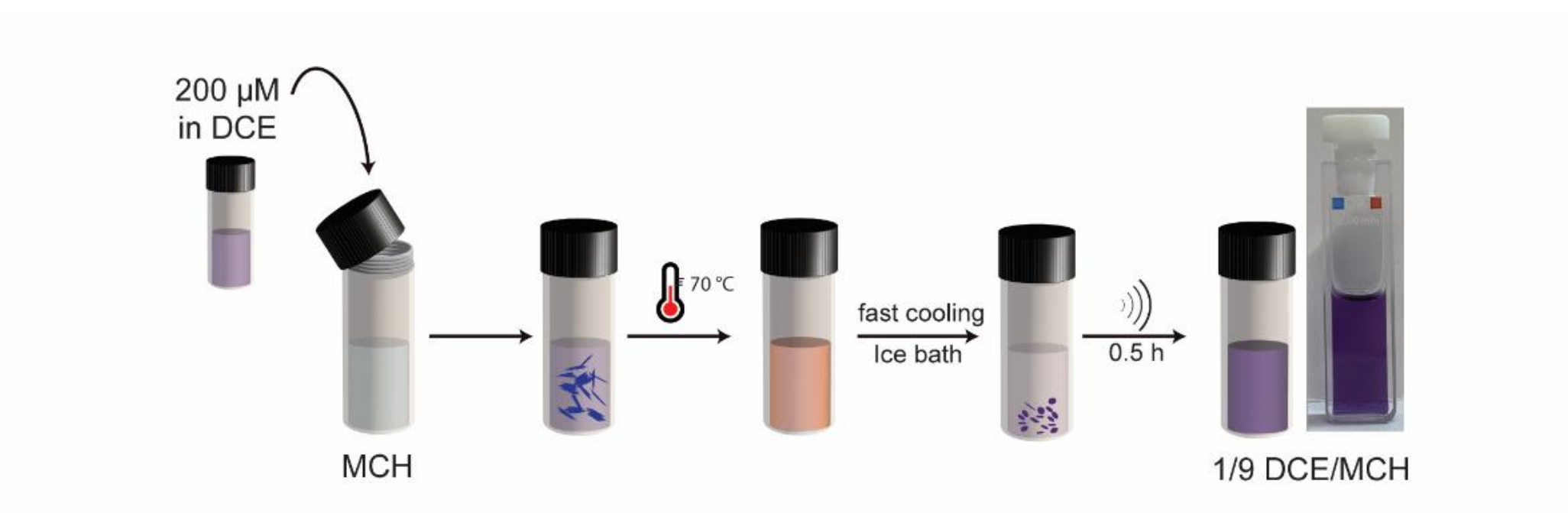

Aggregate D

A 20 μM 1:9 DCE/MCH **QRD** solution was warmed up to 70 °C to reach the molecularly dissolved state, obtaining a bright red solution. Then, the hot solution was cooled down to 15 °C at a 1 K min^{-1} cooling rate, resulting in a purple solution with the precipitation of supramolecular polymers.

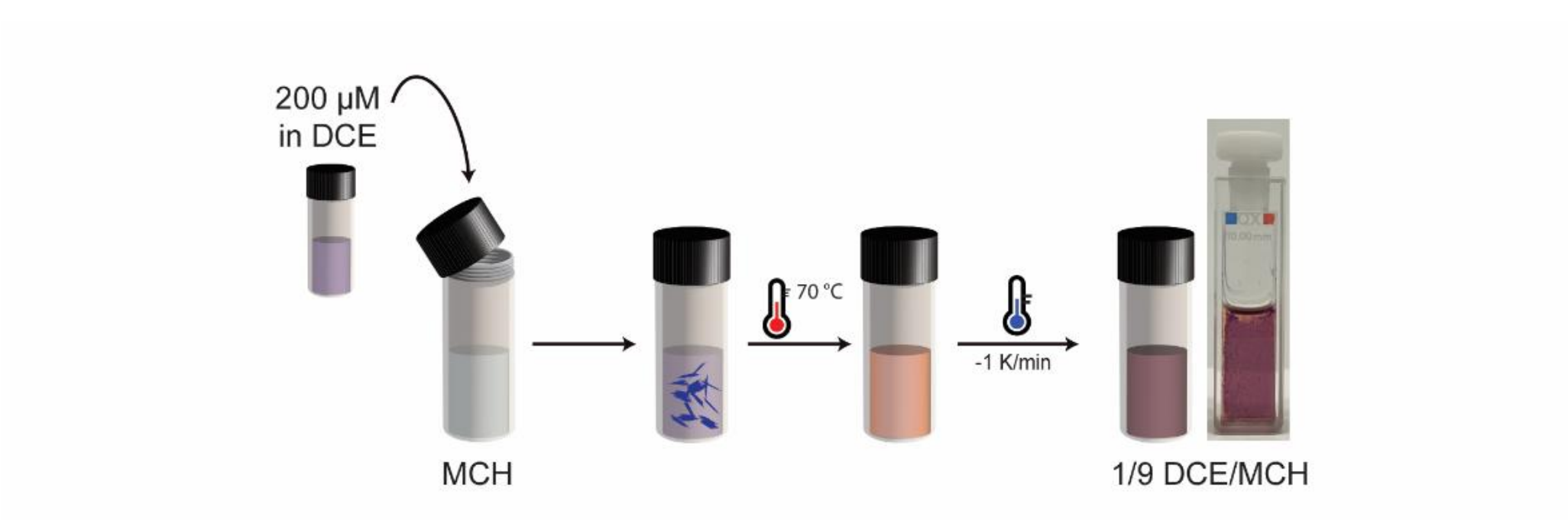

4. Supplementary Figures

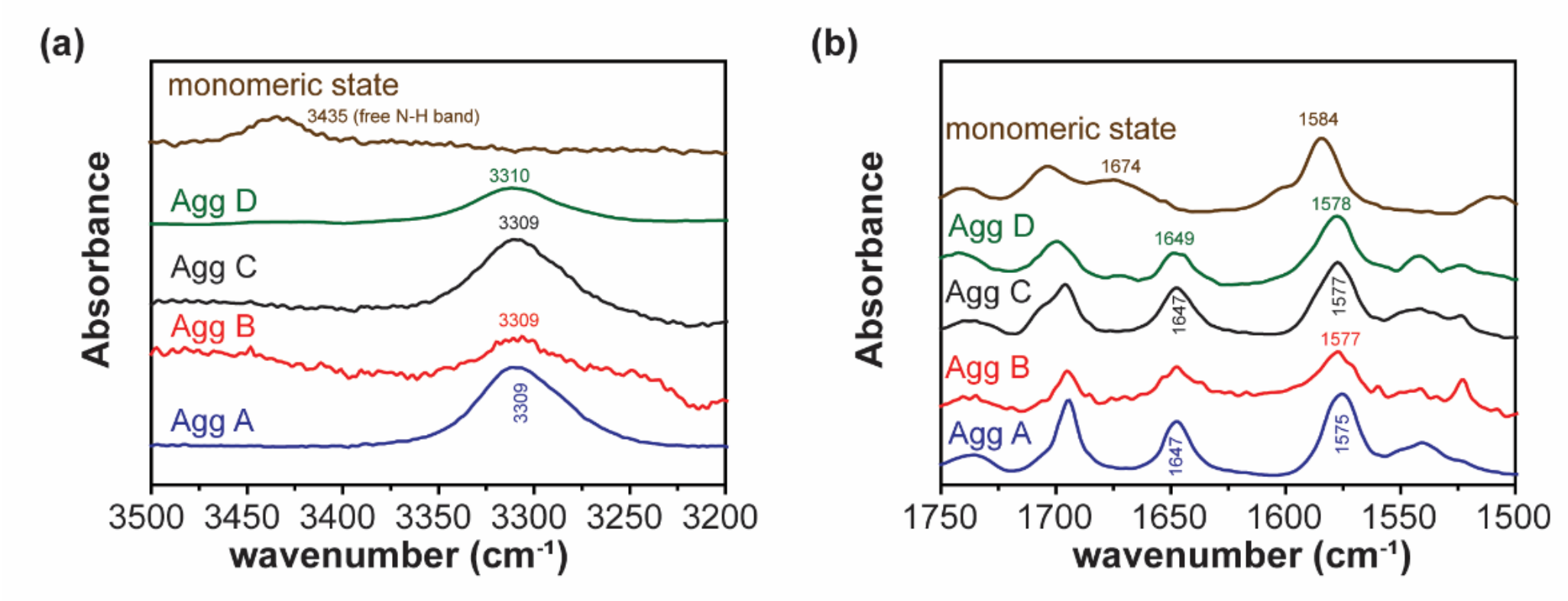

Figure S2. Partial FT-IR spectra of **QRD** (500 μM) in $CDCl_3$ solution, and of **Agg A-D** on thin films prepared by drop casting 20 μM 1:9 DCD/MCH solutions. a) N-H stretching bands (Amide I), and b) C=O stretching (1630 – 1690 cm^{-1}), N-H bending and C-N stretching (1510 – 1580 cm^{-1}) bands (Amide II).

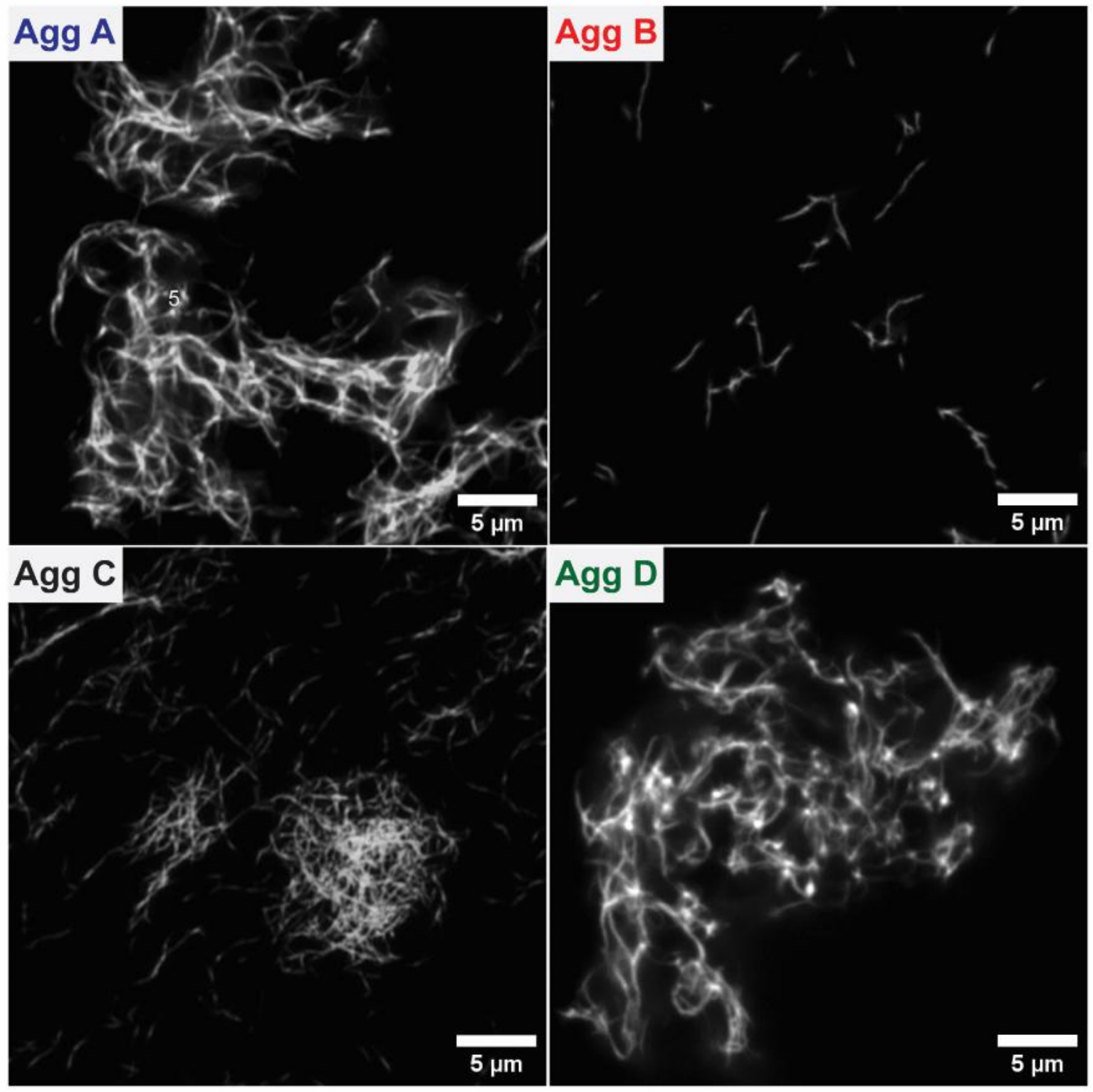

Figure S3. Super resolution confocal microscopy images of **Agg A-D** (20 µM,1:9 DCE/MCH) loaded on concave microscopy glass substrates.

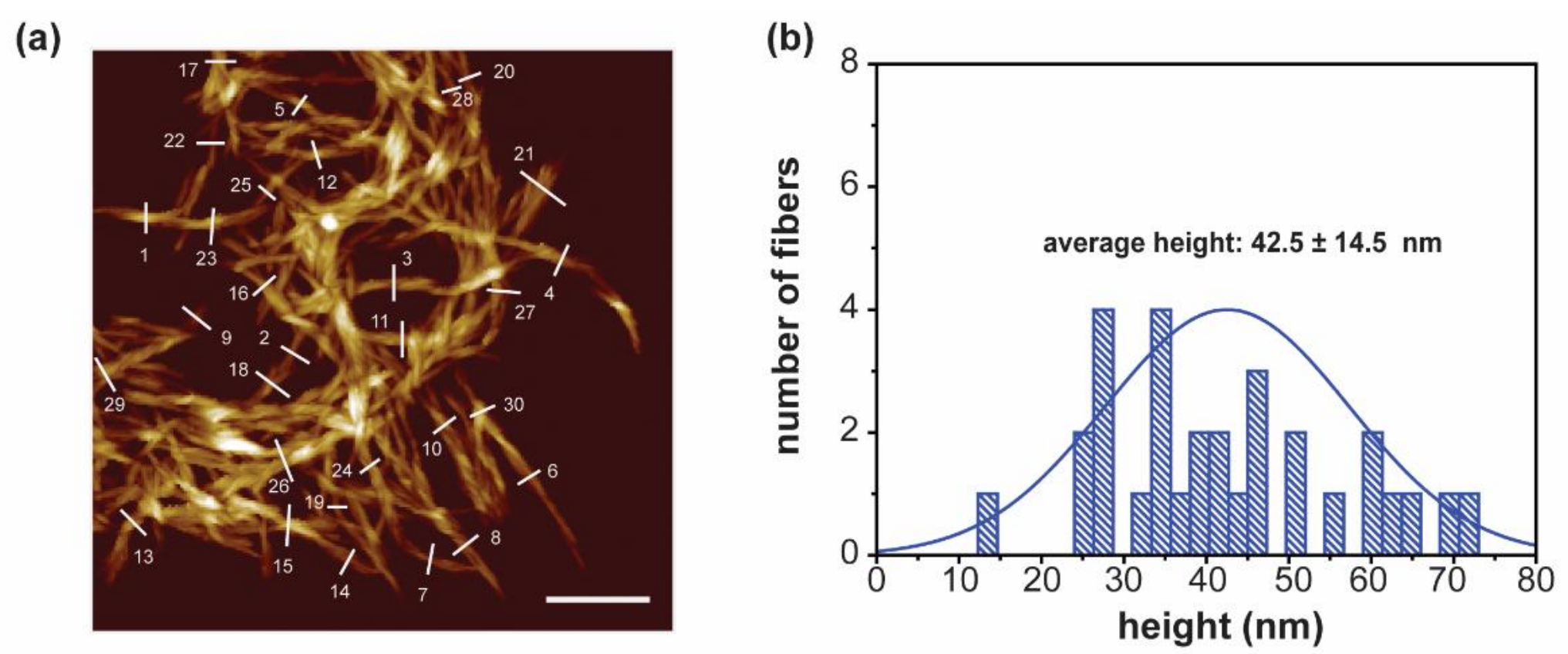

Figure S4. AFM images analysis of **Agg A** (20 µM, 1/9 DCE/MCH) spin-coated on mica (50 µL at 3000 rpm, 60 s). The numbered points of a) height of the supramolecular polymers, and b) corresponding height profiles of the fibers (scale bar: 1 µm).

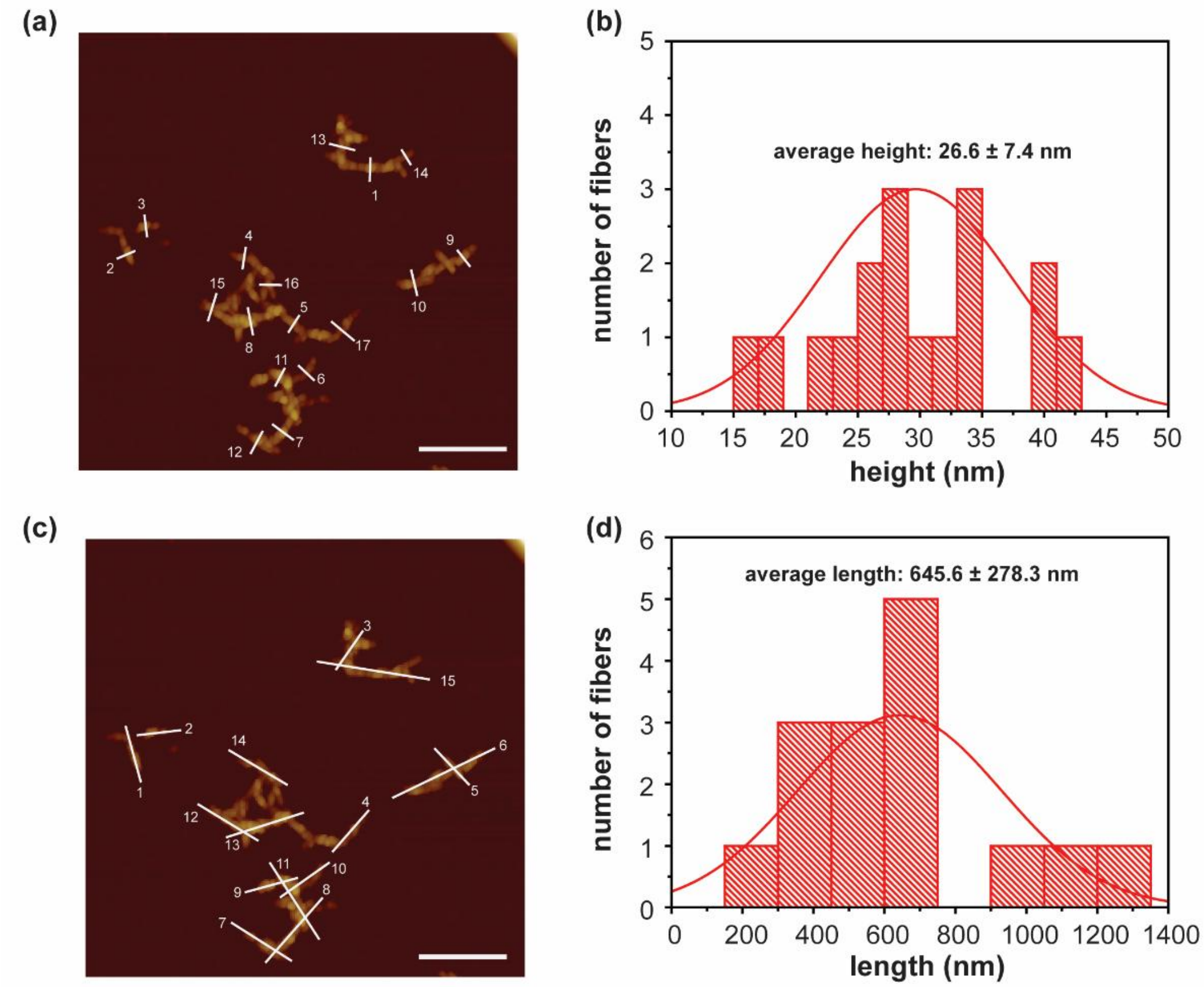

Figure S5. AFM images analysis of **Agg B** (20 μM, 1/9 DCE/MCH) spin-coated on mica (50 μL at 3000 rpm, 60 s). The numbered points of a) height and c) length of the supramolecular polymers and, corresponding b) height profiles and d) length of the fibers (scale bar: 1 μm).

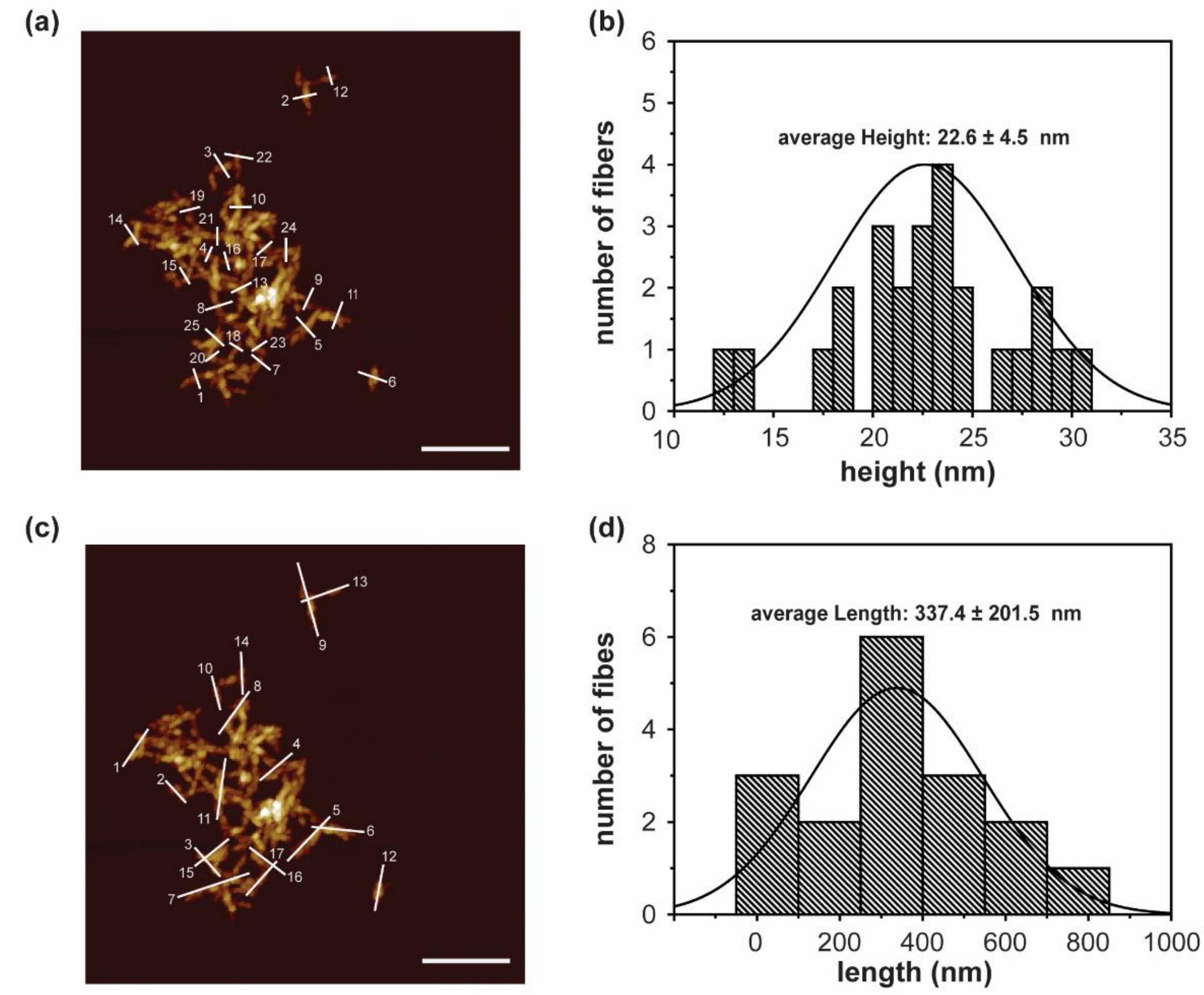

Figure S6. AFM images analysis of **Agg C** (20 µM, 1/9 DCE/MCH) spin-coated on mica (50 µL at 3000 rpm, 60 s). The numbered points of a) height and c) length of the supramolecular polymers and, corresponding b) height profiles and d) length of the fibers (scale bar: 1 µm).

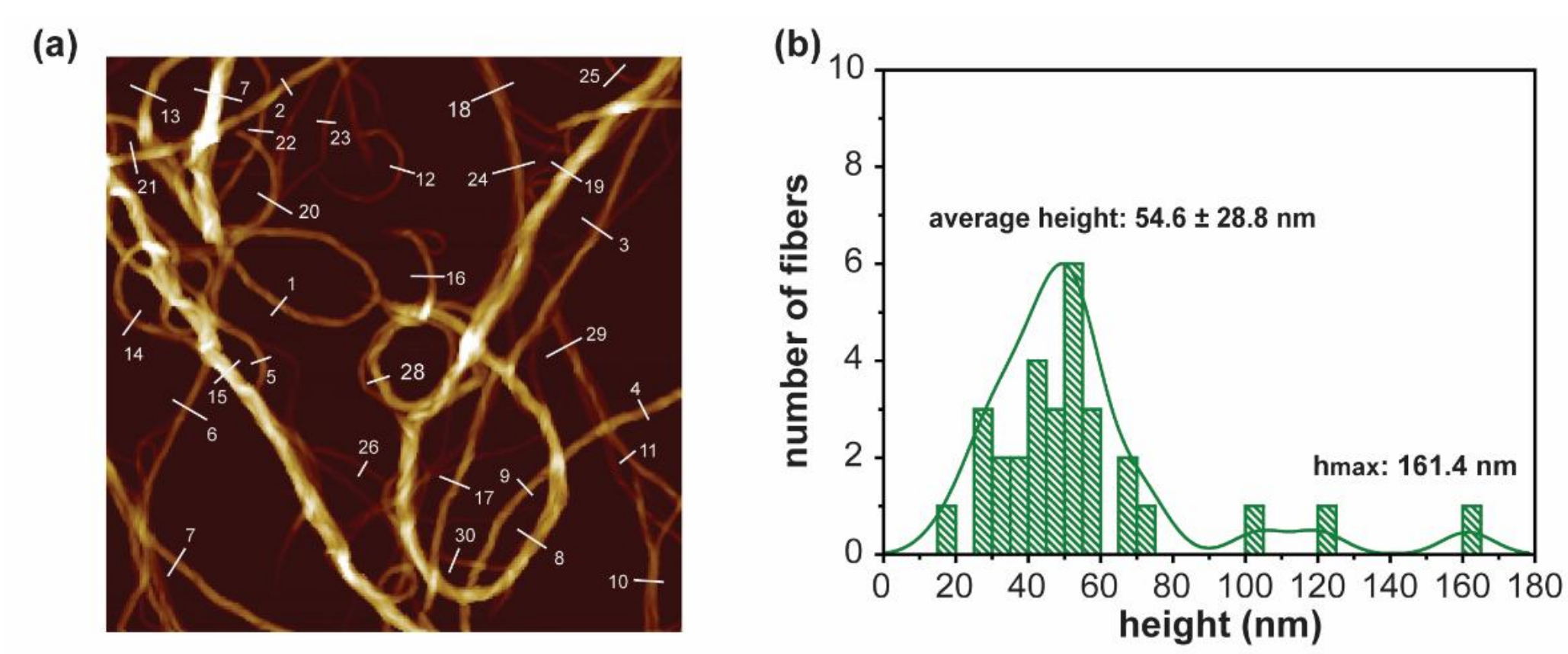

Figure S7. AFM images analysis of **Agg D** (20 µM, 1/9 DCE/MCH) spin-coated on mica (50 µL at 3000 rpm, 60 s). The numbered points of a) height of the supramolecular polymers and b) corresponding height profiles of the fibers (scale bar: 1 µm).

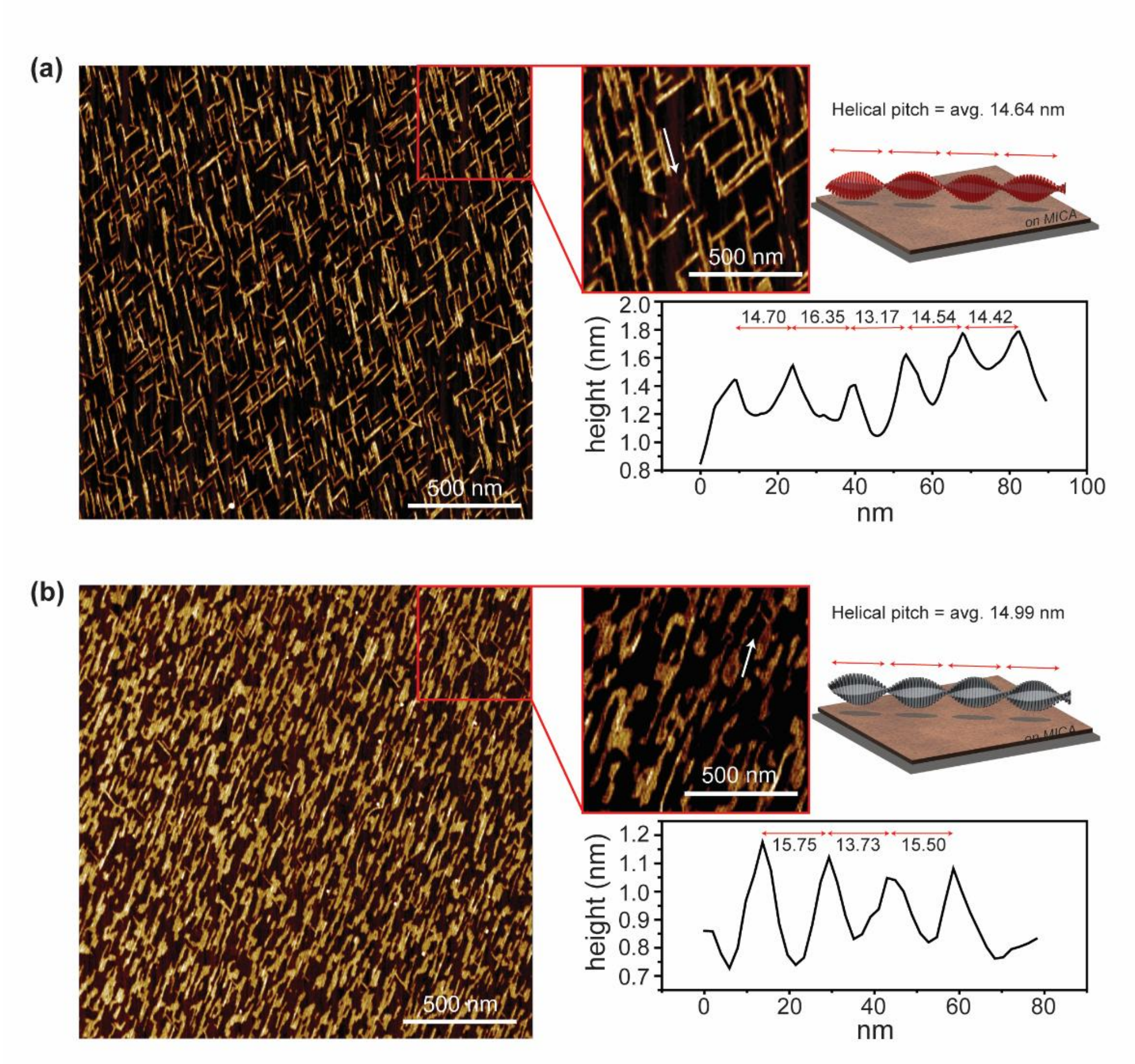

Figure S8. AFM images and helical pitch analysis of a) **Agg B** and b) **Agg C** on samples deposited on mica substrates by spin coating 10 μM 1/9 DCE/MCH solutions at 1000 rpm for 1 minute.

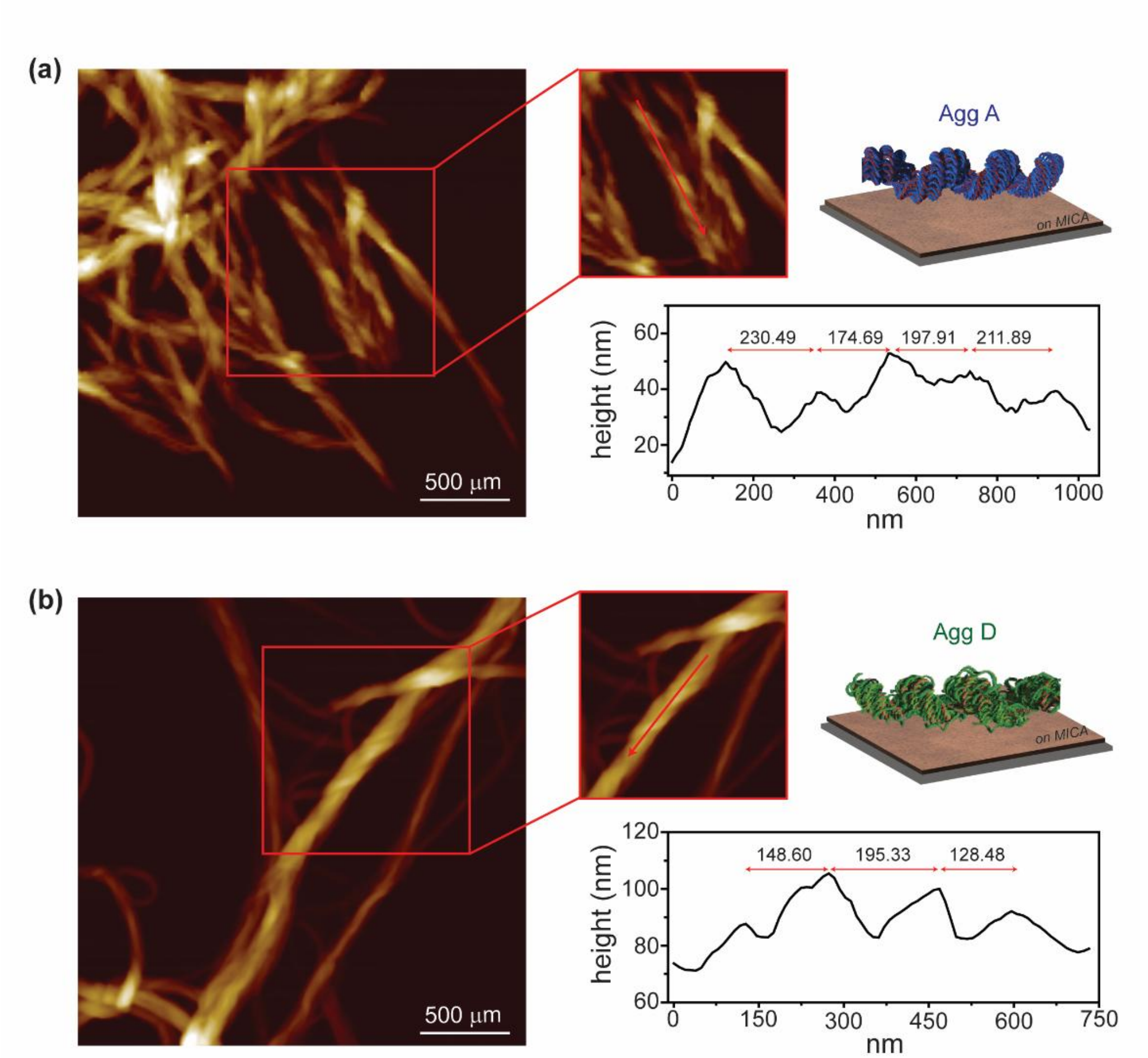

Figure S9. AFM images and helical pitch analysis of a) **Agg A** and b) **Agg D** on samples deposited on mica substrates by spin coating 20 μM 1/9 DCE/MCH solutions at 1000 rpm for 1 minute.

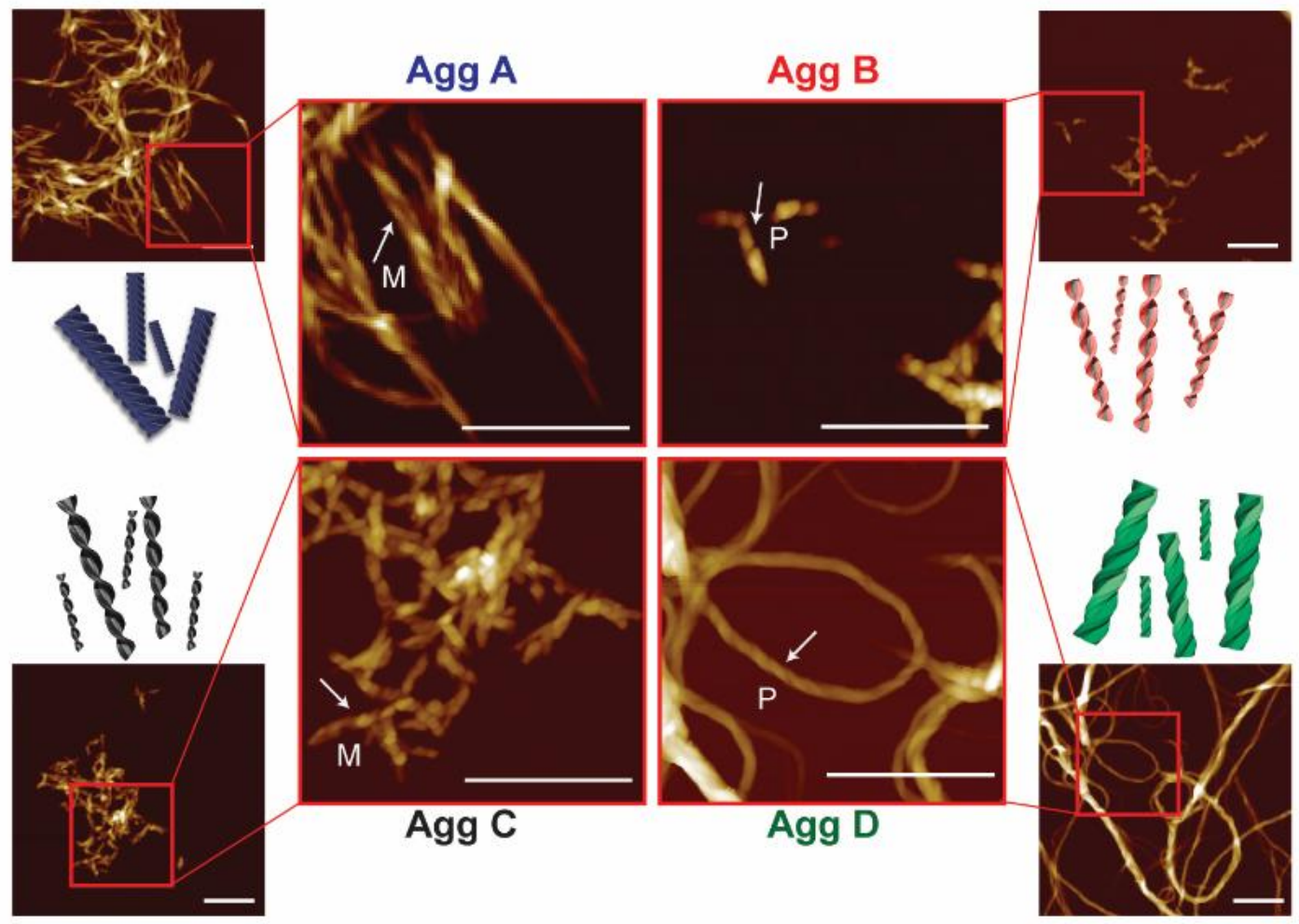

Figure S10. AFM image of **Agg A-D** (20 μM, 1/9 DCE/MCH) on mica with enlarged views highlighting the corresponding helicity of each of the helical supramolecular structures (scale bar: 1 μm).

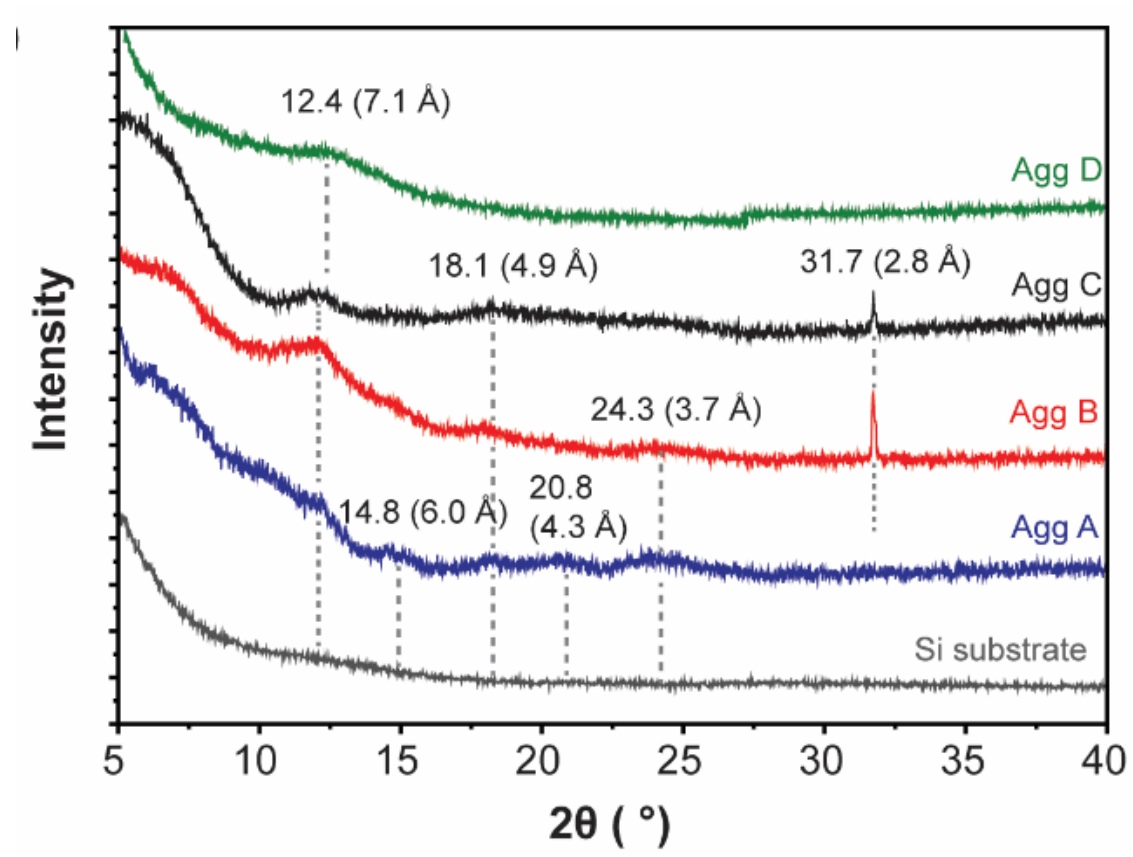

Figure S11. PXRD measurements of **Agg A-D** (20 μM, 1/9 DCE/MCH) drop-cast on zero background Si-holder.

5. ITC EXPERIMENTS DESCRIPTION

1. General experimental conditions

The ITC sample cell was filled with the DCE/MCH mixture, and 283 μL of each **Agg A-D** solution were added with a syringe. Heats of dilution were obtained by titrating each **Agg A-D** into the ITC sample cell and were recorded using a MicroCal VP-ITC (spacing time: 420 sec, temperature: 25 ± 1 °C, injection volume: 20 μL). Thermodynamic parameters were determined using OriginPro 2015 software by integrating peaks 3 through 8.

2. Control experiments

1) **Solvent-solvent titration (same vial).** The ITC cell and syringe were filled with the identical 1:9 DCE/MCH solution (prepared gravimetrically). The titration of the solvent into itself produced no detectable heat signal, confirming negligible intrinsic heat of dilution.
2) **Solvent-solvent titration (independent preparations).** Two separate 1:9 DCE/MCH solutions were independently prepared by weighing (1250 mg DCE and 6930 mg MCH for the 10% v/v mixture). One solution was placed in the cell and the other in the syringe. No significant heat signal was observed. These experiments were repeated twice, confirming that independent preparation of the solvent mixture does not introduce measurable calorimetric artifacts.

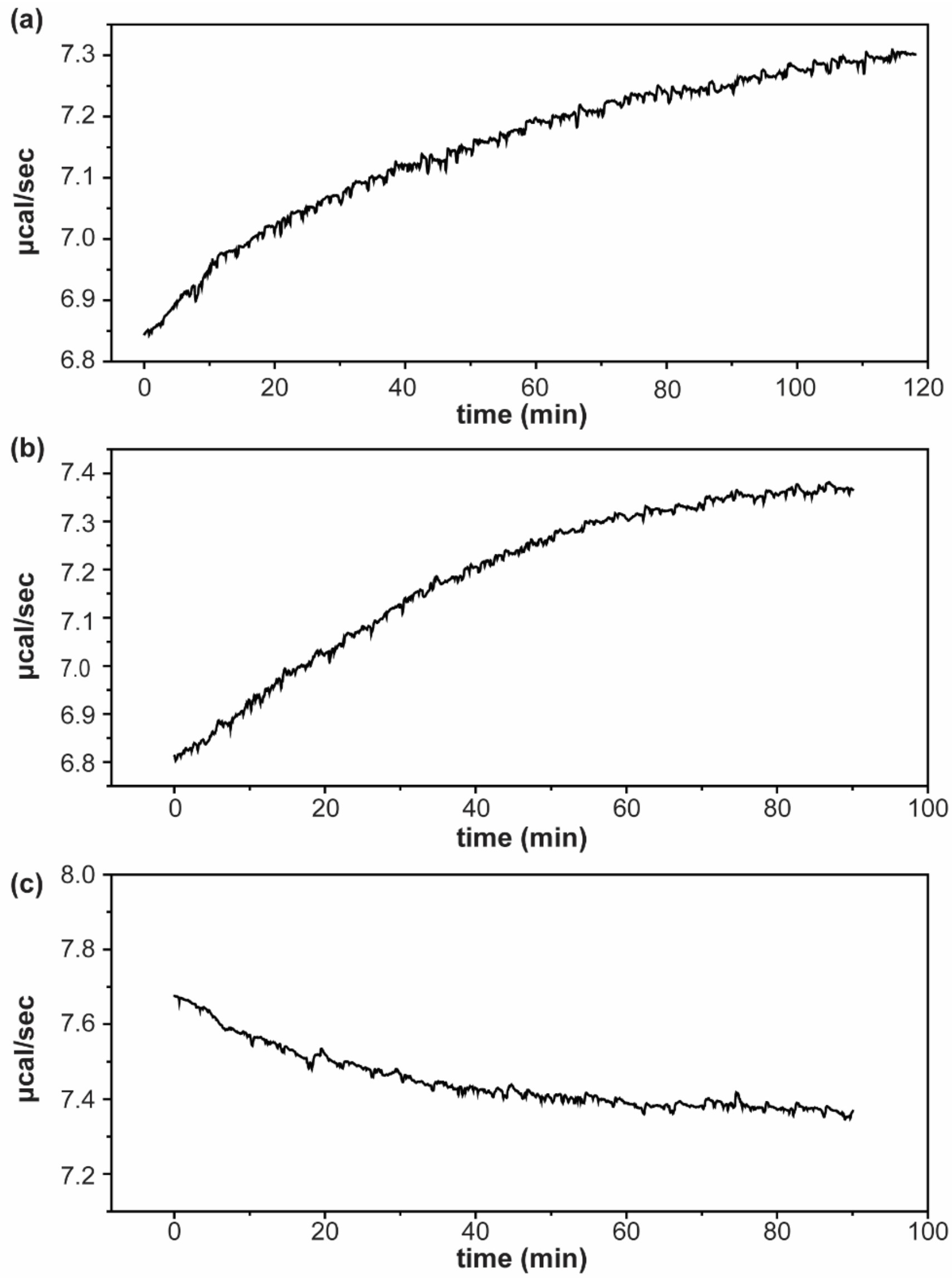

Figure S12. Solvent (10% DCE/MCH; sample cell)-solvent (10% DCE/MCH; syringe) titration a) from same vial, b) and c) separately prepared from different vials.

3) **Effect of heating and sonication on solvent composition**. Because aggregate preparation involves heating and/or sonication (see Section 2 of the SI), we verified that these treatments do not alter solvent composition. A 1:9 DCE/MCH mixture was subjected to the same thermal and sonication procedures used for aggregate preparation and then tested by solvent–solvent ITC titration. No significant heat effects were detected, demonstrating that these treatments do not measurably change the solvent composition

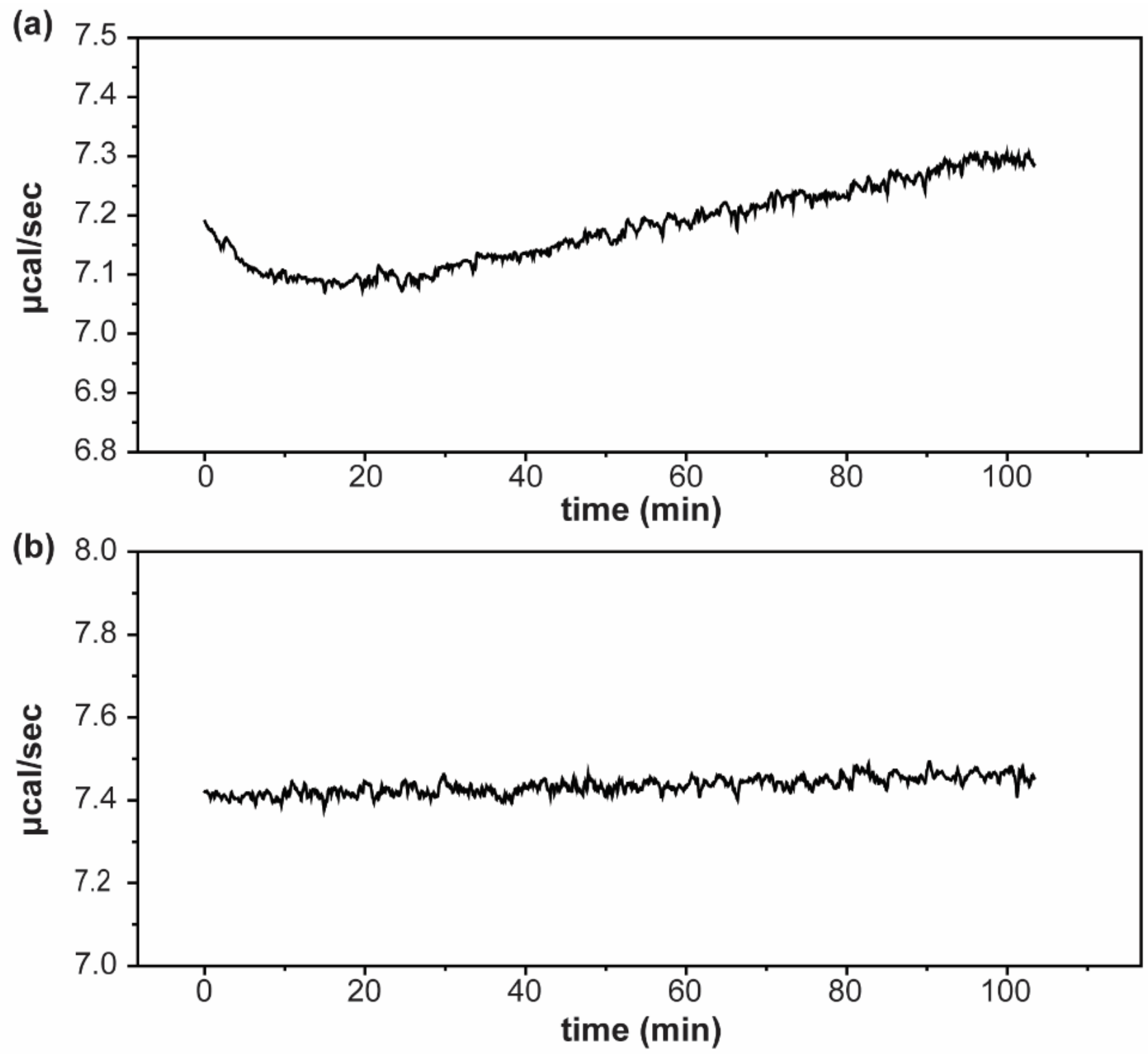

Figure S13. a) Solvent-solvent titration before applying external stimuli, and b) Solvent-solvent titration after applying external stimuli.

4) **Deliberate solvent mismatch experiments.** To evaluate the sensitivity of the system to composition deviations, we intentionally introduced small differences in DCE content (9.95%, 10.05%, 10.1%) between syringe and cell solutions (Table S1). In these cases, clear exothermic heat signals were observed, and their magnitude increased with increasing composition mismatch These control experiments demonstrate that the ITC setup is indeed sensitive to small solvent. composition differences. Importantly, the absence of such signals in our standard experiments confirms that solvent mismatch does not contribute to the measured aggregate dilution enthalpies.

Table S1. Solvent amounts at different contents of DCE.

	10 mL		Weight (mg)	
%DCE/MCH	DCE	MCH	DCE	MCH
10.1	1.01	8.99	1262.5	6922.3
10.05	1.005	8.995	1256.25	6926.15
**10**	**1**	**9**	**1250.0**	**6930**
9.95	0.995	9.005	1243.75	6933.85

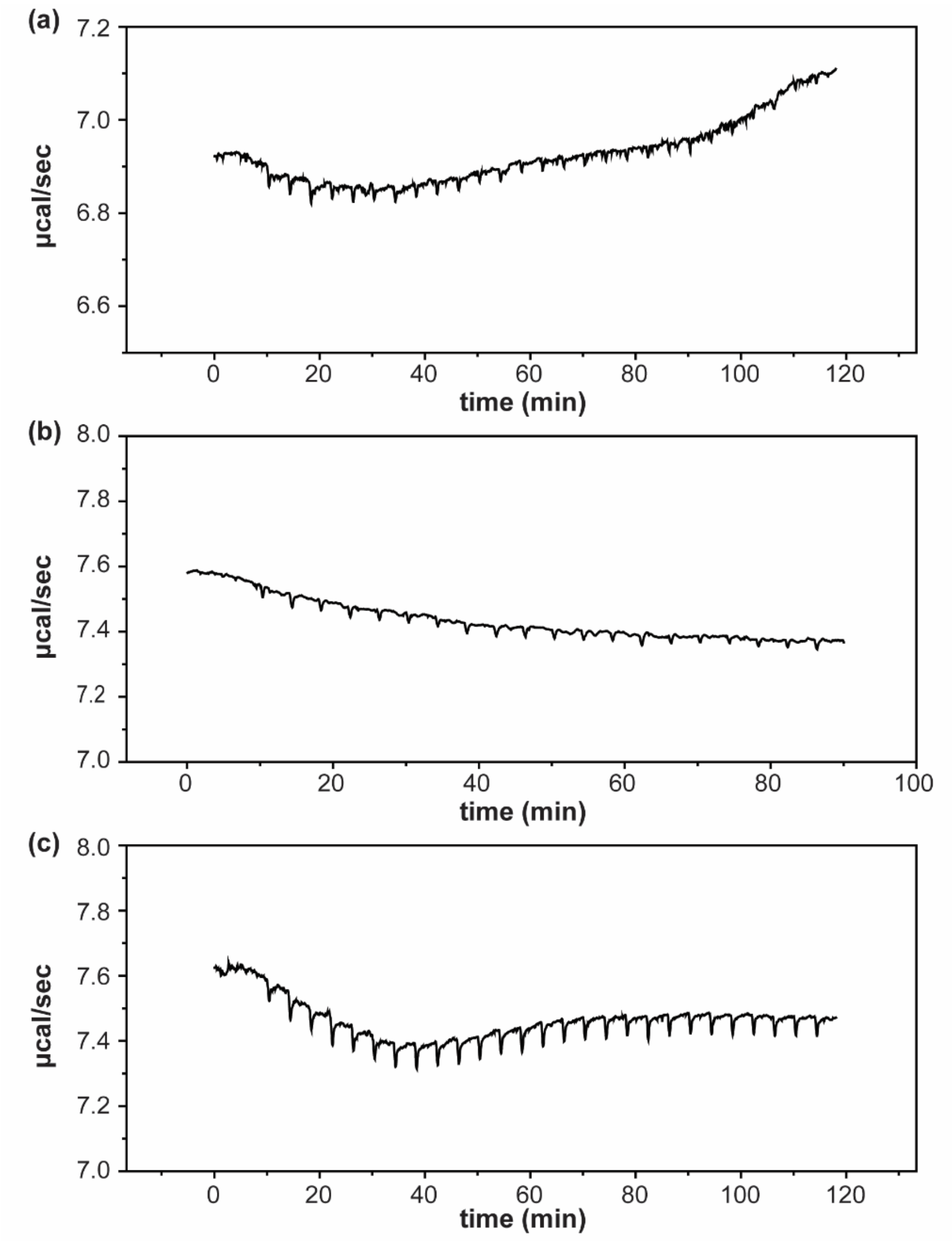

Figure S14. Solvent-solvent titration with different ratio of DCE/MCH; a)9.95% DCE/MCH, b)10.05% DCE/MCH and c) 10.1% DCE/MCH

Finally, we note that the aggregate dilution experiments (Figures S10–S14) were performed under identical solvent conditions in the syringe and cell, and that the measured endothermic signals are consistent with full disassembly upon dilution (as independently verified by CD spectroscopy in the main text). The magnitude and reproducibility of the signals across three independently prepared samples further confirm their reliability.

In summary, (i) all triplicates were performed from independently prepared solutions, and (ii) multiple dedicated control experiments—including solvent–solvent titrations, independent

gravimetric preparations, external stimulus controls, and deliberate mismatch tests, demonstrate that solvent composition artifacts do not affect the reported ITC results.

2. Sample Preparation

DCE and MCH were degassed for 20 min using a sonicator. A stock solution of **QRD** (200 μM) was prepared in degassed DCE. To prepare the solvent mixture for the sample cell, 1250 mg of DCE and 6930 mg of MCH were weighed into a 60 mL glass vial to obtain the desired 1:9 DCE/MCH volume ratio.

For the preparation of **Agg A-D** aggregates, the **QRD** stock solution was mixed with MCH by weighing the corresponding amount to achieve the desired concentrations. Each resulting solution was then used to generate **Agg A-D** according to the specific protocols

Table S2. Summary of ITC results for **Agg A-D** titrated into 1:9 DCE/MCH.

Aggregate	ΔH_{diss}(kJ/mol)	Average ΔH_{diss}(kJ/mol)
**Agg A**	137.72<br>143.64<br>138.56	139.9 ± 3.2
**Agg B**	158.06<br>161.08<br>162.41	159.1 ± 1.7
**Agg C**	173.78<br>175.60<br>180.6	176.7 ± 3.5
**Agg D**	208.42<br>220.53<br>234.90	221.2 ± 13.2

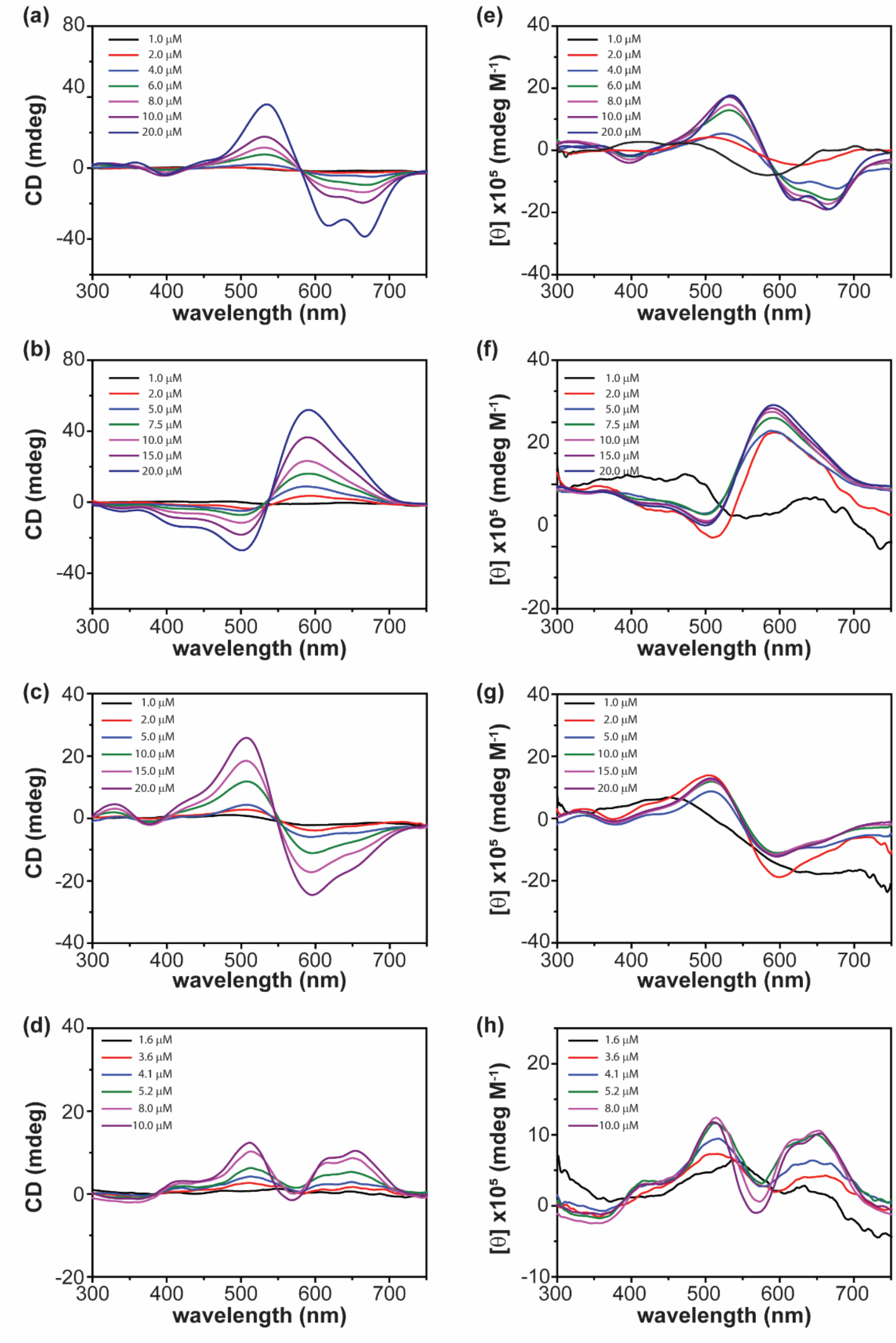

Figure S15. Concentration dependent CD spectra in 1/9 DCE/MCH (l = 1 cm, 20 °C; a and e) **Agg A**, b and f) **Agg B**, c and g) **Agg C**, and d and h) **Agg D** plotted with mdeg and molar ellipticity of CD intensity, respectively.

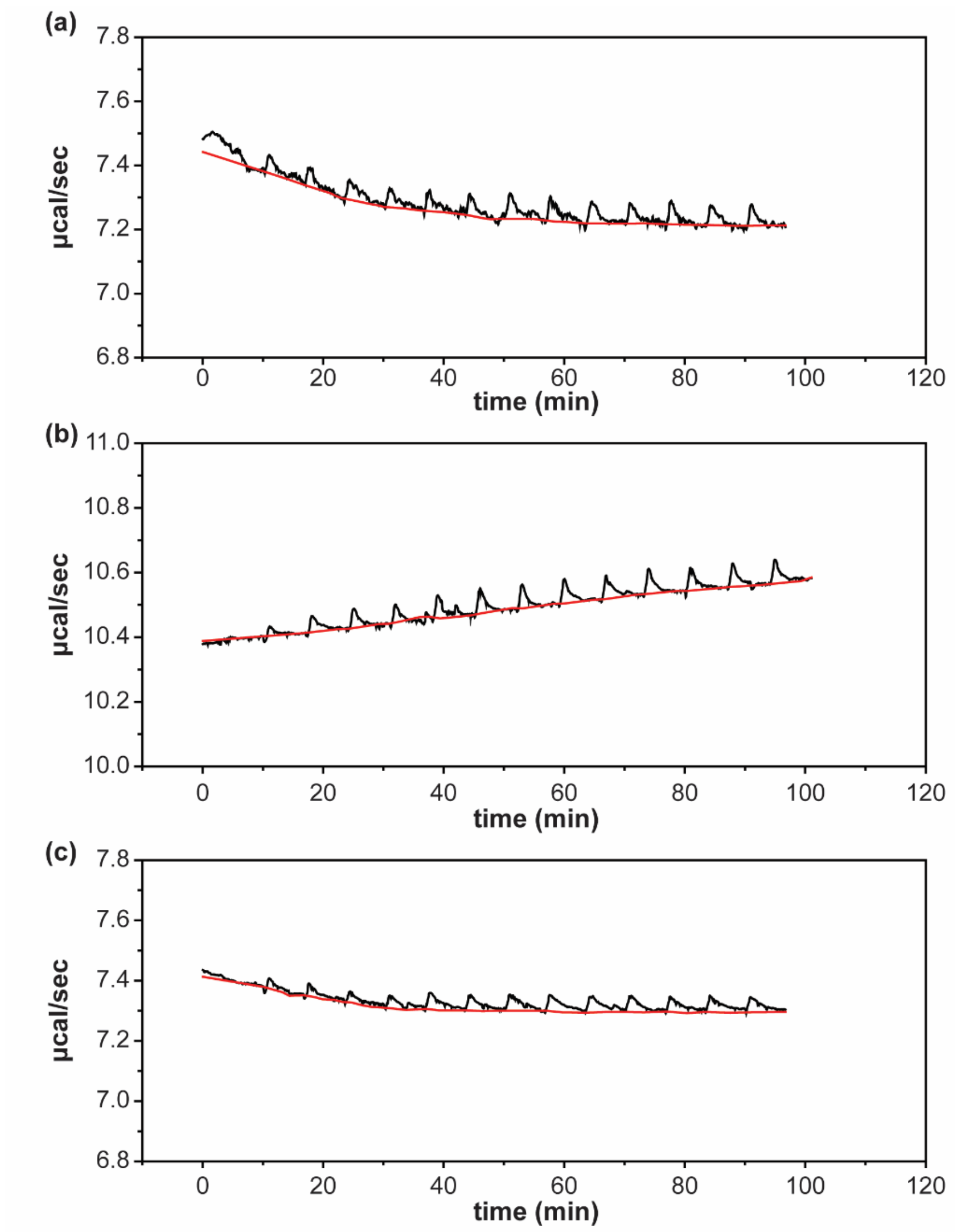

Figure S16. a)-c) ITC dilution experiments of **Agg A** (10 µM) in 1/9 DCE/MCH at 25 °C performed in triplicate.

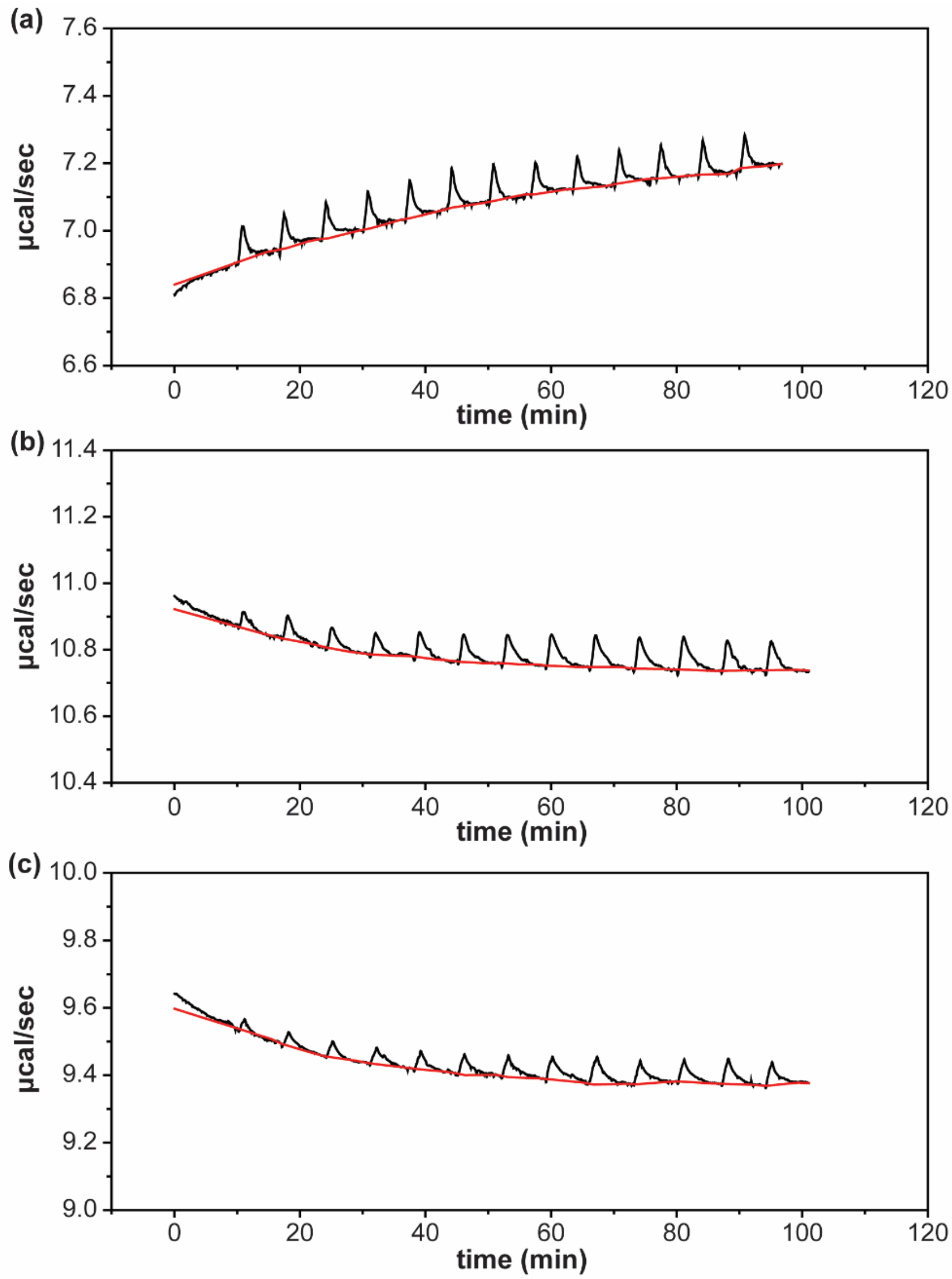

Figure S17. a)-c) ITC dilution experiments of **Agg B** (10 µM) in 1/9 DCE/MCH at 25 °C performed in triplicate.

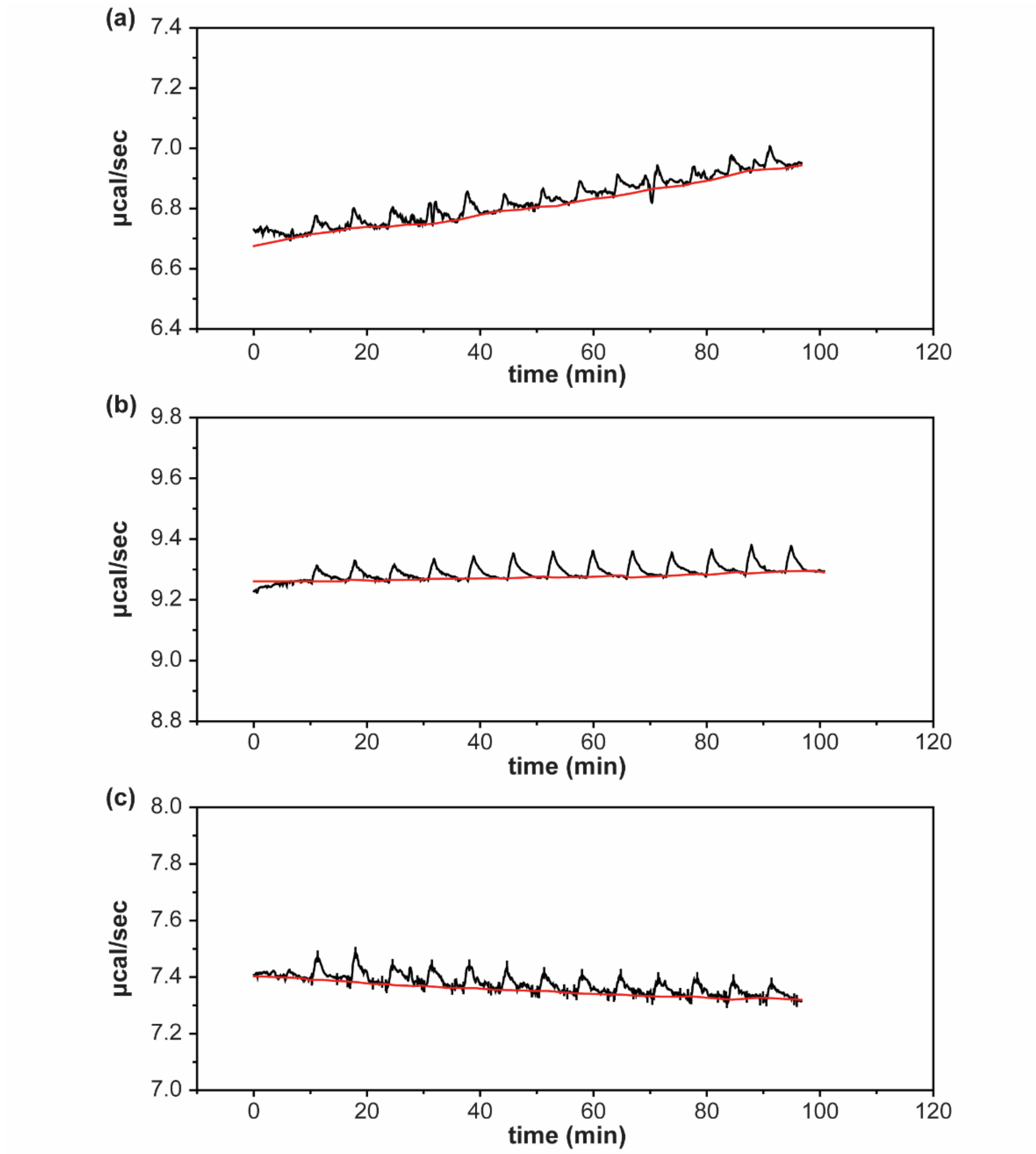

Figure S18. a)-c) ITC dilution experiments of **Agg C** (10 µM) in 1/9 DCE/MCH at 25 °C performed in triplicate.

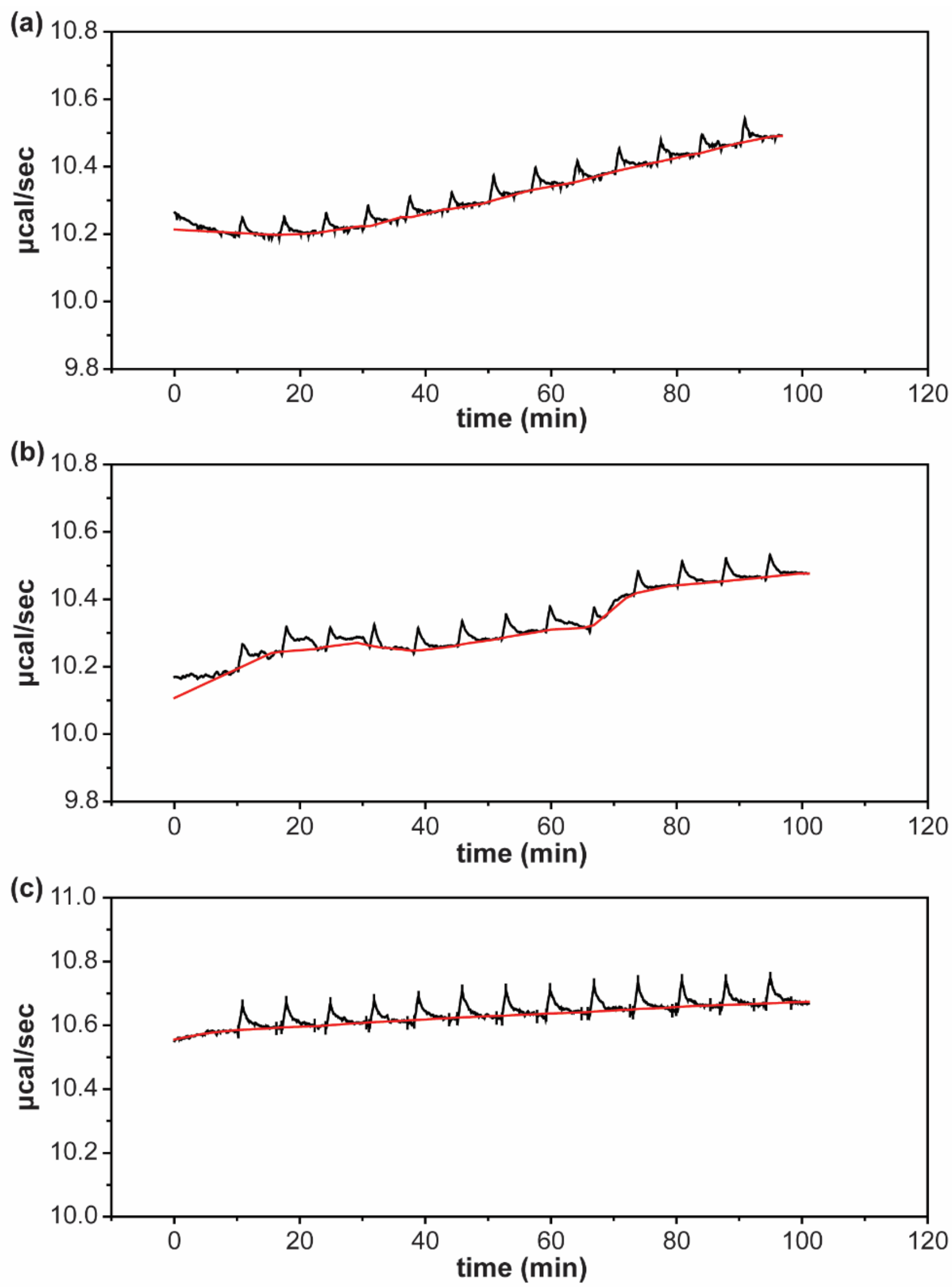

Figure S19. a)-c) ITC dilution experiments of **Agg D** (5 µM) in 1/9 DCE/MCH at 25 °C performed in triplicate.

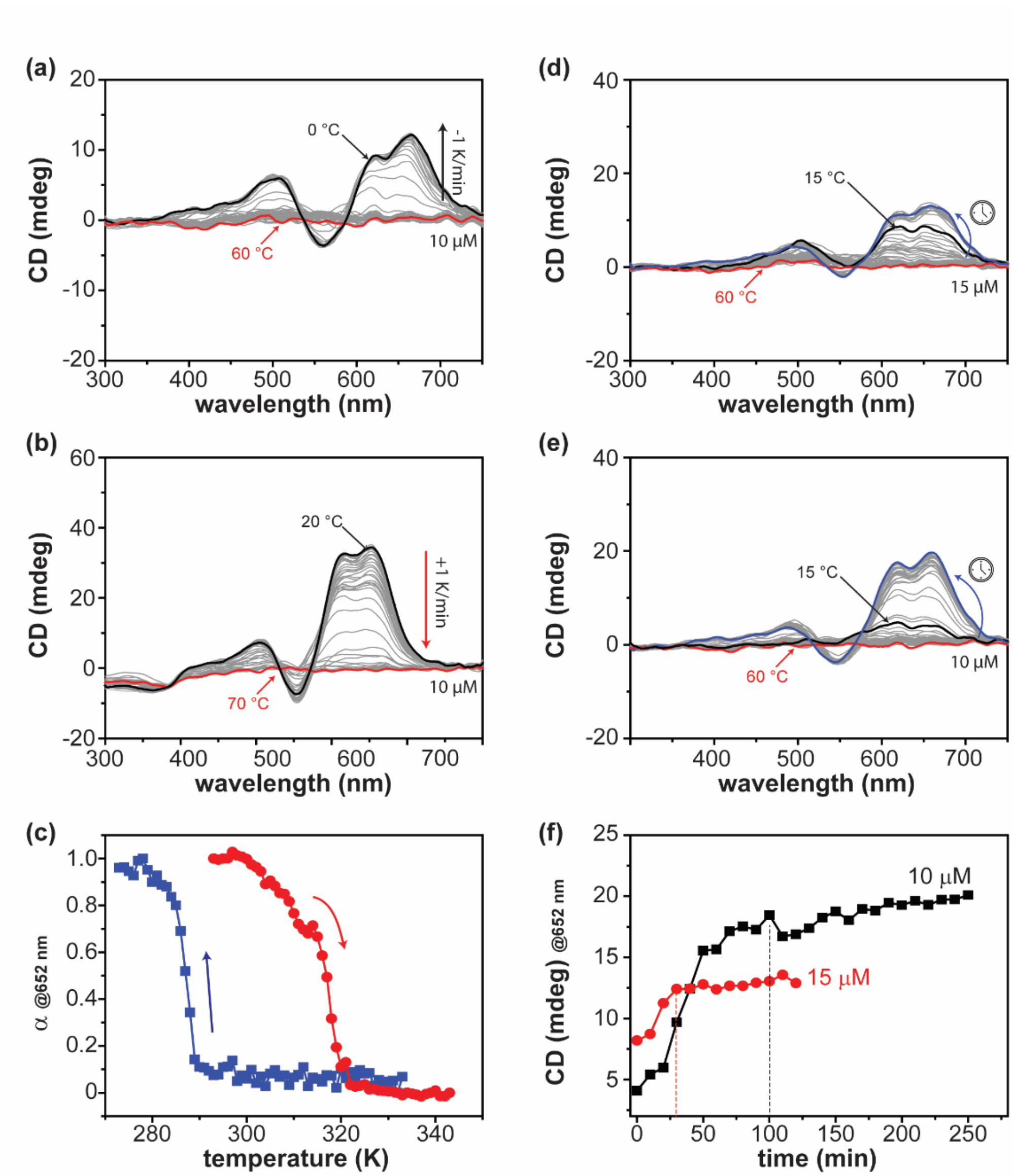

Figure S20. Variable temperature CD spectra of **QRD** (10 µM in 10% DCE/MCH) a) cooling from 60 °C to 0 °C (1 K/min), b) heating from 20 °C to 70 °C (1 K/min), and c) degree of aggregation of heating and cooling process extracted from (a) and (b) monitored at 652 nm. Variable temperature CD spectra of **QRD** cooling from 60 °C to 15 °C (1 K/min) and time dependent kinetic experiment to grow **Agg D** at 15 °C at different concentrations; d) 15 µM and e) 10 µM, (f) CD intensity monitored at 652 nm over time extracted from graph (d) and (e).

6. Description of spectral deconvolution

6.1 Deconvolution method for kinetic experiment of Agg A at different concentrations.

To quantitatively estimate the relative contributions of different aggregate species to the experimentally observed circular dichroism (CD) spectra, a deconvolution analysis was performed using four reference spectra corresponding to **Agg A**, **Agg B**, **Agg C**, and **Agg D** states.

Each reference spectrum was first normalized with respect to its maximum ellipticity and used as a basis function for linear combination fitting. The experimental CD spectrum was expressed as:

(1) $CD_{calc}(\lambda) = a \times CD_{\mathbf{Agg\ A}}(\lambda) + b \times CD_{\mathbf{Agg\ B}}(\lambda) + c \times CD_{\mathbf{Agg\ C}}(\lambda) + d \times CD_{\mathbf{Agg\ D}}(\lambda)$

where a, b, c and d denote the fractional contributions of each aggregate species. The coefficients (a, b, c, d) were optimized by minimizing the sum of squared residuals between the experimental and calculated spectra:

(2) Minimize $\Sigma\lambda\ [CD\_exp(\lambda) - CD\_calc(\lambda)]^2$

The fitting was carried out in Microsoft Excel (Solver add-in) using the file “CD_deconvolution”. The 'Weight' and 'Fraction' rows represent the optimized coefficients and their normalized values, respectively. The 'SUM' column corresponds to the reconstructed spectrum obtained from the linear combination, and the 'errors' column contains the squared residuals for each wavelength.

Solver parameters: Objective – minimization of the total squared error; Adjustable variables – weights (a, b, c, d); Constraints – a, b, c, $d \geq 0$ and $a + b + c + d = 1$.

The experimental CD spectra used for this analysis are provided in Sheet4 of the same Excel file. The deconvolution results demonstrated excellent agreement between the reconstructed and experimental spectra ($R^2 > 0.99$), validating the reliability of the approach.

Example of CD spectral deconvolution was presented in the “CD_deconvolution” file (available on https://github.com/hermanslab/4ChiralAgg). Experimental spectrum (black solid line) and reconstructed spectrum (red dashed line) obtained from the linear combination of reference spectra corresponding to **Agg A**, **Agg B**, **Agg C**, and **Agg D**.

Table S3. Fractional contributions (*a*, *b*, *c*, *d*) of **Agg A**, **Agg B**, **Agg C**, and **Agg D** species obtained from CD spectral deconvolution using **eq. S1** for kinetic experiments on **Agg A**.

**Concentration (μM)**	**Time (d)**	**Agg A (a)**	**Agg B (b)**	**Agg C (c)**	**Agg D (d)**
5	0	1.00	0.00	0.00	0.00
	9	0.36	0.28	0.36	0.00
	16	0.31	0.30	0.39	0.00
	26	0	0.52	0.46	0.02
10	0	1.00	0.00	0.00	0.00
	9	0.77	0.00	0.23	0.00
	16	0.38	0.36	0.15	0.11
	26	0.33	0.30	0.00	0.37
20	0	1.00	0.00	0.00	0.00
	9	0.73	0.17	0.00	0.10
	16	0.53	0.36	0.00	0.11
	26	0.36	0.36	0.00	0.29

6.2 Deconvolution method for kinetic experiment of Agg B and Agg C in the absence and presence of seed

To quantitatively estimate the relative contributions of different aggregate species to the experimentally observed circular dichroism (CD) spectra, a deconvolution analysis was performed using three reference spectra corresponding to **Agg B**, **Agg C**, and **Agg D** states.

Each reference spectrum was first normalized with respect to its maximum ellipticity and used as a basis function for linear combination fitting. The experimental CD spectrum was expressed as:

(3) $CD_{calc}(\lambda) = b \times CD_{\mathbf{Agg\ B}}(\lambda) + c \times CD_{\mathbf{Agg\ C}}(\lambda) + d \times CD_{\mathbf{Agg\ D}}(\lambda)$

where b, c and d denote the fractional contributions of each aggregate species. The coefficients (b, c, d) were optimized by minimizing the sum of squared residuals between the experimental and calculated spectra:

(4) Minimize $\Sigma\lambda\ [CD\_exp(\lambda) - CD\_calc(\lambda)]^2$

The fitting was carried out in Microsoft Excel (Solver add-in) using the file “CD_deconvolution”. The 'Weight' and 'Fraction' rows represent the optimized coefficients and their normalized values, respectively. The 'SUM' column corresponds to the reconstructed spectrum obtained from the linear combination, and the 'errors' column contains the squared residuals for each wavelength.

Solver parameters: Objective – minimization of the total squared error; Adjustable variables – weights (*b*, *c*, *d*); Constraints – $b, c, d \geq 0$ and $b + c + d = 1$.

The experimental CD spectra used for this analysis are provided in Sheet4 of the same Excel file. The deconvolution results demonstrated excellent agreement between the reconstructed and experimental spectra ($R^2 > 0.99$), validating the reliability of the approach.

Example of CD spectral deconvolution was presented in the “CD_deconvolution” file (available on https://github.com/hermanslab/4ChiralAgg). Experimental spectrum (black solid line) and reconstructed spectrum (red dashed line) obtained from the linear combination of reference spectra corresponding to **Agg B**, **Agg C**, and **Agg D**.

Table S4. Fractional contributions (*b*, *c*, *d*) of **Agg B**, **Agg C**, and **Agg D** species obtained from CD spectral deconvolution using **eq. S3** for (un)seeded experiments on **Agg B**.

	**Concentration (µM)**	**time (d)**	**Agg B (a)**	**Agg C (b)**	**Agg D (c)**
**Unseeded**	20	0	0.98	0.00	0.02
		3	0.95	0.04	0.01
		7	0.85	0.12	0.03
		14	0.86	0.11	0.03
	15	0	0.98	0.00	0.02
		3	0.88	0.09	0.03
		7	0.75	0.20	0.05
		14	0.85	0.14	0.02
	10	0	1.00	0.00	0.00
		3	0.89	0.11	0.00
		7	0.70	0.24	0.06
		14	0.76	0.20	0.04
	**Concentration (µM)**	**Seed C (%)**	**Agg B (a)**	**Agg C (b)**	**Agg D (c)**
**Seeded**	10	0	0.98	0.00	0.02
		0.05	0.00	0.00	1.00
		0.1	0.00	0.00	1.00
		0.5	0.00	0.01	0.99
		10	0.00	0.08	0.92

Table S5. Fractional contributions (b, c, d) of **Agg B**, **Agg C**, and **Agg D** species obtained from CD spectral deconvolution using **eq. S3** for (un)seeded experiments on **Agg C**.

	Concentration (μM)	Time (d)	Agg B (b)	Agg C (c)	Agg D (d)
**Unseeded**	30	0	0.00	0.98	0.02
		3	0.44	0.38	0.18
		7	0.46	0.28	0.27
		14	0.39	0.27	0.35
	20	0	0.00	1.00	0.00
		3	0.48	0.38	0.14
		7	0.43	0.36	0.21
		14	0.47	0.31	0.22
	15	0	0.08	0.92	0.01
		3	0.36	0.52	0.11
		7	0.49	0.33	0.18
		14	0.46	0.31	0.23
	**Concentration (μM)**	**Seed B (%)**	**Agg B (b)**	**Agg C (c)**	**Agg D (d)**
**Seeded**	10	0	0.00	0.98	0.02
		0.05	0.05	0.57	0.38
		0.1	0.24	0.54	0.22
		0.5	0.21	0.49	0.30
		10	0.00	0.49	0.51

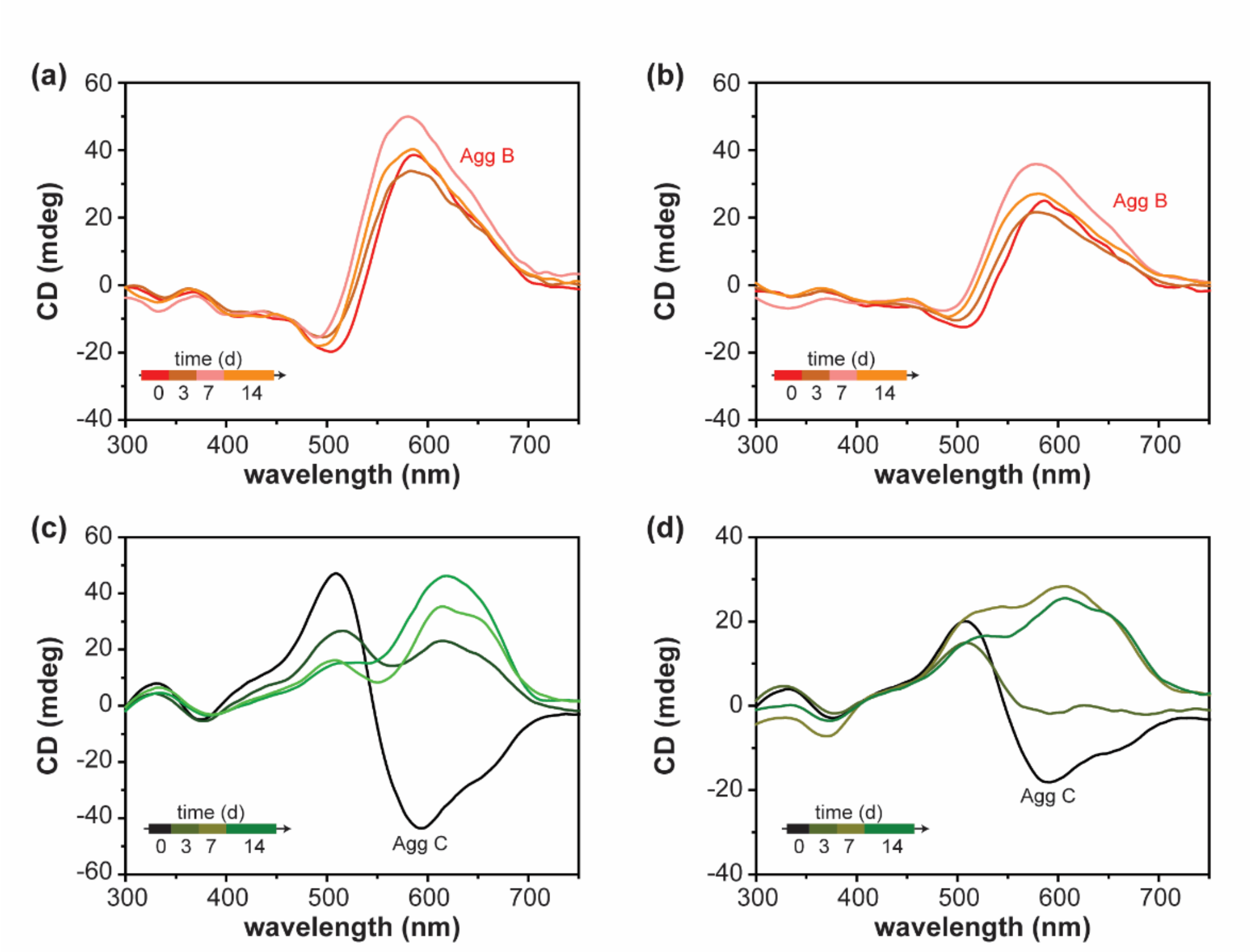

Figure S21. CD spectral changes of a) **Agg B** (15 μM, 1/9 DCE/MCH), b) **Agg B** (10 μM, 1/9 DCE/MCH), c) **Agg C** (30 μM, 1/9 DCE/MCH), and d) **Agg C** (15 μM, 1/9 DCE/MCH) over the course of 14 days at room temperature.

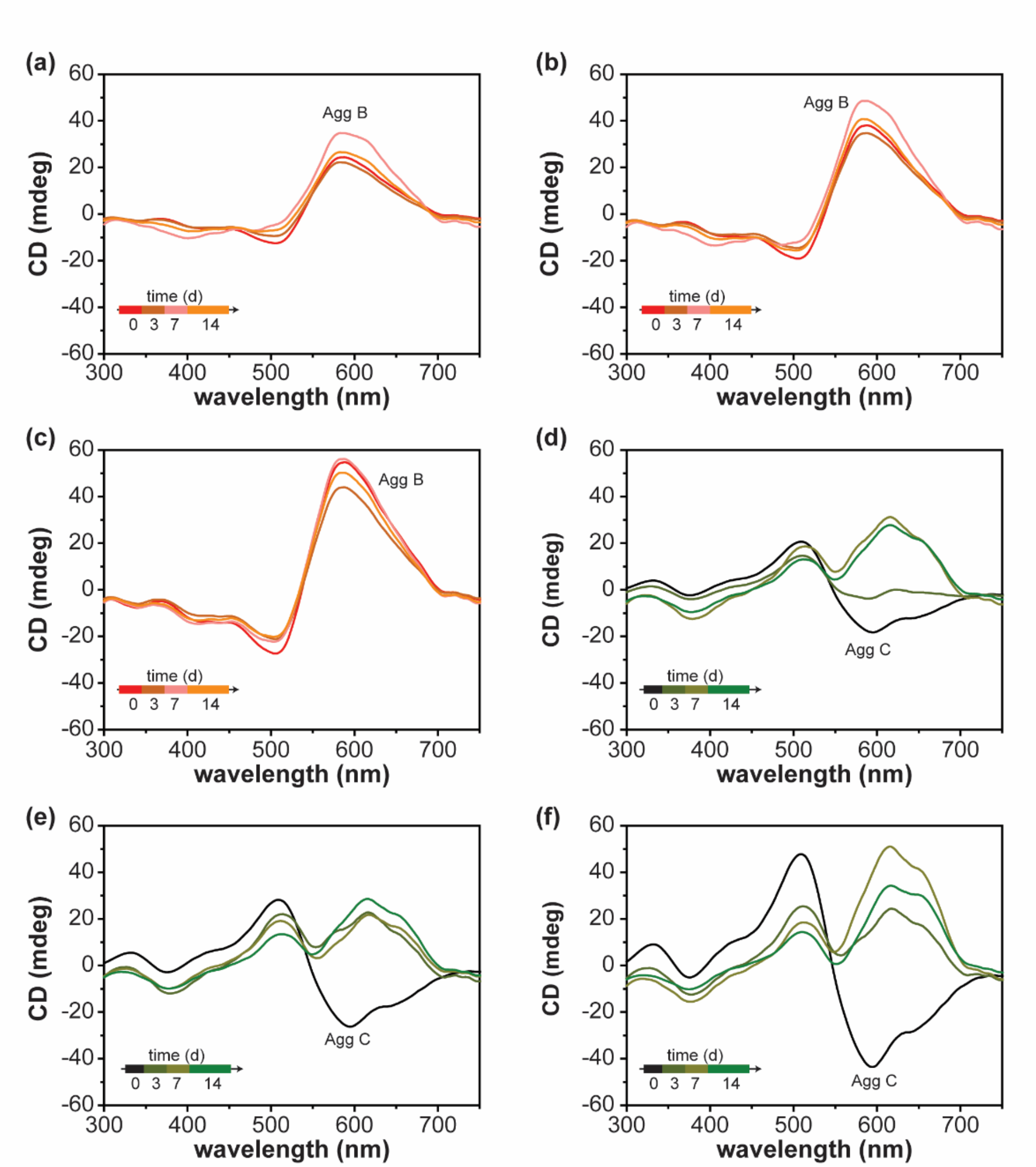

Figure S21. Reconstructed CD spectral changes of **Agg B** over the course of 14 days at room temperature at different concentrations; a) 10 μM, b) 15 μM, and c) 20 μM, and **Agg C** over the course of 14 days at room temperature at different concentrations; a) 15 μM, b) 20 μM, and c) 30 μM.

7. Seeding conversion experiment

1) General experimental conditions

To perform the seeding conversion experiments, **Agg B** and **Agg C** solutions were prepared by following the protocols described in Section 2. As summarized in table S6, **Agg B** and **Agg C** were mixed at different volumetric ratios.

It should be noted that both **Agg B** and **Agg C** solutions were prepared solely according to the standard protocols, and no additional treatment was applied to generate seeds before it was mixed.

After mixing, the circular dichroism (CD) spectra of the resulting solutions were monitored after 24 hours to evaluate the seeding induced conversion behaviors.

Table S6. Volumetric ratios of **Agg B** and **Agg C** used for the seeding conversion experiments.

**Seed**		**0.5% (v/v)**	**1%**	**2%**	**5%**	**10%**
Exp 1.	Agg C	12.5	25	50	125	250
	Agg B	2487.5	2475	2450	2375	2250
Exp 2.	Agg B	12.5	25	50	125	250
	Agg C	2487.5	2475	2450	2375	2250

Exp 1. is the seeding conversion experiment with varying amount of **Agg C**

Exp 2. Is the seeding conversion experiment with varying amount of **Agg B**.

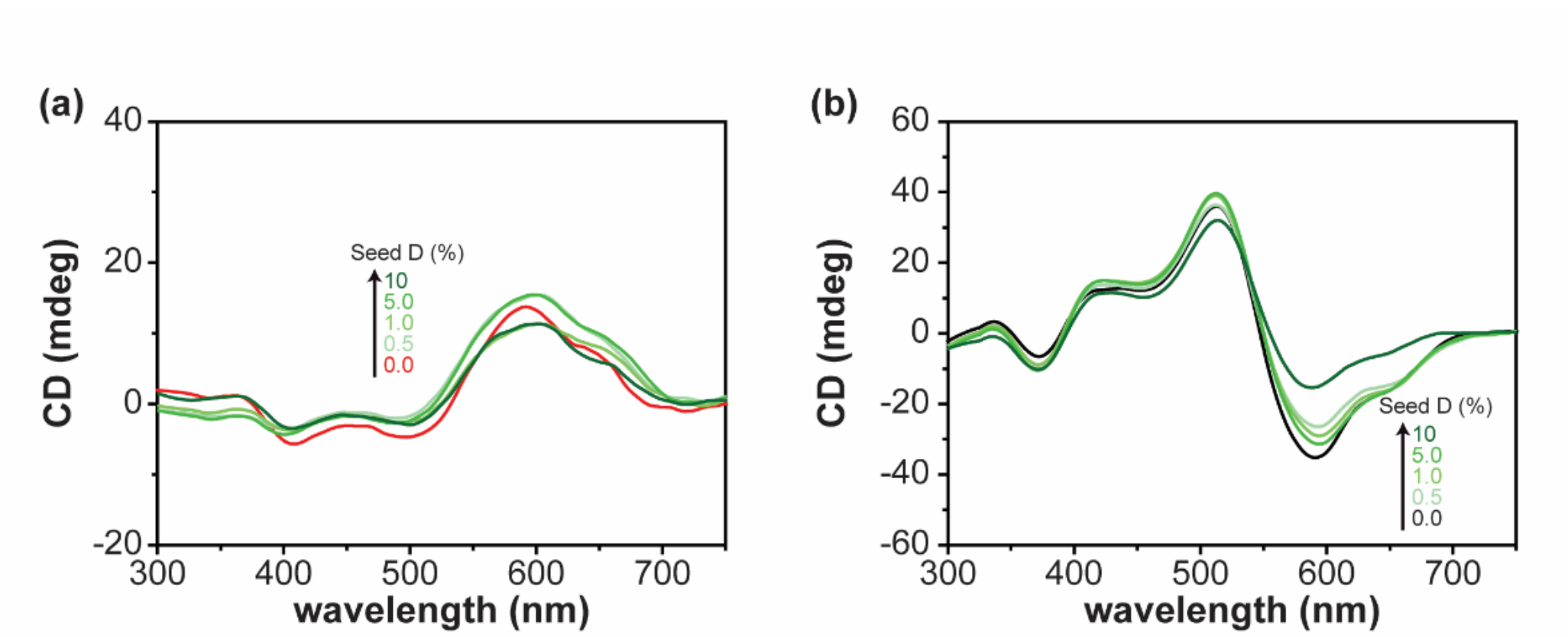

Figure S23. No observed CD spectral changes of a) **Agg B** (10 μM, 1:9 DCE/MCH) and b**) Agg C** (10 μM, 1/9 DCE/MCH) in the presence of **Agg D** seeds.

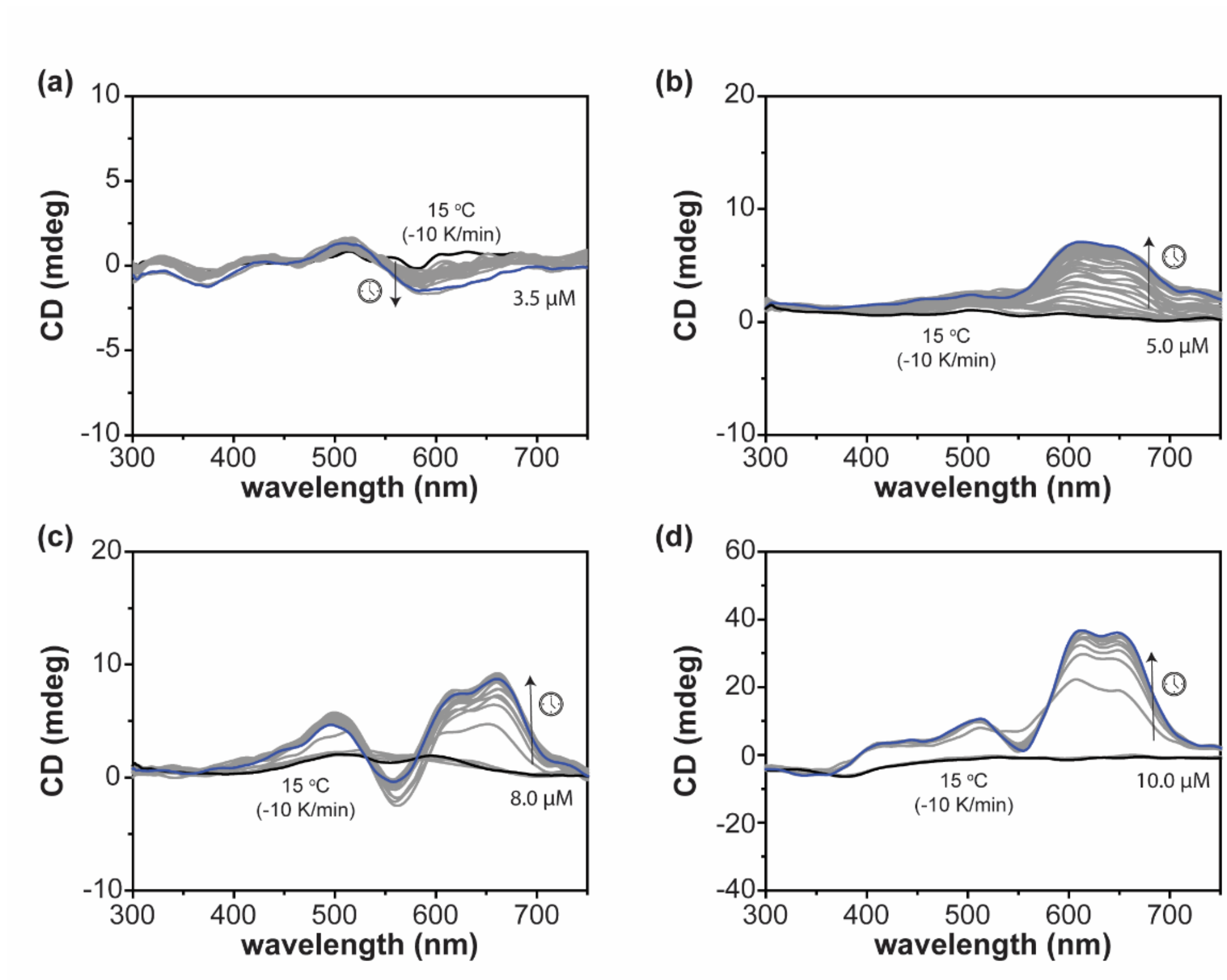

Figure S24. Time dependent CD spectral changes of **QRD** at 15 °C after cooling down from 45 °C (10 K/min) at different concentrations; a) 3.5 μM, b) 5.0 μM, c) 8.0 μM, and d) 10.0 μM recorded in 10% DCE/MCH.

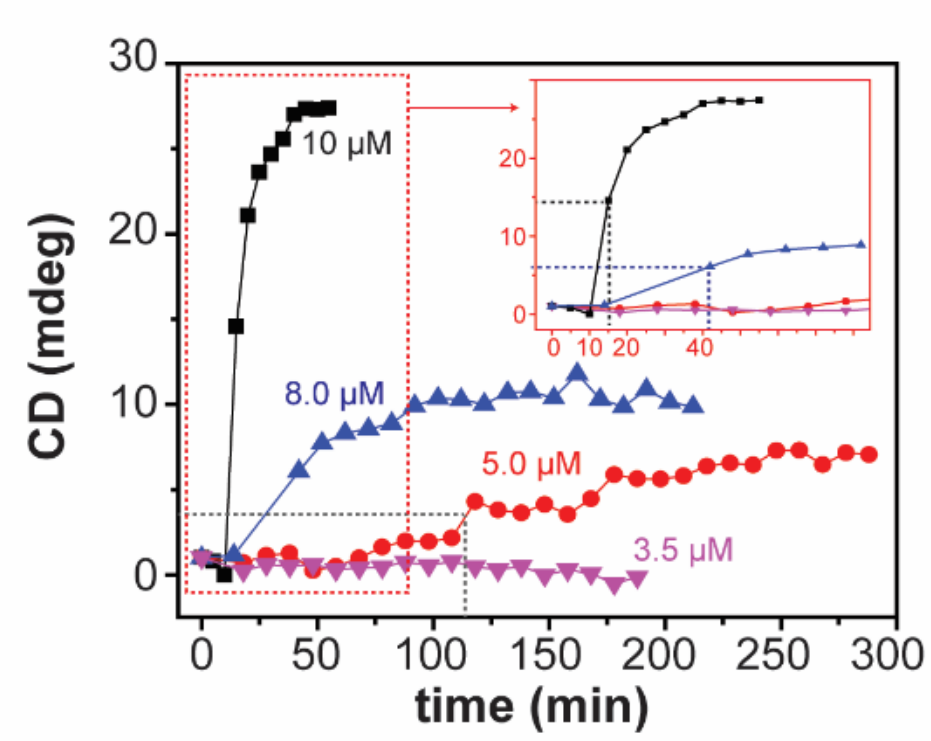

Figure S25. Time dependent CD intensity changes of **QRD** monitored at 653 nm upon cooling down to 15 °C at different concentrations.

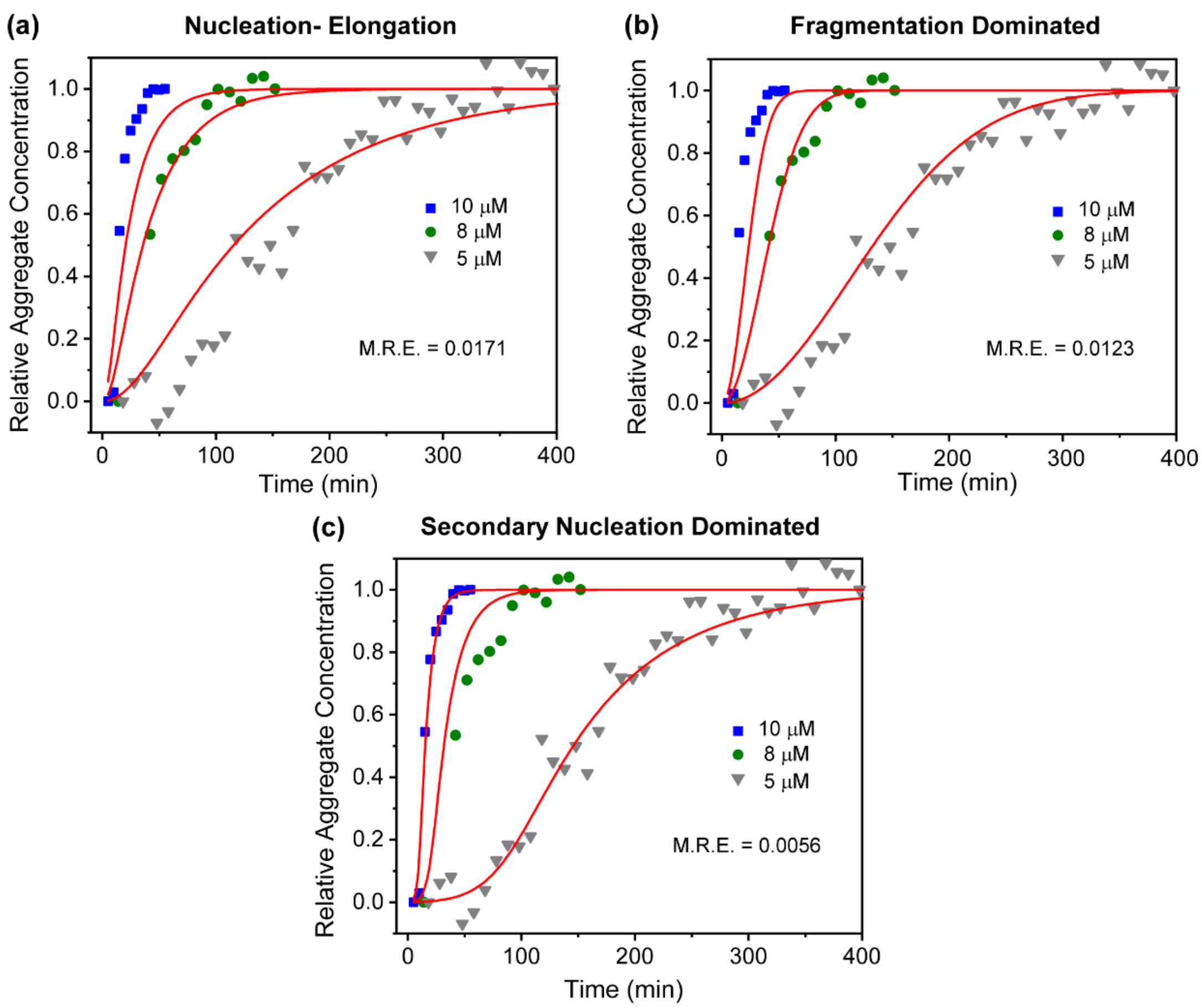

Figure S26. Kinetics of formation of **Agg D** from the monomer at different concentrations (blue squares 10 μM, green circles 8 μM and grey inverted triangles 5 μM). Fittings of the curves to the (a) nucleation-elongation model (b) Fragmentation dominated model and (c) secondary nucleation model of the Amylofit software.

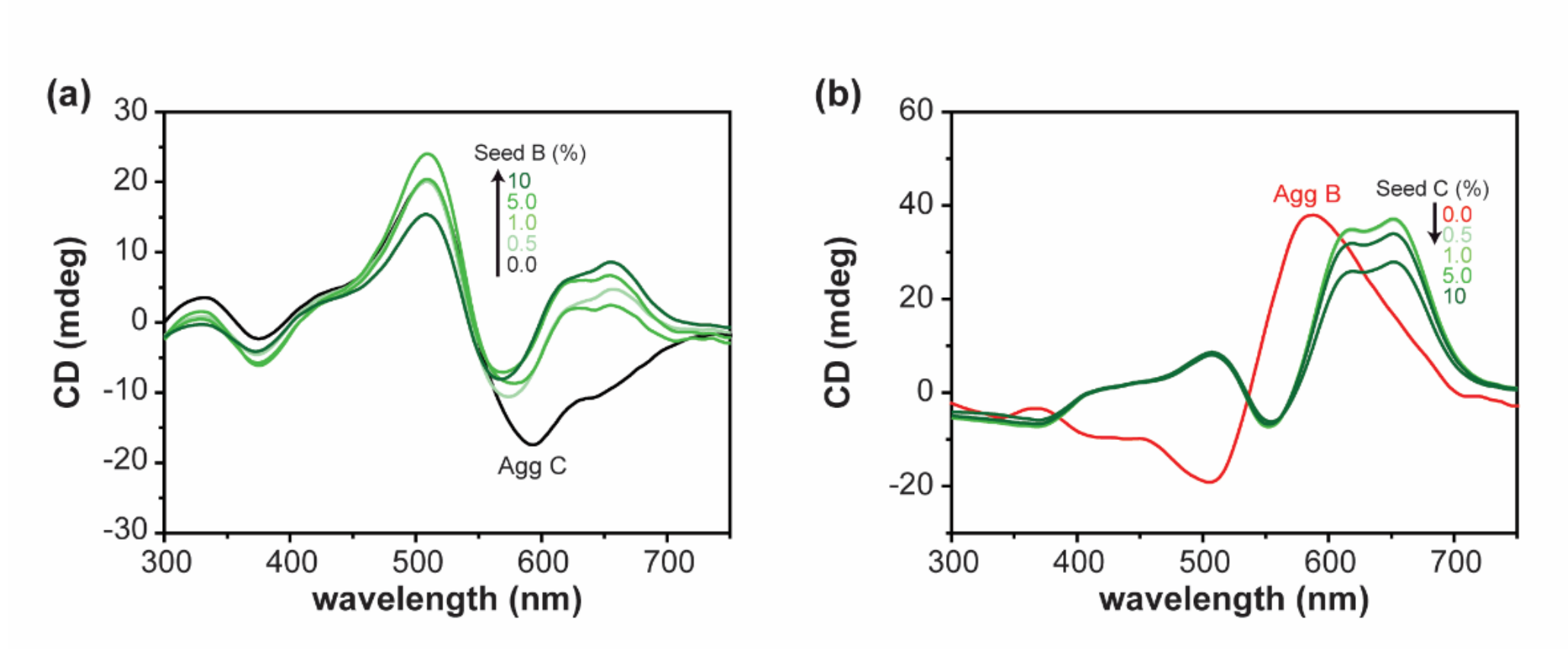

Figure S27. Reconstructed spectra of a) **Agg C** with seeds of **Agg B** and b) **Agg B** with seeds of **Agg C** obtained from deconvolution of the experimental CD spectra of cross-seeding experiments between **Agg B** and **Agg C**.

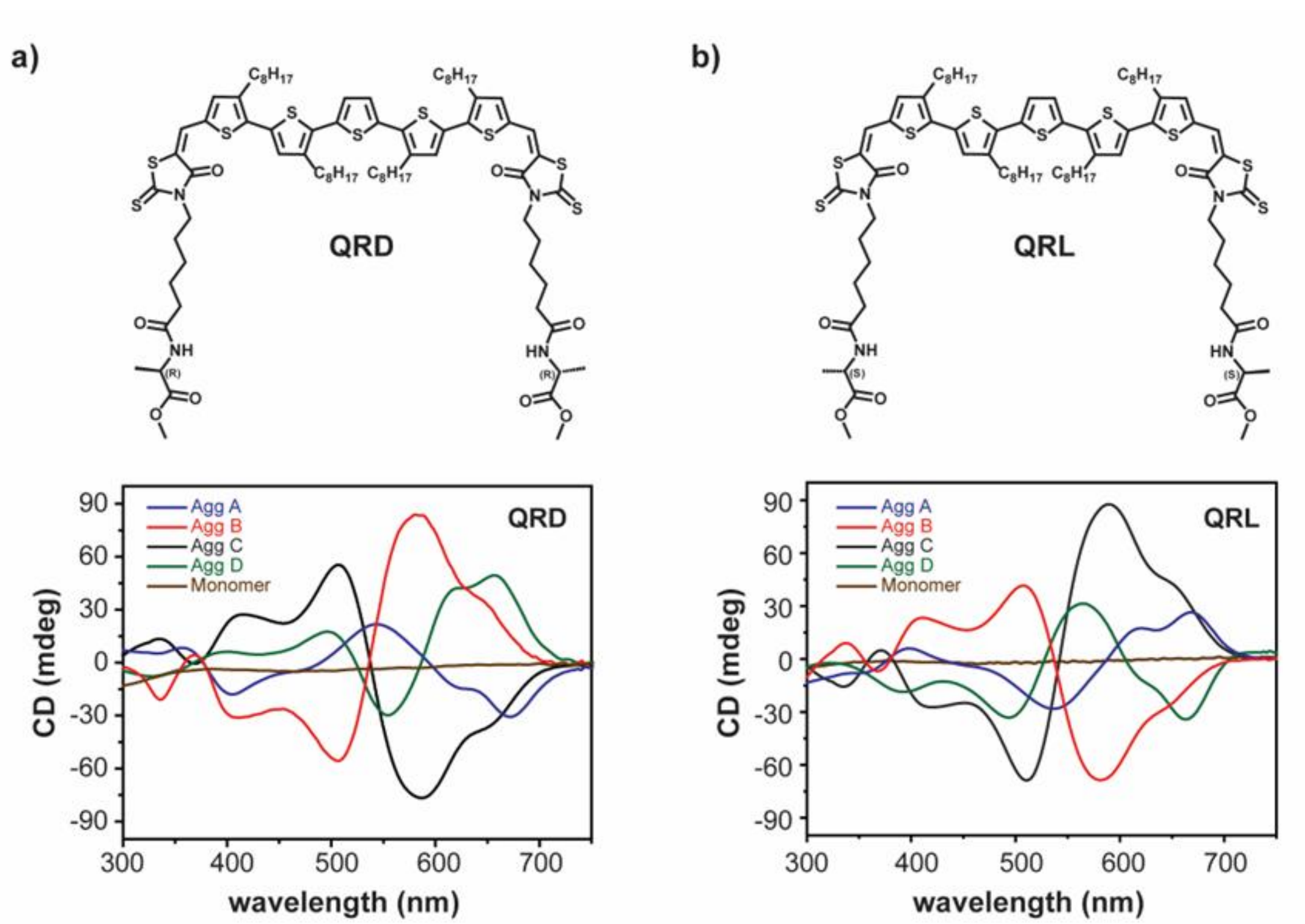

Figure S28. CD spectra of **Agg A-D** prepared following the protocols described in section 2 of a) **QRD** and b) **QRL**.

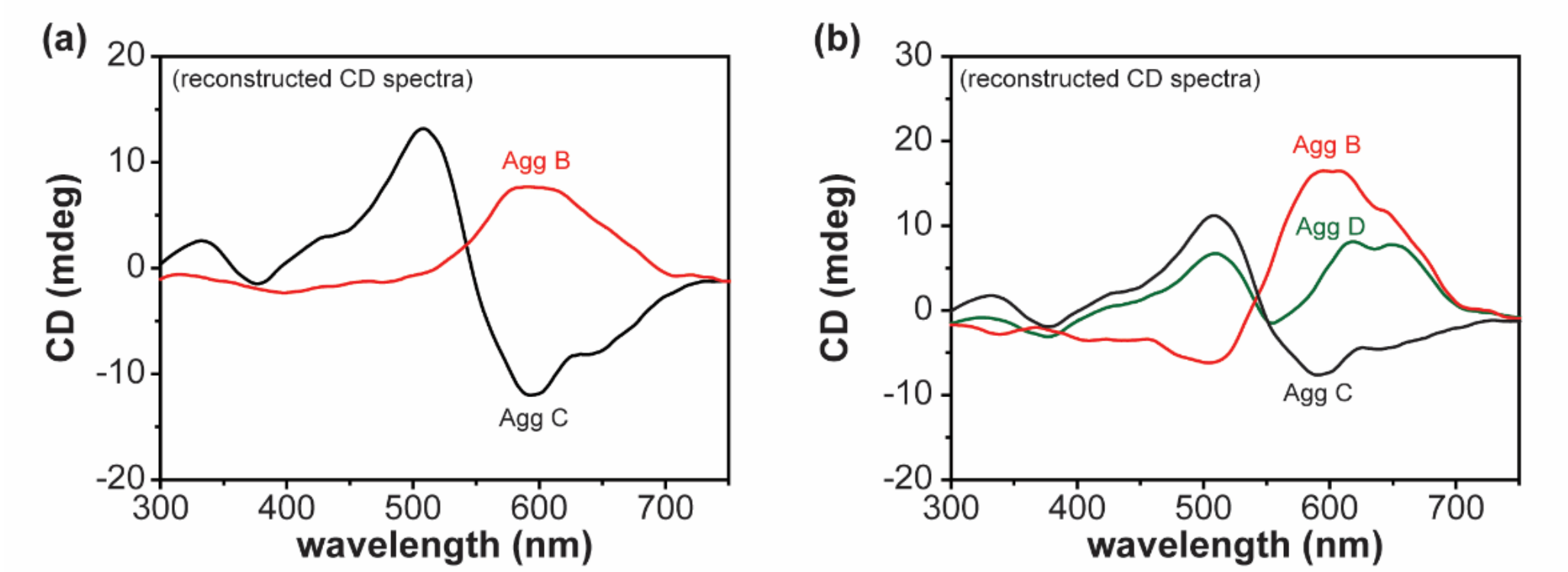

Figure S29. Reconstructed spectra of a) **Agg B** formed at 30 °C transformed to **Agg C** by applying sonication, and b) **Agg D** converted into **Agg B** and **Agg C** by sonication at different temperatures.

8. Supplementary videos

9. Supplementary references

[1]A. M. Garcia and A. Ruiz-Carretero, *Chem. Commun.*, 2022, **58**, 529–532.
