# Supplementary material for: From helices to superhelices through hierarchical assembly across competing pathways
Source: Nat Commun. 2026 Aug 11;17:9619. doi: 10.1038/s41467-026-76656-4 (PMC13554182; doi:10.1038/s41467-026-76656-4)
Supplement: Supplementary file 2 [file 41467_2026_76656_MOESM2_ESM.pdf]

Description of Additional Supplementary Information

Supplementary Video S1. A cropped section of superresolution z-stack of **Agg C** was reconstructed in 3D using ImageJ (3D Viewer plugin). The video shows a 360 degree rotation showing the helicity of the aggregate. See Figure S2 for a still 2D image.

Supplementary Video S2. A cropped section of superresolution z-stack of **Agg B** was reconstructed in 3D using ImageJ (3D Viewer plugin). The video shows a 360 degree rotation showing the helicity of the aggregate. See Figure S2 for a still 2D image.
