# Supplementary material for: From helices to superhelices through hierarchical assembly across competing pathways
Source: Nat Commun. 2026 Aug 11;17:9619. doi: 10.1038/s41467-026-76656-4 (PMC13554182; doi:10.1038/s41467-026-76656-4)
Supplement: Supplementary file 3 [file 41467_2026_76656_MOESM5_ESM.pdf]

nature portfolio

Peer Review File

From Helices to Superhelices through Hierarchical Assembly across Competing Pathways

Corresponding Author: Dr Amparo Ruiz Carretero

Parts of this Peer Review File have been redacted as indicated to remove third-party material.

This file contains all reviewer reports in order by version, followed by all author rebuttals in order by version.

A version of this paper was originally rejected for publication by Nature Communications, however that decision was reconsidered after appeal by the authors.

Version 0:

Reviewer comments:

Reviewer #1

(Remarks to the Author)
The authors report the characterization of self-assembled aggregates formed from a particular thiophene oligomer. Four different supramolecular species have been identified together with some insight on their relative stabilities and interconversion pathways. While the results are of interest to specialists in the field, I do not think the manuscript is suitable for publication in Nature Communications. First, some technical questions have to be clarified (see below). Second, even if the conclusions can be confirmed, it is difficult to see how the specific results described can be of general value and how they would translate in terms of functionality (besides CD signals).

The ITC experiments performed by the authors are potentially very informative, but I would like to stress possible pitfalls that need to be excluded.
-the authors ascertain that the measured enthalpies are obtained "without further assumptions". This is actually not true. In fact, the authors make the implicit assumption that their systems are fully assembled at the starting concentration (10 or 5 microM). To prove it the authors could check if the CD data is concentration independent above 10microM, which cannot be ascertained from Fig. S8.
-most importantly, performing ITC experiments with solvent mixtures is very tricky as a slight solvent composition deviation between samples that are in the syringe and in the cell will lead to a signal due to dilution of the DCE/MCH mixture. In view of the very small signals detected, this possible artifact has to be specifically excluded before any conclusion can be drawn. The fact that the authors performed experiments in triplicates is a good thing but they should mention whether these triplicates were made from 3 independent solutions or from aliquots coming from the same solution. Only triplicates from independent solutions can prove that experimental deviations in solvent composition does not introduce significant errors in the final results.

In addition, the authors should pay attention to the following minor details:
Page 5: the authors mention their ITC experiment is "leading to dilution by a factor 170". However, according to supporting information the experiment consists in injecting 20 microL into 1.4 mL i.e. a factor of 70.

Page 6: I disagree with the statement that "We can, however, exclude that Agg D is a true thermodynamic equilibrium, since its heating/cooling curves show large hysteresis". The hysteresis is only sign that the assembly is nucleated. The slow evolution at 15°C from 6 to 10mdeg is a sign that the assembly process was not complete right after cooling at 1K/min. The question is how much time elapsed between the end of cooling and the start of heating? If it was only a few minutes then the final equilibrium may have been reached. If it was several hours then I agree that Agg D is not at thermodynamic equilibrium. In fact, the time evolution of the CD signal after the cooling step would be most informative.

Page 7: the authors write "we expected Agg A to evolve to B, C, or D, but this is not the case". The authors should describe the experiment they performed to reach this conclusion (concentration, temperature, time).

Page 8: the authors speculate that "Agg D is a superhelix of Agg C, since the latter is accompanied with a chirality inversion". I do not understand the rational. One could state with the same reasoning from the CD data that C is a superhelix of B...

Fig. S14: The figure caption is unclear. Was the cooling curve measured before or after the heating curve? Moreover, the phrasing "Changes in the CD signal at 658 nm of Agg D upon cooling " is confusing because Agg D is obtained at the lowest temperature and is therefore certainly not cooled. It should be "Changes in the CD signal at 658 nm of monomer QRD upon cooling" or "Changes in the CD signal at 658 nm of Agg D upon heating ". Finally, the solvent composition should be mentioned.

Reviewer #2

(Remarks to the Author)
Hermans, Ruiz-Carretero, et al. reported on the pathway complexity involved in the self-assembly of a quinquethiophene-rhodanine (QRD) derivative in organic media, resulting in four distinct aggregate forms, each exhibiting unique morphologies and optoelectronic properties. In a mixed DCE/MCH solvent system, varying the preparation procedures enabled the isolation of aggregates Agg A through Agg D, which were characterized using UV-Vis and CD spectroscopy. Furthermore, the stability and interconversion of these self-assembled forms were investigated, with the relative proportions of each form determined by deconvolution of the CD spectra. Notably, the addition of seeds effectively accelerated the conversion of on-pathway species to the superhelical Agg D form, while a combination of thermal and sonication treatments could induce the disassembly of Agg D into Agg B and Agg C.
While this manuscript demonstrates the complexity and interrelationships among different self-assembly pathways, the novelty and significance of these supramolecular systems remain unsubstantiated. Additionally, the characterization presented (and thus the overall content) is insufficient. In light of these concerns, the manuscript is not suitable for publication in Nature Communications and it is recommended to transfer to a specific journal e.g. Commun. Chem.

The following comments are suggested for consideration:
1. The concepts of pathway complexity and seeded supramolecular polymerization have been extensively developed over the past decade. What advances and significance does the supramolecular system described in this article offer compared to existing systems and methodologies? The authors are strongly encouraged to reorganize the introduction to clearly highlight the novelty and unique contributions of their work.

2. In this manuscript, the authors primarily investigate the assemblies using CD spectroscopy. While this allows differentiation of the pathways, the manuscript lacks a comprehensive understanding of the underlying self-assembly processes. Specifically, how are the molecules arranged within each type of assembly? Since molecular packing has a profound impact on assembly morphology, it should be characterized in greater detail. Furthermore, given the observed chirality amplification and inversion, the packing modes in both the helix and superhelix forms warrant thorough discussion.

3. Building on the previous point, the reviewer notes that the amide groups in the alanine motifs likely play a crucial role in chirality transfer and pathway determination. Additional techniques such as NMR and IR spectroscopy should be employed to characterize the hydrogen bonding interactions. How does the packing of hydrogen bonds correlate with the different assembly pathways?

4. The authors utilized distinct preparation methods to obtain different aggregates. What can be obtained if fast cooling was performed and without sonication (as analog of method for Agg D)? For Agg B, the procedure of sonication (in room temperature bath) of high temperature solutions is unusual. What influence can this cause comparing to the two cooling processes (fast and slow cooling)?

5. In Figures 2a and 2b, why does the CD signal around 600 nm increase from day 3 to day 7, even though the fraction of Agg B is at its lowest on day 7? Please clarify this apparent discrepancy between the CD signal intensity and the aggregate composition.

6. It is recommended that the cross-on-dot symbols in Figures 2b (15 µM concentration) and 2d (20 µM concentration) be modified, as they are difficult to distinguish from solid dots. Please consider using more distinct markers to improve clarity.

7. On page 8, the manuscript states, “Agg A should be a superhelix of Agg B, but cannot be formed from it since it is energetically uphill.” Is there any evidence supporting this argument? If Agg A is directly related to Agg B as its superhelix form, why does the energy of Agg A differ so significantly compared to Agg D as the superhelix of Agg C?

8. The manuscript claims, “Agg B—unable to convert to Agg D—acts as a monomer reservoir, accelerating Agg C conversion into superhelix Agg D.” Is there any evidence supporting this process, particularly the disassembly of Agg B? The deconvolution analysis was only performed for equilibrium solutions; please provide additional data or discussion to substantiate this claim.

9. Minor Typographical Errors

• Page 5: In the sentence "… confocal microscopy (Figure S2) Indeed, …" a period is missing after the parenthesis.
• Page 9: In the sentence "Sonication at 10 °C for 30 min. provoked the conversion …" there is an unnecessary period after "min".

Reviewer #3

(Remarks to the Author)
Title: Pathway selection into 4 isolatable helical supramolecular aggregates

This manuscript describes the self-assembly of a π-conjugated chiral quinquethiophene–rhodanine system driven by π–π stacking and hydrogen bonding, resulting in a complex energy landscape. By employing four preparation protocols involving mechanical and thermal perturbations, the authors report the formation and isolation of four chiral supramolecular aggregates with distinct chiroptical, hierarchical, and morphological features. Measurements of assembly enthalpies by isothermal titration calorimetry, together with spontaneous, induced, and seeded interconversion experiments, are used to construct an energy landscape comprising on- and off-pathway relationships among the aggregates. The results have the potential to advance the area of chiral supramolecular materials. However, the manuscript is not suitable for publication in Nature Communications in its current form, as several issues need to be addressed to substantiate the mechanistic claims and improve clarity.

The seeding experiments are an important part of the interpretation of the energy landscape. Given the metastability and interconvertibility of the aggregates, the preparation, purity, and stability of the seeds are crucial points. However, the manuscript does not describe the preparation protocol and characterization of the seeds. The authors should provide the seed preparation protocol and include AFM images, size distributions, and time-dependent CD data of the seed solutions to ensure their purity and stability over the timescales relevant to the experiments.

The following additional points should also be addressed properly to improve the quality of the manuscript further.

(i) The introduction section does not adequately convey the significance of the work to the readers. Given that the title emphasizes the achievement of "4 isolatable chiral aggregates", the introduction should clearly state the fundamental challenges associated with isolating multiple (4 here) chiral aggregates from a single monomer, explain why this has not been achieved previously, and clarify how the present work strategically overcomes these limitations, beyond empirical protocol variation. Addressing these points would substantially strengthen the motivation and framing of the study.

(ii) The AFM images provided lack sufficient resolution to clearly visualize the aggregates. Improved/higher-magnification AFM images should be included to enable a more reliable structural assessment. In addition, the analysis is currently limited to fiber heights and lengths of the aggregates. Given that all four isolatable aggregates are described as helical in nature, their helical pitch distribution should be included for comparison among the aggregates.

(iii) During the preparation of the bundled aggregates (Agg A and Agg D), it remains unanswered when and how bundling occurs. Does bundling arise at the initial or later stage of assembly? The manuscript does not provide any evidence to clarify this mechanism. What kind of interactions mediate the association of individual helical fibers into bundled or superhelical structures? To confirm bundling as a hierarchical process, 1D and 2D X-ray diffraction experiments are recommended to probe whether there is any subtle difference in internal molecular packing between the helical and superhelical aggregates.

(iv) Under external perturbation—such as mechanical agitation or changes in solvent composition—do the bundled aggregates undergo stepwise debundling followed by molecular disassembly? In particular, the authors should clarify whether the conversion of Agg D into Agg B or Agg C under sonication proceeds via partial debundling (fragmentation) or via complete disassembly into monomers followed by reassembly. If disassembly into monomers is involved, why does kinetically trapped Agg A not form? To get insight into the formation of Agg A, it is essential to characterize the species formed in 1:9 dichloroethane/methylcyclohexane solution by injection at room temperature. This point is critical, as sonication of this solution produces Agg A, whereas all other aggregates are obtained starting from the monomeric solutions prepared at elevated temperature.

(v) In Fig. 3c, Agg B (91%) exhibits lower CD intensity and different spectral features compared to Agg B (81%) in Fig. 3d, although the solutions have identical total concentrations. The origin of this discrepancy should be clarified.

(vi) In the time-dependent evolution of CD of Agg B and Agg C, is there an observable lag phase at the initial stage? If so, how does the lag phase depend on concentration?

(vii) The heating-cooling curves of Agg D show a slightly negative value of the CD signal at high temperature instead of zero (Fig. S14). Does the solution contain some residual aggregates?

Version 1:

Reviewer comments:

Reviewer #1

(Remarks to the Author)
I am fully satisfied with the modifications concerning my technical questions. However, the authors have not replied to my main concern. It is still difficult to see how the specific results described can be of general value and how they would translate in terms of functionality (besides CD signals). I therefore cannot support publication in this journal.

Reviewer #2

(Remarks to the Author)
After the current revision, the originality, novelty, and contribution of this work were presented more clearly in the updated introduction. The authors have demonstrated the incorporations of secondary nucleation into the pathway complexity, in generating distinguishable and isolatable structures supported by detailed CD discussions. The insightful view of the helix formation, especially the newly added experiments regarding pathway of Agg A to Agg B, is fruitful for understanding the energy landscape of the current system. In considering the improved experimental results and discussion, the reviewer suggests addressing some remaining issues before considering acceptance in the in Nat. Commun., as mentioned below.

For XRD measurements which can obtain crucial information for aggregation patterns, the authors did not provide deep insight into the structures. In PXRD experiments, it is suggested that a thinner glass substrate would be used to reduce background signals, and extended exposure time the increase the sample signals with reduced background signal.

Importantly, the authors should provide detailed experiment setups (X-ray source, exposure time, etc.) and sample preparation protocols for clear understanding of the provided data, which may give direct hint on molecular arrangements.

The SAXS results presented are inadequate. A rapid attenuation of signal even below region of $q < 0.1$ Å-1 suggests poor structure detected, which is in sharp contrast to the well-defined morphologies reported by microscopies (for reference see Nat. Commun. 2025, 16, 9316). The authors should re-examine the experiment setup and sample preparation method, and provide detailed fitting of their improved data.

In FT-IR results presented, why different concentrations of aggregates were used for different solvent mixtures? Also, a 20 µM solution may be too dilute to afford a good signal-to-noise ratio. In summary, detailed structural characterizations can illustrate the intrinsic properties of distinguishable helical structures, and can further resolve the bundling and debundling processes of superhelix observed.

Reviewer #3

(Remarks to the Author)
Title: From Helices to Superhelices: Hierarchical Assembly across Competing Pathways

General Assessment: I appreciate the authors' effort in revising the manuscript entitled "From Helices to Superhelices: Hierarchical Assembly across Competing Pathways" in response to my previous comments. The revised version shows noticeable improvement, particularly in the introduction section, which now presents a clearer and more relevant context for the study. As encouraged in my previous report, the authors have undertaken a more detailed investigation into the role of secondary processes in the hierarchical assembly mechanism, and the results suggest that secondary nucleation plays a dominant role in this system. However, several major discrepancies remain in the revised manuscript. The following points should be carefully addressed to strengthen the credibility of the work further.

Specific Comments:
(i) The authors state that the conditions leading to the formation of Agg D are consistent with hierarchical growth processes favoring secondary nucleation and subsequent fiber bundling. However, kinetic analysis is provided only for Agg D, while analogous analyses for the other aggregates are avoided. A comparative scaling analysis is necessary to elucidate the relative contributions of primary and secondary nucleation pathways governing the formation of distinct hierarchical morphologies (helices vs. superhelices i.e., Agg A vs. Agg B and Agg C vs. Agg D). The corresponding scaling plots and a schematic illustration of the secondary nucleation mechanism-mediated hierarchical self-assembly mechanism should be clearly shown in the main manuscript.

(ii) The authors rationalize chirality inversion during helix-to-superhelix transitions in terms of stress relaxation of the resulting helical structure (energetic reasons). This interpretation is consistent with the transition from helices Agg C ($\Delta H_{assem, C} = -176.7 \pm 3.5$ kJ/mol) to superhelices Agg D transition (superhelices: $\Delta H_{assem, D} = -221.2 \pm 13.3$ kJ/mol), where a clear enthalpic stabilization is observed. However, this rationale is not consistently supported across the system. The transition from helices Agg B ($\Delta H_{assem, B} = -159.1 \pm 1.7$ kJ/mol) to superhelices Agg A ($\Delta H_{assem, A} = -139.9 \pm 3.2$ kJ/mol) is associated with an enthalpic penalty. This inconsistency should be addressed to substantiate the authors' interpretation.

(iii) The authors claim that the transition from Agg B to Agg C is an off-pathway process occurring via the monomer, a conclusion supported by the observed enhancement of the transition rate at lower concentrations. However, the energy landscape presented in Figure 1d shows a direct connectivity between these two states, implying an on-pathway relationship. This inconsistency between the mechanistic interpretation and its graphical representation is problematic. If the

interconversion indeed occurs via monomer, the energy landscape should explicitly reflect this by depicting a pathway that traverses the monomeric state rather than a direct connection between Agg B and Agg C. The authors should revise Figure 1d accordingly to ensure consistency with their proposed mechanism.

(iv) The manuscript highlights the “long-lived nature” of the isolated aggregates as a key feature that enables direct calorimetric determination of their assembly enthalpies and the construction of a quantitative enthalpic energy landscape. While this represents an impressive experimental achievement, the authors do not provide any discussion regarding the molecular origin of this behavior. It remains unclear what specific features of the molecular design or self-assembly endow such kinetic stability, allowing multiple distinct states to persist under these conditions. The authors should therefore provide a rationale or at least a plausible hypothesis addressing the origin of this long-lived behavior. Such a discussion would significantly enhance the broader impact of the work and offer valuable insights for the future design of systems with similarly well-defined and long-lived metastable states.

Minor comment: γ is the scaling exponent. It should be mentioned with the scaling equation.

Version 2:

Reviewer comments:

Reviewer #2

(Remarks to the Author)
The reviewer is satisfied with the efforts of authors concerning xrd-related results. Broadened peaks can be distinguished from PXRD experiments, and SAXS data with absence of oscillation features may indicate the poor long-range order within the assembled structures. It is still obscure to the reviewer how an amorphous system can adopt precise pathway selection in generating isolable and long-live species, with limited structural identifications only accessing the morphologies. Even the authors claimed that secondary nucleation is a dominating process, the correlation of sample preparation procedures and the assembly pathway selection should be discussed, considering general impact for orchestrating complex but controllable systems.

Reviewer #3

(Remarks to the Author)
I suggested the evaluation of the relative contributions of primary and secondary nucleation processes in the formation of the different aggregates (Agg A–D). The authors have clarified that experimental constraints associated with the preparation protocols prevented kinetic analyses of Agg A–C. Therefore, I do not wish to comment further on this point.

However, my original comment also included a schematic illustration of the proposed secondary nucleation-mediated hierarchical assembly pathway leading to the double-helical Agg D structure. There should be no limitation preventing the inclusion of such a schematic, for example, below Figure 1D. This would help readers visualize the proposed assembly mechanism and strengthen the discussion of Agg D formation.

Version 3:

Reviewer comments:

Reviewer #2

(Remarks to the Author)
In the revised manuscript the authors have addressed all the reviewers' comments, therefore I suggest this manuscript to be accepted in the current form.

Open Access This Peer Review File is licensed under a Creative Commons Attribution 4.0 International License, which permits use, sharing, adaptation, distribution and reproduction in any medium or format, as long as you give appropriate credit to the original author(s) and the source, provide a link to the Creative Commons license, and indicate if changes were made.
In cases where reviewers are anonymous, credit should be given to 'Anonymous Referee' and the source.
The images or other third party material in this Peer Review File are included in the article's Creative Commons license, unless indicated otherwise in a credit line to the material. If material is not included in the article's Creative Commons license and your intended use is not permitted by statutory regulation or exceeds the permitted use, you will need to obtain permission directly from the copyright holder.
To view a copy of this license, visit https://creativecommons.org/licenses/by/4.0/

Point by point answers to referees' comments:

Reviewer #1 (Remarks to the Author):

The authors report the characterization of self-assembled aggregates formed from a particular thiophene oligomer. Four different supramolecular species have been identified together with some insight on their relative stabilities and interconversion pathways. While the results are of interest to specialists in the field, I do not think the manuscript is suitable for publication in Nature Communications. First, some technical questions have to be clarified (see below). Second, even if the conclusions can be confirmed, it is difficult to see how the specific results described can be of general value and how they would translate in terms of functionality (besides CD signals).

The ITC experiments performed by the authors are potentially very informative, but I would like to stress possible pitfalls that need to be excluded.

The authors ascertain that the measured enthalpies are obtained "without further assumptions". This is actually not true. In fact, the authors make the implicit assumption that their systems are fully assembled at the starting concentration (10 or 5 microM). To prove it the authors could check if the CD data is concentration independent above 10microM, which cannot be ascertained from Fig. S8.

Answer: We thank the referee for the insightful comments on our work.

Regarding the reviewer's concern on the assumptions about the ITC experiments, we have included additional data to clarify this point. We have confirmed that our systems are fully assembled at the starting concentration (10 or 5 μM) by plotting molar ellipticity at concentrations ranging from 1 μM to 20 μM. The results show no spectral changes once the concentration is 5 μM or higher. We have substituted Figure 8 by a new one, currently Figure S13 in the updated version (see below), including the CD spectra and the molar ellipticity graphs.

This change in Figure S8 has been highlighted in yellow in the SI and we have incorporated an additional sentence highlighted in yellow in the main text, page 6: "(as verified by CD spectroscopy and plotting the molar ellipticity for each of the aggregates, Figure S13)".

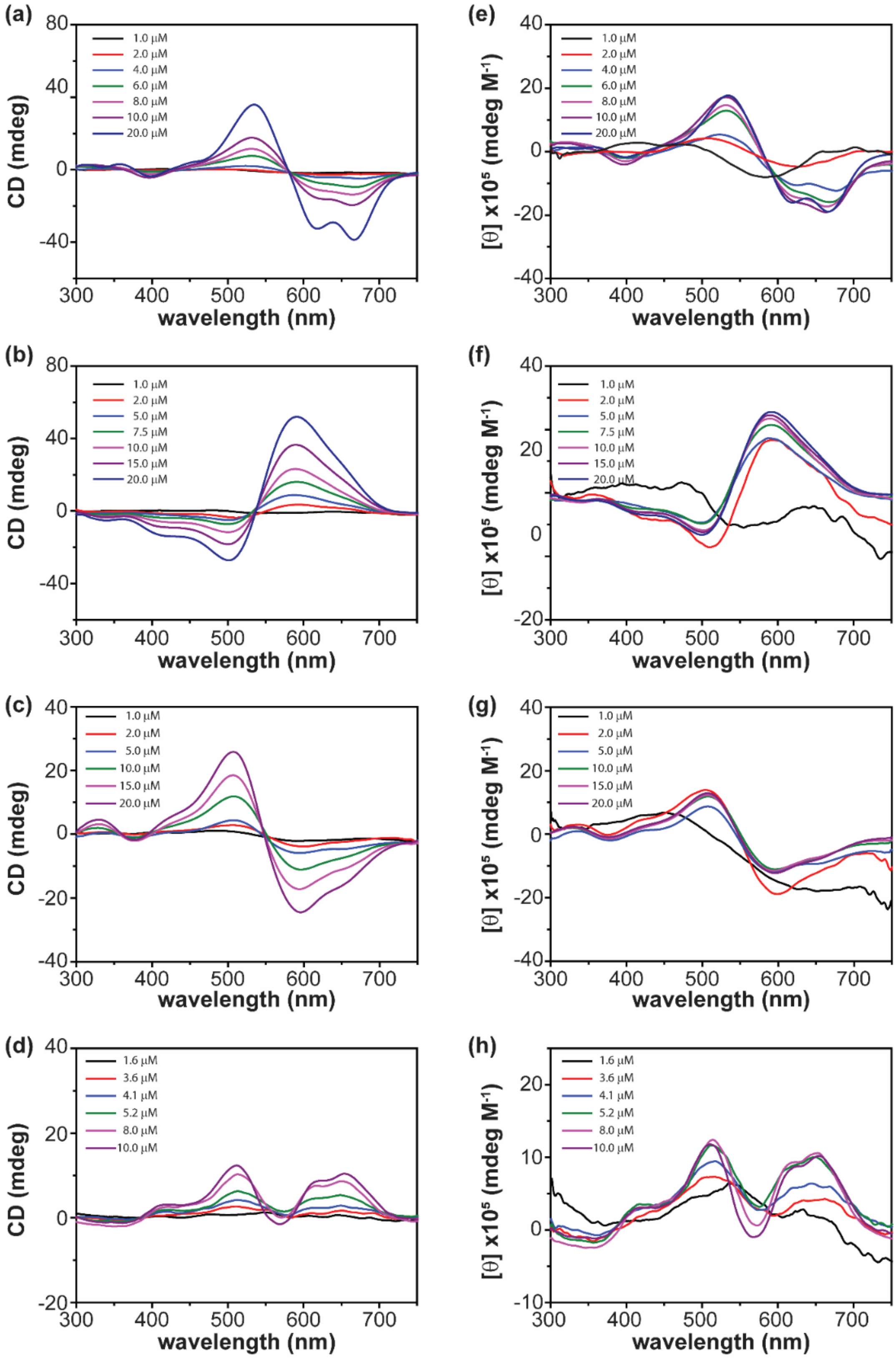

New Figure S13. Concentration dependent CD spectra in 1/9 DCE/MCH (l = 1 cm, 20 °C); a and e) **Agg A**, b and f) **Agg B**, c and g) **Agg C**, and d and h) **Agg D** plotted with mdeg and molar ellipticity of CD intensity, respectively.

Most importantly, performing ITC experiments with solvent mixtures is very tricky as a slight solvent composition deviation between samples that are in the syringe and in the cell will lead to a signal due to dilution of the DCE/MCH mixture. In view of the very small signals detected, this possible artifact has to be specifically excluded before any conclusion can be drawn. The fact that the authors performed experiments in triplicates is a good thing but they should mention whether these triplicates were made from 3 independent solutions or from aliquots coming from the same solution. Only triplicates from independent solutions can prove that experimental deviations in solvent composition does not introduce significant errors in the final results.

Answer: We thank the referee for raising this important point. We fully agree that ITC measurements in mixed solvent systems require particular care, as even minor deviations in solvent composition between the syringe and the cell may generate heat signals that could interfere with the interpretation of small dilution enthalpies.

First, we would like to clarify that all ITC experiments were performed in triplicate using three independently prepared solutions, not aliquots from a single stock solution. For each replicate, the solvent mixture and the aggregate solutions were prepared separately by independent weighing using an analytical balance. The reported ΔH values (Previous Table S1 and now Table S2) therefore, reflect reproducibility across independently prepared samples.

To rigorously exclude artifacts arising from solvent mismatch or dilution effects, we performed a series of dedicated control experiments, now explicitly described in the revised Supporting Information (Section 4). An additional sentence has been included in page 7 of the main text to guide the reader to the SI (highlighted in yellow: "(See section 4 in SI for all the ITC sample preparation details and control experiments, Figures S10-S17 and Tables S1 and S2)."

We have included the following information in the revised version of the SI:

1. **Solvent-solvent titration (same vial).** The ITC cell and syringe were filled with the identical 1:9 DCE/MCH solution (prepared gravimetrically). The titration of the solvent into itself produced no detectable heat signal, confirming negligible intrinsic heat of dilution (Figure S10a).

2. **Solvent-solvent titration (independent preparations).** Two separate 1:9 DCE/MCH solutions were independently prepared by weighing (1250 mg DCE and 6930 mg MCH for the 10% v/v mixture). One solution was placed in the cell and the other in the syringe. No significant heat signal was observed. These experiments were repeated twice, confirming that independent preparation of the solvent mixture does not introduce measurable calorimetric artifacts.

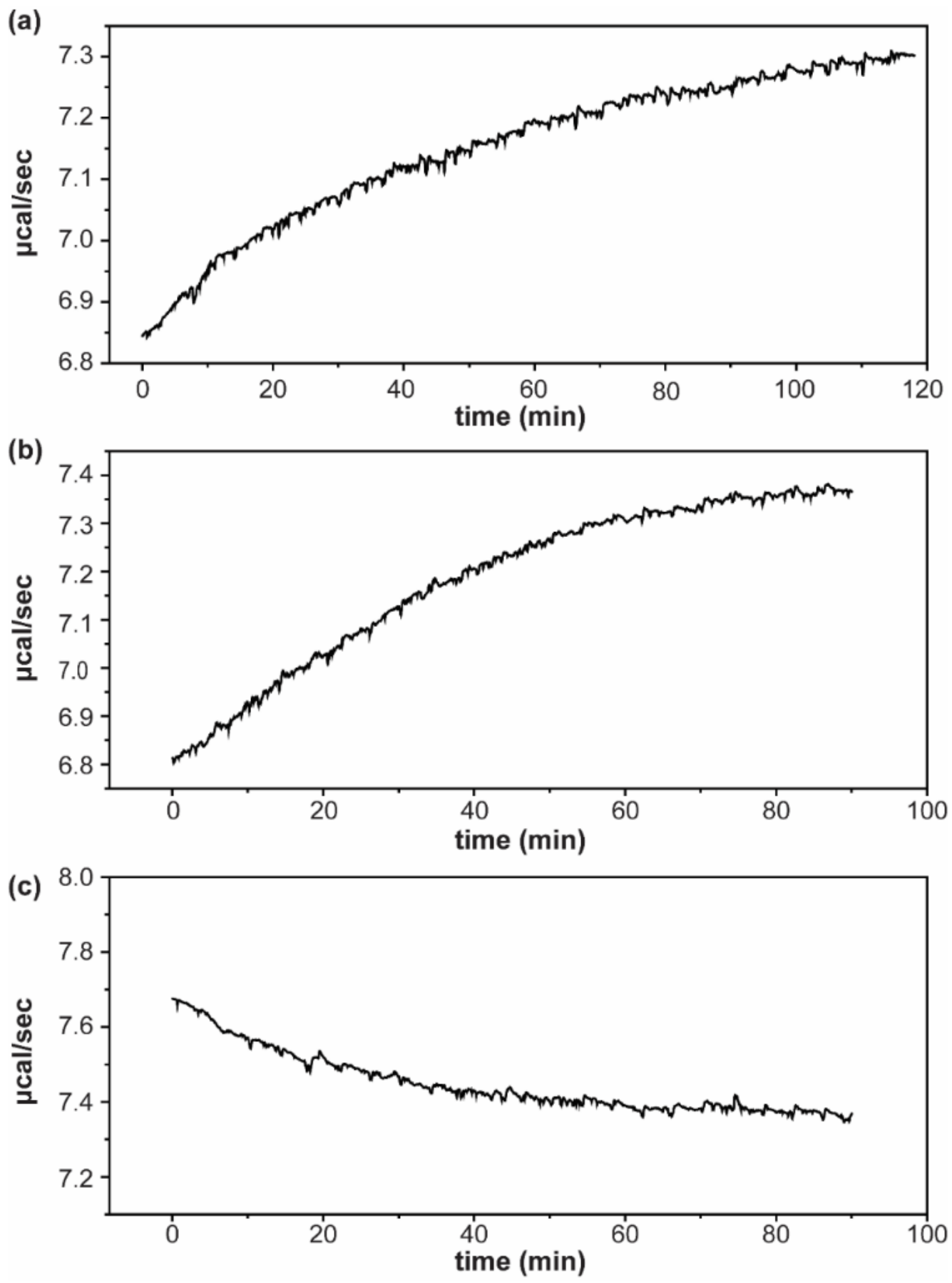

New Figure S10. Solvent (10% DCE/MCH; sample cell)-solvent (10% DCE/MCH; syringe) titration a) from same vial, b) and c) separately prepared from different vials.

3. **Effect of heating and sonication on solvent composition**. Because the aggregates preparation involves heating and/or sonication (see Section 2 of the SI), we verified that these treatments do not alter solvent composition. A 1:9 DCE/MCH mixture was subjected to the same thermal and sonication procedures used for aggregate preparation and then tested by solvent–solvent ITC titration. No significant heat effects were detected, demonstrating that these treatments do not measurably change the solvent composition

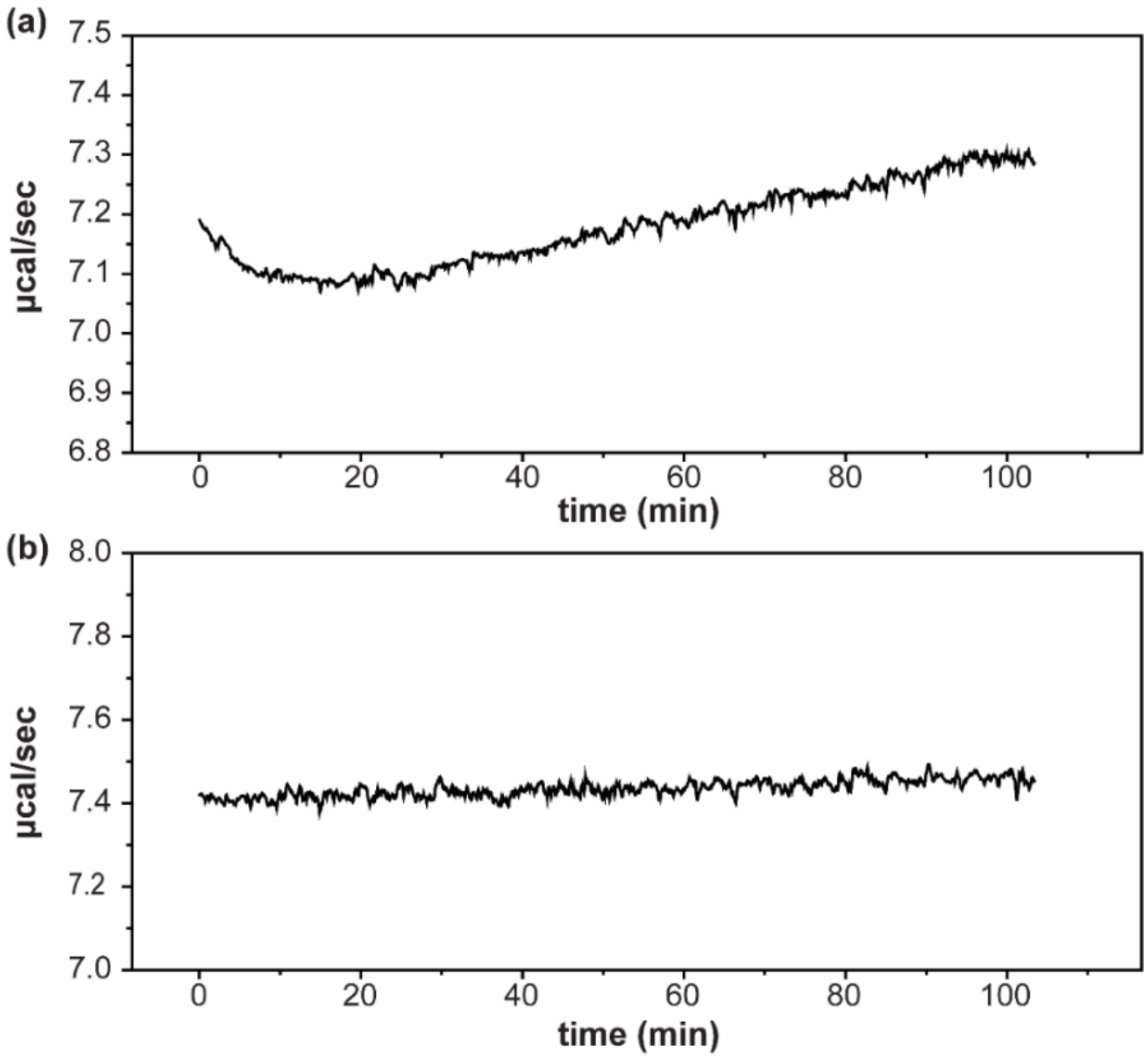

New Figure S11. a) Solvent-solvent titration before applying external stimuli, and b) Solvent-solvent titration after applying external stimuli.

4. **Deliberate solvent mismatch experiments.** To evaluate the sensitivity of the system to composition deviations, we intentionally introduced small differences in DCE content (9.95%, 10.05%, 10.1%) between syringe and cell solutions (New Table S1). In these cases, clear exothermic heat signals were observed, and their magnitude increased with increasing composition mismatch These control experiments demonstrate that the ITC setup is indeed sensitive to small solvent. composition differences. Importantly, the absence of such signals in our standard experiments confirms that solvent mismatch does not contribute to the measured aggregate dilution enthalpies.

New Table S1. Solvent mismatch tests.

	10 mL		Weight (mg)	
%DCE/MCH	DCE	MCH	DCE	MCH
10.1	1.01	8.99	1262.5	6922.3
10.05	1.005	8.995	1256.25	6926.15
**10**	**1**	**9**	**1250.0**	**6930**
9.95	0.995	9.005	1243.75	6933.85

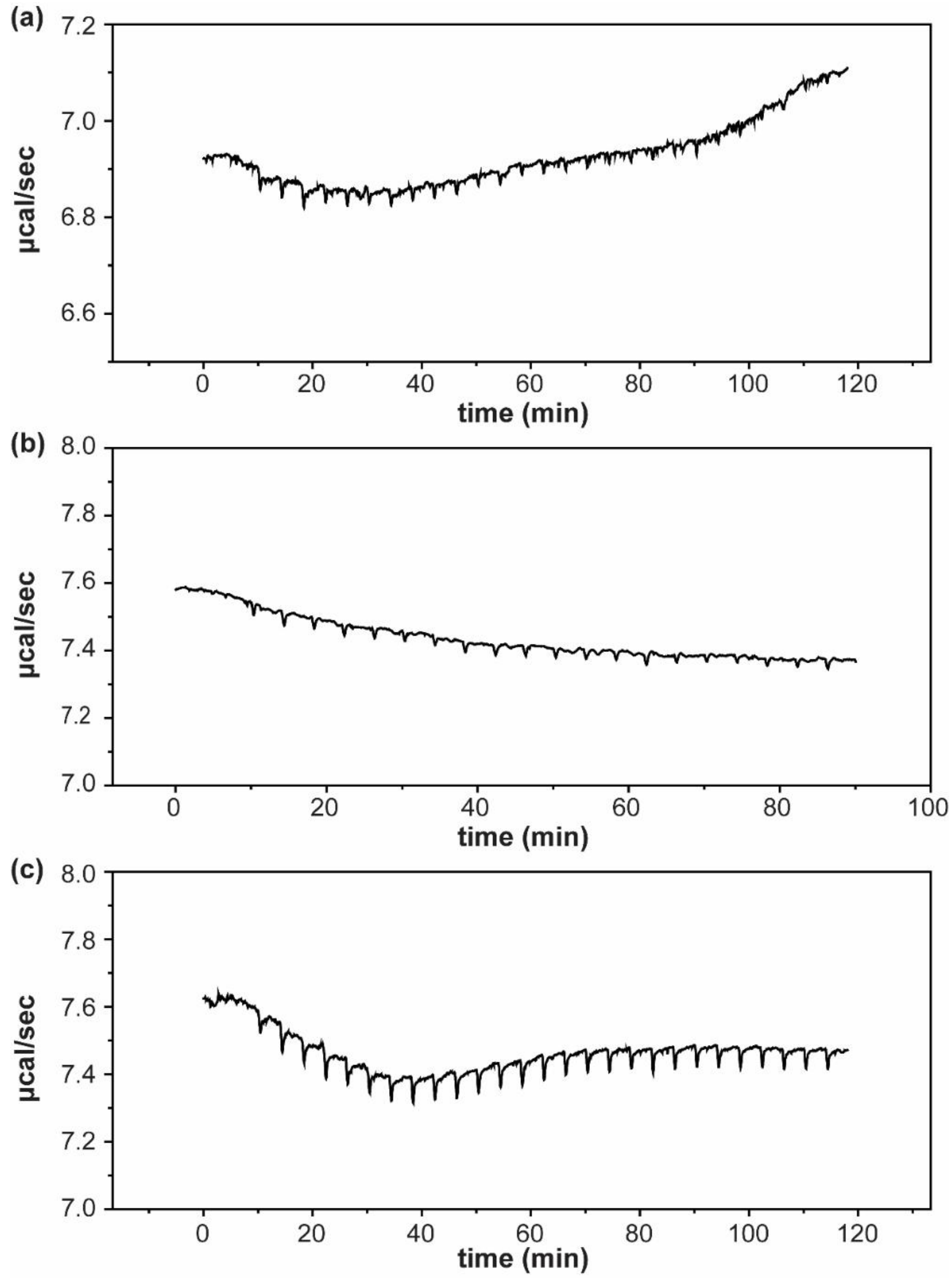

New Figure S12. Solvent-solvent titration with different solvent ratios: a) 9.95% DCE/MCH, b)10.05% DCE/MCH and c) 10.1% DCE/MCH

Finally, we note that the aggregate dilution experiments (former Figures S9–S13, current Figures S14-S17) were performed under identical solvent conditions in the syringe and cell, and that the measured endothermic signals are consistent with full disassembly upon dilution (as independently verified by CD spectroscopy in the main text). The magnitude and reproducibility of the signals across three independently prepared samples further confirm their reliability.

In summary, (i) all triplicates were performed from independently prepared solutions, and (ii) multiple dedicated control experiments, including solvent–solvent titrations, independent gravimetric preparations, external stimulus controls, and deliberate mismatch tests, demonstrate that solvent composition artifacts do not affect the reported ITC results.

- In addition, the authors should pay attention to the following minor details: Page 5: the authors mention their ITC experiment is "leading to dilution by a factor 170". However, according to supporting information the experiment consists in injecting 20 microL into 1.4 mL i.e. a factor of 70.

Answer: we apologize for the mistake. The change is highlighted in yellow on page 6 of the main text: “leading to dilution by a factor 70”.

Page 6: I disagree with the statement that "We can, however, exclude that Agg D is a true thermodynamic equilibrium, since its heating/cooling curves show large hysteresis". The hysteresis is only sign that the assembly is nucleated. The slow evolution at 15°C from 6 to 10mdeg is a sign that the assembly process was not complete right after cooling at 1K/min. The question is how much time elapsed between the end of cooling and the start of heating? If it was only a few minutes then the final equilibrium may have been reached. If it was several hours then I agree that Agg D is not at thermodynamic equilibrium. In fact, the time evolution of the CD signal after the cooling step would be most informative.

Answer: We thank the reviewer for this thoughtful comment. We agree that hysteresis alone does not necessarily exclude thermodynamic equilibrium, as it can indeed arise from nucleation-controlled assembly processes. We therefore recognize that our original wording could be misleading and have revised the sentence accordingly in the manuscript. It is highlighted in yellow on page 8 of the main text: "Although **Agg D** displays the most favourable assembly enthalpy, the pronounced thermal hysteresis and the time-dependent evolution of the CD signal after cooling indicate that its formation proceeds through kinetically controlled, nucleation-limited pathways rather than immediate thermodynamic equilibration (Figure S18).[45,46] Thus, these measurements indicate that **Agg D** is much more stable than **Agg A**, whereas **Agg C** is slightly more stable than **Agg B**, with differences in energy that disclose potential transitions between these aggregates, as well as the presence of competing pathways".

The statement has now been reformulated to clarify that the large hysteresis ($\Delta T > 30$ °C) reflects the inability of the system to equilibrate efficiently upon reversing the thermal ramp, indicating the presence of kinetic barriers and metastable intermediates during the assembly/disassembly processes. Our intention was not to claim that **Agg D** cannot represent the lowest enthalpic state, but rather to emphasize that its formation occurs under kinetic control with competing pathways.

In Answer to the reviewer's important question regarding the time elapsed between cooling and reheating: the cooling was performed at 1 K min^{-1} to 15 °C, and the heating ramp was initiated shortly thereafter (within minutes). We agree that this does not necessarily allow full equilibration.

To address this point directly, we have now monitored the time evolution of the CD signal after the cooling step at constant temperature (15 °C). The kinetic trace (now included as new Figure S18) shows that the CD signal continues to evolve after the cooling ramp, reaching a plateau within approximately 1 hour at 10 μM. This confirms that the assembly process is not fully complete immediately after cooling at 1 K min^{-1}, in agreement with the reviewer's interpretation.

Furthermore, we quantified the lag time required to reach the final CD intensity corresponding to **Agg D**. The lag time is concentration dependent: increasing concentration shortens the lag time (New Figure S18f). This concentration dependence is consistent with a nucleation–elongation mechanism involving secondary nucleation processes, which is characteristic of cooperative superhelical formation.

We also attempted slower cooling ramps (0.1 K min^{-1}) to favor equilibration toward the thermodynamically most stable species. However, under these conditions, precipitation occurred, likely due to extensive superhelix formation, preventing reliable spectroscopic monitoring.

All together, these additional experiments support the view that while **Agg D** is the most enthalpically favored aggregate (as determined by ITC), its formation proceeds via a kinetically controlled, cooperative pathway with significant nucleation barriers. We have revised the manuscript text to reflect this more precise interpretation.

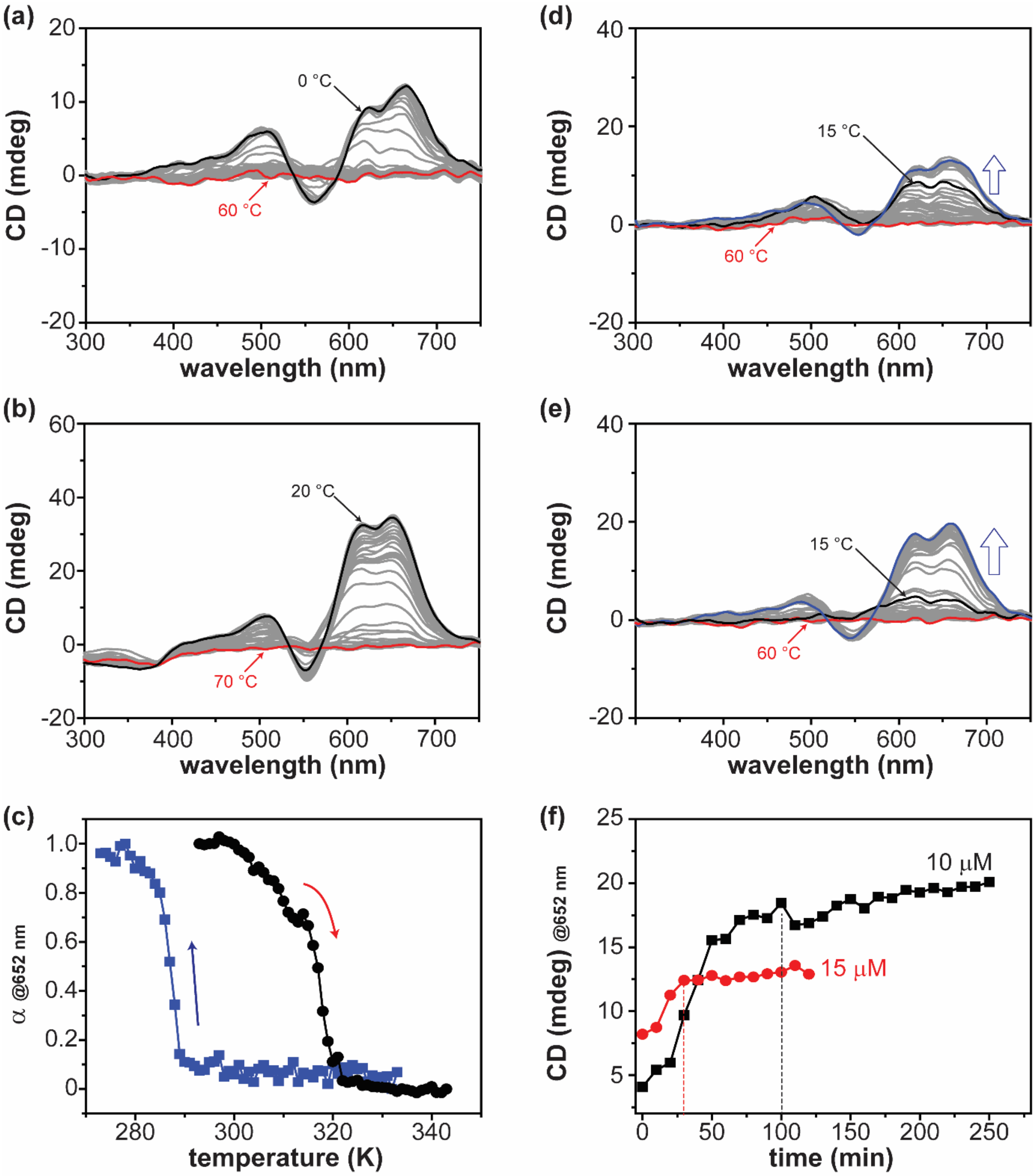

New Figure S18. Variable temperature CD spectra of QRD (10 uM in 10% DCE/MCH) a) cooling from 60 ºC to 0 ºC, b) heating from 20 ºCto 70 ºC, and c) degree of aggregation of heating and cooling process extracted from (a) and (b) monitored at 652 nm. (d) and (e) are variable temperature CD spectra of QRD cooling from 60 ºC to 15 ºC and time dependent kinetic experiment to grow **Agg D** at 15 ºC at different concentrations, (f) CD intensity monitored at 652 nm over time extracted from graph (d) and (e).

Page 7: the authors write "we expected Agg A to evolve to B, C, or D, but this is not the case". The authors should describe the experiment they performed to reach this conclusion (concentration, temperature, time).

Answer: We thank the reviewer for this important comment. We agree that the experimental conditions under which we concluded that **Agg A** does not spontaneously evolve directly into **Agg B**, **C**, or **D** should be explicitly described.

To investigate this, we performed time-dependent kinetic experiments on **Agg A** at different concentrations (5, 10, and 20 μM) in 1:9 DCE/MCH at room temperature. After preparation of **Agg A** under standard sonication conditions, CD spectra were recorded over 26 days. The time-resolved CD spectra were subsequently deconvoluted using linear combinations of the reference spectra of **Agg A–D**, allowing us to quantify the relative fraction of each aggregate as a function of time.

The results show clear concentration dependence:

- **At 5 μM**, **Agg A** decreases relatively rapidly. Deconvolution reveals that **Agg A** mainly converts into **Agg B** and **Agg C**. However, no significant direct formation of **Agg D** is observed at early stages. The conversion from **Agg B/C** to **Agg D** remains slow under these dilute conditions.
- **At 10 μM**, a similar trend is observed, although the conversion is slower than at 5 μM.
- **At 20 μM**, **Agg A** persists longer, and over time a measurable fraction of **Agg D** appears. At this higher concentration, the conversion from intermediate **Agg B/C** states toward **Agg D** becomes more significant.

We have included these results at the beginning of the section "spontaneous conversions" in the main text on pages 8-9 (highlighted in yellow): "A priori, we expected **Agg A** to evolve to **Agg B**, **Agg C**, or **Agg D**. To investigate this, we performed time-dependent experiments on **Agg A** at different concentrations (5, 10, and 20 μM) in 1:9 DCE/MCH at room temperature (Figure 2a,d and Table S3). After preparation of **Agg A** under standard sonication conditions, CD spectra were recorded over 26 days. The time-resolved CD spectra were subsequently deconvoluted using linear combinations of the reference spectra of **Agg A–D**, allowing us to quantify the relative fraction of each aggregate as a function of time. The results show clear concentration dependence: at 5 μM, **Agg A** decreases relatively rapidly. The spectral deconvolution reveals that **Agg A** mainly converts into **Agg B** and **Agg C**. However, no significant direct formation of **Agg D** is observed at early stages. The conversion from **Agg B/C** to **Agg D** remains slow under these dilute conditions. At 10 μM, a similar trend is observed, although the conversion is slower than at 5 μM. Finally, at 20 μM, **Agg A** persists longer, and over time a measurable fraction of **Agg D** appears. At this higher concentration, the conversion from intermediate **Agg B/C** toward **Agg D** becomes more significant.
Importantly, in none of the experiments we observed a direct, rapid conversion of **Agg A** into **Agg D**. Instead, **Agg A** first evolves into **Agg B** and/or **Agg C**, and only subsequently, under conditions where the **Agg B/C** → **Agg D** pathway is operative, does **Agg D** emerge. This behaviour implies that **Agg A** is not on the direct pathway toward **Agg D** but rather requires partial depolymerization and reorganization before accessing the **Agg B/C** → **Agg D** *on-pathway* landscape. As a reminder, *"on-pathway"* is a stepping stone toward the intended state, whereas *"off-pathway"* denotes a detour that traps monomers in a non-productive state. **Agg A** is thus *off-pathway* towards **Agg D**.[10,11]"

These time-dependent kinetic experiments (now explicitly described in the revised manuscript and shown in Figure for reviewer below, Current Figures 2a-2d of the revised main text) therefore substantiate our statement that **Agg A** does not directly evolve into **Agg B**, **Agg C**, or **Agg D** under the studied conditions, and instead behaves as a kinetically trapped state whose evolution proceeds via intermediate aggregates in a concentration-dependent manner.

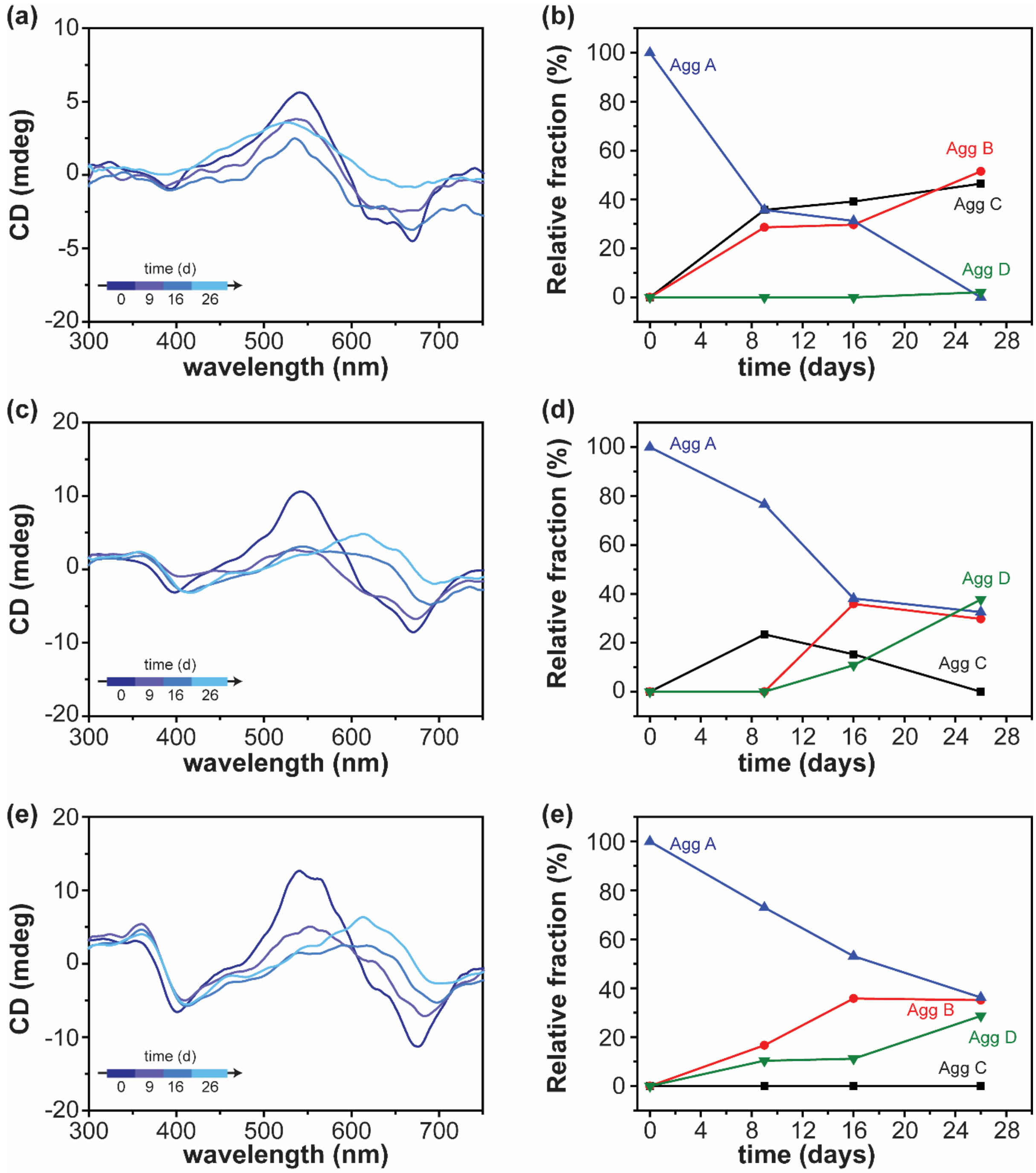

Figure for Reviewer. Time dependent kinetic experiment of Agg A performed in 10% DCE/MCH at different concentrations a) 5 µM, c) 10 µM, d) 20 µM, and the estimation of relative fraction (%) by deconvolution of CD spectra for b) 5 µM, d) 10 µM, e) 20 µM

Page 8: the authors speculate that "Agg D is a superhelix of Agg C, since the latter is accompanied with a chirality inversion". I do not understand the rational. One could state with the same reasoning from the CD data that C is a superhelix of B…

Answer: We agree with the reviewer that this point was not clearly supported in the discussion. It is true that the rational applied in this statement can be applied to **Agg B**, however, the distinct spectroscopic and microscopic data provided in the text demonstrate that **Agg B** and **Agg C** are aggregates of the same level or organization, whereas **Agg D** is clearly an entity of bigger dimensions. Still, as the reviewer pointed out, the statement "**Agg D** is a superhelix of **Agg C**, since the latter is

accompanied with a chirality inversion" is not so evident and thus, hypothetical. Nevertheless, in most of the helical supramolecular polymers which evolve into superhelices or hierarchically organized chiral structures described to date there is an inversion of chirality (see recent examples e.g., *Angew. Chem. Int. Ed*. **2017**, *56*,12634 and *Angew. Chem. Int. Ed*. **2019**, *58*, 1799). This type of helix to superhelix transition has been ascribed to the relaxation of helical stress exerted by the primary supramolecular helices by twisting around themselves (*Angew. Chem. Int. Ed.* **2007**, *46*, 5873), but the observed chirality inversion has not been successfully explained in these artificial systems. In contrast, this type of behavior has also been observed and successfully elucidated in the supercoiling of DNA.

We have deliberately included this discussion in the main text on pages 11-12 (highlighted in yellow): “The inversion of chirality observed during the transition from helices to superhelices may appear counterintuitive at first. However, such behavior is in fact common in hierarchical chiral assemblies. In many supramolecular polymers that evolve from primary helices into superhelical bundles or higher-order chiral structures, an inversion of handedness between the two hierarchical levels has been reported.[16–18] This phenomenon has generally been rationalized as a consequence of the relaxation of torsional stress present in the primary supramolecular helices, which can be relieved when the fibers coil around each other.[16] Nevertheless, the origin of the resulting chirality inversion has rarely been fully clarified in artificial supramolecular systems.

In contrast, analogous behaviour has been extensively studied in the context of DNA supercoiling.[57] In such systems, twisting of a primary helix generates supercoils that often adopt the opposite handedness because this configuration more efficiently relaxes torsional strain. Although supercoiling can in principle occur in either direction, opposite-handed coiling typically provides more effective stress relaxation and is therefore energetically favoured. Considering the distinct level of organization in the self-assembly of **Agg C** and **Agg D** and the seeding experiments results, it is reasonable to propose that **Agg D** is a superhelical structure formed through secondary nucleation of **Agg C**. The conditions that produce **Agg D** (i.e., supersaturation combined with slow cooling) consistent with hierarchical growth processes that favour secondary nucleation and subsequent fiber bundling. Similarly, the formation of **Agg A** under rapid solvophobic quenching conditions suggests that this superstructure may originate from secondary nucleation processes involving **Agg B** and **Agg C**. Further support for this hypothesis comes from concentration-dependent experiments: when the preparation protocol used for **Agg D** is applied at lower concentrations (3.5 μM), **Agg C** is initially formed and subsequently evolves partially into **Agg D**. Together, these observations support a hierarchical pathway in which helical aggregates (**Agg B/C**) act as precursors for the formation of superhelical assemblies (**Agg A/D**).”

Fig. S14: The figure caption is unclear. Was the cooling curve measured before or after the heating curve? Moreover, the phrasing "Changes in the CD signal at 658 nm of Agg D upon cooling " is confusing because Agg D is obtained at the lowest temperature and is therefore certainly not cooled. It should be "Changes in the CD signal at 658 nm of monomer QRD upon cooling" or "Changes in the CD signal at 658 nm of Agg D upon heating ". Finally, the solvent composition should be mentioned.

Answer: We apologize for the misleading caption. Due to the changes done in the manuscript, figure S14 has been eliminated and a more complete figure has been added in the revised SI version (new Figure S18).

Reviewer #2 (Remarks to the Author):

Hermans, Ruiz-Carretero, et al. reported on the pathway complexity involved in the self-assembly of a quinquethiophene-rhodanine (QRD) derivative in organic media, resulting in four distinct aggregate forms, each exhibiting unique morphologies and optoelectronic properties. In a mixed DCE/MCH solvent system, varying the preparation procedures enabled the isolation of aggregates Agg A through Agg D, which were characterized using UV-Vis and CD spectroscopy. Furthermore, the stability and interconversion of these self-assembled forms were investigated, with the relative proportions of each form determined by deconvolution of the CD spectra. Notably, the addition of seeds effectively accelerated the conversion of on-pathway species to the superhelical Agg D form, while a combination of thermal and sonication treatments could induce the disassembly of Agg D into Agg B and Agg C. While this manuscript demonstrates the complexity and interrelationships among different self-assembly pathways, the novelty and significance of these supramolecular systems remain unsubstantiated. Additionally, the characterization presented (and thus the overall content) is insufficient. In light of these concerns, the manuscript is not suitable for publication in Nature Communications and it is recommended to transfer to a specific journal e.g. Commun. Chem.

The following comments are suggested for consideration:
1. The concepts of pathway complexity and seeded supramolecular polymerization have been extensively developed over the past decade. What advances and significance does the supramolecular system described in this article offer compared to existing systems and methodologies? The authors are strongly encouraged to reorganize the introduction to clearly highlight the novelty and unique contributions of their work.

Answer: We thank the referee for the insightful comment. The introduction has been substantially revised and reorganized to more clearly highlight the conceptual advances and novelty of the present work, which we have updated and completed thanks to the questions raised by the referees.

In the previous version, the introduction primarily discussed supramolecular helicity and pathway complexity in a broad context, with emphasis on examples of helical structures in natural systems and the general importance of chirality in supramolecular assemblies. In the revised manuscript, we reposition the study within the current framework of pathway complexity and seeded supramolecular polymerization, explicitly outlining the unique aspects of the QRD system and how it advances beyond previously reported systems. In particular, the new introduction emphasizes: (i) the rare experimental observation and isolation of four distinct supramolecular aggregates originating from a single molecular building block, (ii) the quantitative reconstruction of the energetic landscape of these assemblies through calorimetric measurements, enabling direct comparison of their thermodynamic stability, (iii) the existence of interconnected on-pathway and off-pathway relationships between aggregates with different hierarchical organizations, and (iv) the central role of secondary nucleation processes in enabling the transformation of primary helical aggregates into higher-order superhelical structures. By explicitly introducing these aspects, the revised Introduction clarifies how the QRD system provides mechanistic insight into pathway selection, hierarchical organization, and secondary nucleation in supramolecular polymerization. Importantly, this combination of multiple experimentally isolatable states, quantitative thermodynamic analysis, and secondary nucleation–driven hierarchical transitions provides a level of mechanistic resolution that remains uncommon in supramolecular polymer systems. These revisions therefore better highlight the broader significance of our work for understanding and controlling complex supramolecular energy landscapes.

Additionally, we have changed the title of the manuscript from "**Pathway selection into 4 isolatable helical supramolecular aggregates"** to "**From Helices to Superhelices: Hierarchical Assembly across Competing Pathways".**

We include here the new text for the revised version of the introduction.

New Introduction: Helical organization is a fundamental structural motif in Nature, where molecular chirality is amplified across length scales to generate functional architectures such as the α-helix in proteins and the DNA double helix.[1,2] In synthetic supramolecular systems, hierarchical helicity likewise gives rise to emergent properties including circularly polarized luminescence (CPL),[3,4] chiral charge transport,[5] and chiral-induced spin selectivity (CISS).[4,6,7] A central objective in supramolecular chemistry is therefore to understand and control how molecular chirality propagates through self-assembly into higher-order structures.[8]

Over the past decade, it has become clear that supramolecular polymerization is inherently pathway-dependent.[9] Competing kinetic and thermodynamic routes can give rise to distinct aggregates from the same molecular building block, and significant progress has been made in identifying the molecular designs and experimental conditions required to control these pathways. While pathway complexity is now widely recognized, experimental access to multiple, well-defined and stable supramolecular states is still limited. In most systems, alternative assemblies are transient, partially overlapping, or only accessible through chemical modification, photoisomerization, or drastic solvent changes.[10–12] Consequently, supramolecular energy landscapes are often inferred indirectly, and mechanistic relationships between competing aggregates particularly across different hierarchical levels, remain difficult to resolve.

Chiral supramolecular systems[13,14] offer unique opportunities to expose hidden assembly pathways,[15] as excitonic coupling and chiroptical signatures provide sensitive structural reporters. Helix-to-superhelix transitions have been also observed, frequently accompanied by inversion of supramolecular chirality in both biological and synthetic contexts.[16–18] However, isolating more than two long-lived chiral supramolecular aggregates from a single enantiopure monomer under identical chemical conditions remains exceptionally rare.[19–21] One promising strategy to expand the number of accessible states is to combine pathway complexity with hierarchy in the self-assembly process. In this context, experimental tools that connect assemblies of different dimensionality such as cross-seeding processes remain scarce,[22–24] and the controlled use of secondary nucleation to generate hierarchical chiral superstructures is still in its infancy.[25–29]

Here we report a chiral π-conjugated quinquethiophene–rhodanine derivative (**QRD**, Figure 1a) that, through controlled thermal and mechanical protocols, gives access to four distinct and isolatable helical supramolecular aggregates (**Agg A–D**) from a single enantiopure monomer. Each aggregate exhibits a unique combination of chiroptical signature, morphology, and hierarchical organization, yet all are formed in the same solvent environment without chemical modification. The long-lived nature of these states enables direct calorimetric determination of their assembly enthalpies, allowing construction of a quantitative enthalpic energy landscape.

By integrating spectroscopy, concentration-dependent studies, cross-seeding experiments, and thermal/mechanical pathway switching, we experimentally resolve the relationships between these aggregates. We identify kinetically trapped hierarchical states, on-pathway interconversions between one-dimensional helices, and helix-to-superhelix transitions governed by concentration-dependent secondary nucleation. Remarkably, cross-seeding accelerates transformations between assemblies of different dimensionality, revealing an unusually interconnected energy landscape.

Together, these findings demonstrate that pathway selection alone can program supramolecular chirality and hierarchy, enabling four isolatable chiral structures to emerge from a single molecular building block. Beyond establishing a unique example of mechanistically resolved supramolecular polymorphism, this work provides general principles for mapping and manipulating complex energy landscapes in chiral self-assembly.

2. In this manuscript, the authors primarily investigate the assemblies using CD spectroscopy. While this allows differentiation of the pathways, the manuscript lacks a comprehensive understanding of the underlying self-assembly processes. Specifically, how are the molecules

arranged within each type of assembly? Since molecular packing has a profound impact on assembly morphology, it should be characterized in greater detail. Furthermore, given the observed chirality amplification and inversion, the packing modes in both the helix and superhelix forms warrant thorough discussion.

Answer: We thank the reviewer for this valuable comment. It is certain that CD spectroscopy and microscopy by themselves do not provide further information on the distinct molecular packings between the different aggregates. However, as discussed below in further detail, all our attempts to shed light on this point have resulted unfruitful.

Regarding the helix to superhelix transition, this phenomenon has been ascribed in other supramolecular polymers to the relaxation of helical stress exerted by the primary supramolecular helices by twisting around themselves (*Angew. Chem. Int. Ed.* **2007**, *46*, 5873). Of course, this phenomenon must also be accompanied by certain changes in molecular packing, but we consider that the contribution of fibre to fibre changes must be more substantial.

We have deliberately included this discussion in the main text on pages 11-12 (highlighted in yellow): "The inversion of chirality in a helix to superhelix transition does, in principle, not result so evident. In most of the helical supramolecular polymers which evolve into superhelices or hierarchically organized chiral structures described to date, there is inversion of chirality. [16–18] Helix to superhelix transition has been explained due to the relaxation of helical stress exerted by the primary supramolecular helices by twisting around themselves,[16] but the chirality inversion observed has not been successfully explained in artificial systems. In contrast, this type of behavior has also been observed and successfully elucidated in the supercoiling of DNA.[57] Even though twisting or superhelix formation can proceed in both senses, the twisting is more efficient and more abundant when there is chirality inversion (in this case, to yield left-handed coiling) due to the better stress relaxation of the resulting helical structure (*i.e.*, it attends to energetic reasons).Considering the distinct level of organization in the self-assembly of **Agg C** and **Agg D** and the seeding experiments results, it is judicious to think that **Agg D** is a superhelix obtained through secondary nucleation of **Agg C**. The conditions of formation of **Agg D** (with supersaturation and slow cooling rates) and those of **Agg A** (supersaturation through solvophobic quenching) make reasonable to think that these superstructures are formed through secondary nucleation of **Agg B** and **Agg C**".

3. Building on the previous point, the reviewer notes that the amide groups in the alanine motifs likely play a crucial role in chirality transfer and pathway determination. Additional techniques such as NMR and IR spectroscopy should be employed to characterize the hydrogen bonding interactions. How does the packing of hydrogen bonds correlate with the different assembly pathways?

Answer: We acknowledge the reviewer for addressing this point. As the reviewer points out, the distinct strength and nature of intermolecular H-bonds between amide bonds as well as intermolecular H-bonds between amides and other groups susceptible of this interaction have been utilized to direct pathway assemblies, and it is rational to think that they also play an important role in our system. We initially attempted to shed light on this topic through NMR experiments. Unfortunately, the low solubility of the aggregates at higher concentrations and the broad bands observed made it difficult to gain information by experiments like ROESY/NOESY, concentration dependent and/or variable temperature (VT)-NMR. We have additionally performed FTIR experiments in solution to obtain information about the strength of the H bonds in the distinct aggregates (Figure for Reviewer 2.1. below). However, the differences between the amide I and amide II bands of these aggregates are minimal, unravelling the negligible differences concerning the H-bonding pattern and strength in these aggregates. We

hypothesize that **Agg B** and **Agg C** show a very similar internal order, which only differs in the helicity of the resulting self-assembled monodimensional structures. The transfer of asymmetry, due to remoteness of the chiral center to the π-conjugated system, is not so efficient as in referable supramolecular systems and it leads to two aggregates of opposite helicity which are still close in energy. **Agg A** and **Agg D** are obtained through the secondary nucleation of **Agg B** and **Agg C**, respectively, in conditions which favor supersaturation (direct injection of monomer and slow cooling at high concentrations) and given the fact that **Agg B** and **Agg C** gain stability upon supercoiling and helical inversion, as discussed briefly before and more in detail in the current version of the manuscript.

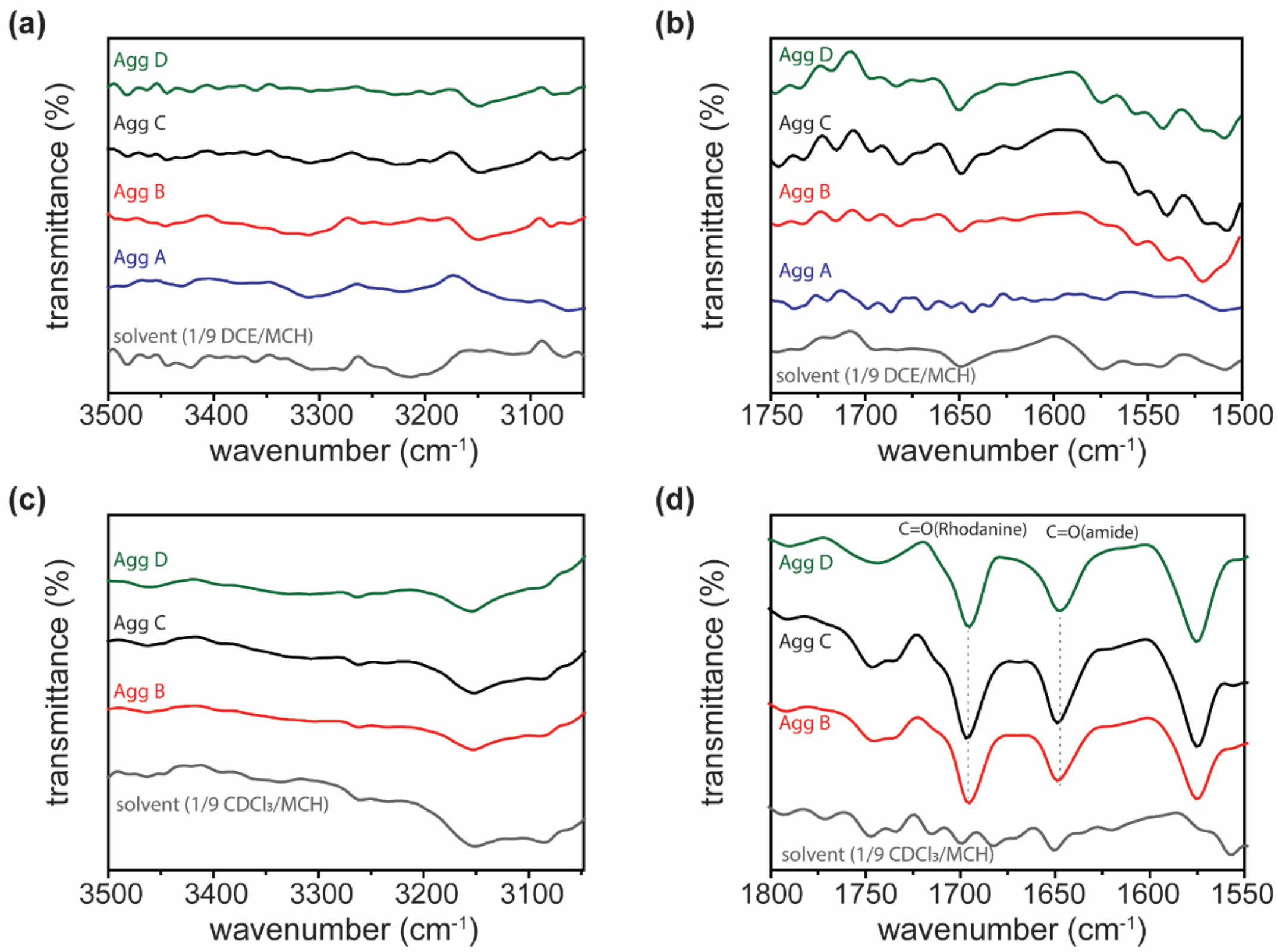

Figure for Reviewer 2.1. Partial FT-IR spectra of **Agg A-D** prepared in 1/9 DCE/MCH (20 μM); a) N-H region and b) C=O region, partial FT-IR spectra of **Agg B-D** prepared in 1/9 $CHCl_3$/MCH (200 μM); c) N-H region and d) C=O region.

Additionally, we performed PXRD measurements on drop cast films deposited on glass substrates. **Agg A-D** were prepared following the described protocols in section 2 of the SI (20 mM, 10% DCE/MCH). As it can be seen in the following Figure for Reviewer 2.2, the results obtained do not provide useful information due to the lack of defined peaks.

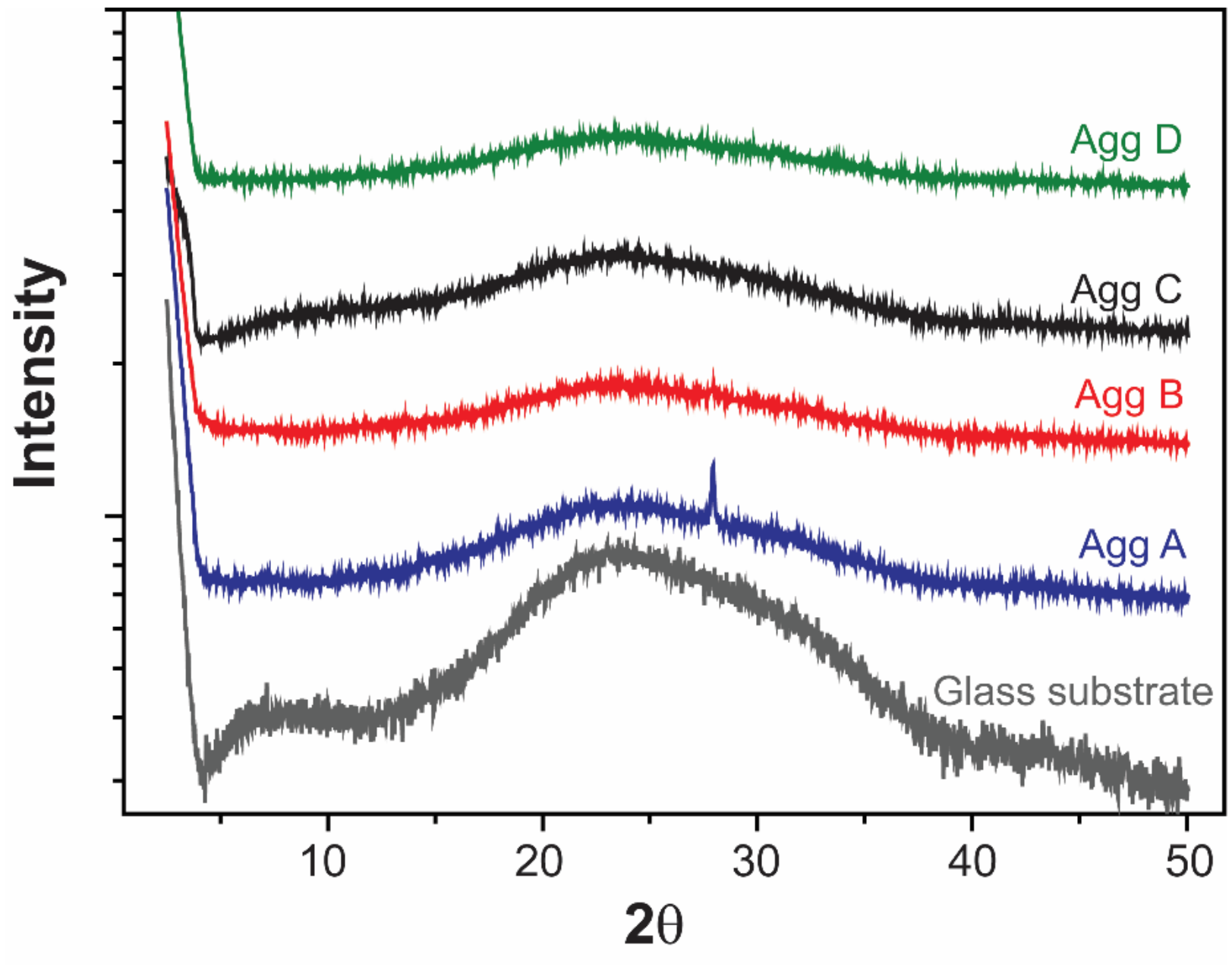

Figure for Reviewer 2.2. PXRD spectra of **Agg A-D** prepared onto glass substrate.

We have attempted as well to obtain structural data from Small Angel X-Ray Scattering (SAXS) and cryogenic Transmission Electron Microscopy (cryo TEM). The raw scattering curves of each of the aggregates liquid phases (Figure for Reviewer below), are similar to that of the solvent. Thus, do not exhibit feature characteristics of nanostructures that can by further analyzed to obtain size and shape. These results are supported by cryo-TEM images of aqueous solution of **Agg B** and **Agg C** (following Figure for Reviewer 2.4) in which no distinct structure or specific ordering can be observed. Although there are some bundles in the images of **Agg B** but the contrast is too low to extract additional information.

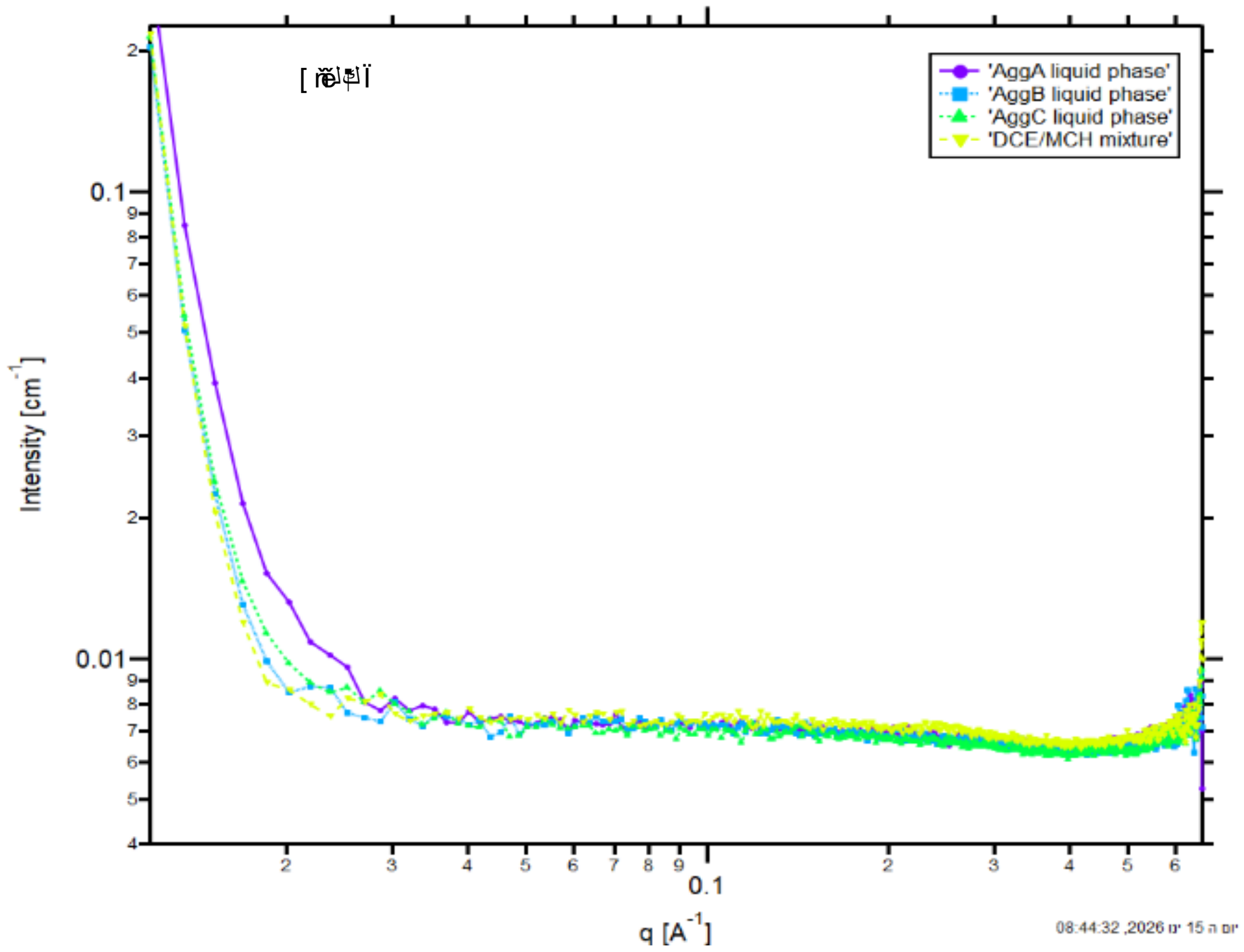

Figure for Reviewer 2.3: SAXS curves of the liquid phase of aggregates A-C and the solvent DCE/MCH (1:9, v/v) mixture.

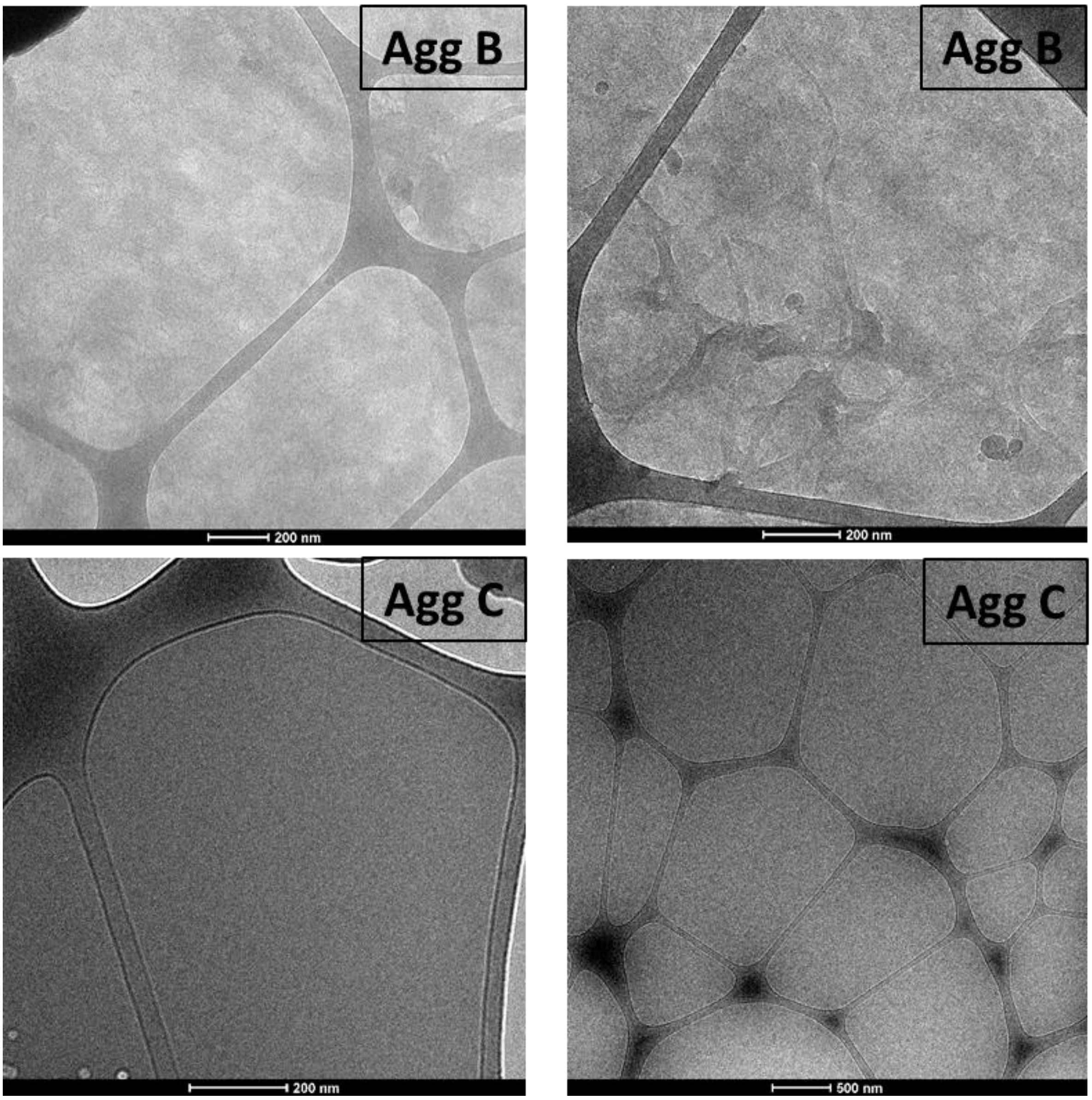

Figure for Reviewer 2.4: Cryo-TEM images of aqueous solution of **Agg B** (top row) and **Agg C** (bottom row)

4. The authors utilized distinct preparation methods to obtain different aggregates. What can be obtained if fast cooling was performed and without sonication (as analog of method for Agg D)? For Agg B, the procedure of sonication (in room temperature bath) of high temperature solutions is unusual. What influence can this cause comparing to the two cooling processes (fast and slow cooling)?

Answer: We also compared CD spectra without sonication during the formation processes of **Agg A** and **Agg C**. Before applying sonication, the obtained CD spectra were similar to the spectra obtained after applying sonication (See Figure for Reviewer below). By performing the sonication, the solubility of the aggregates increased as well as helping the organization of the aggregates.

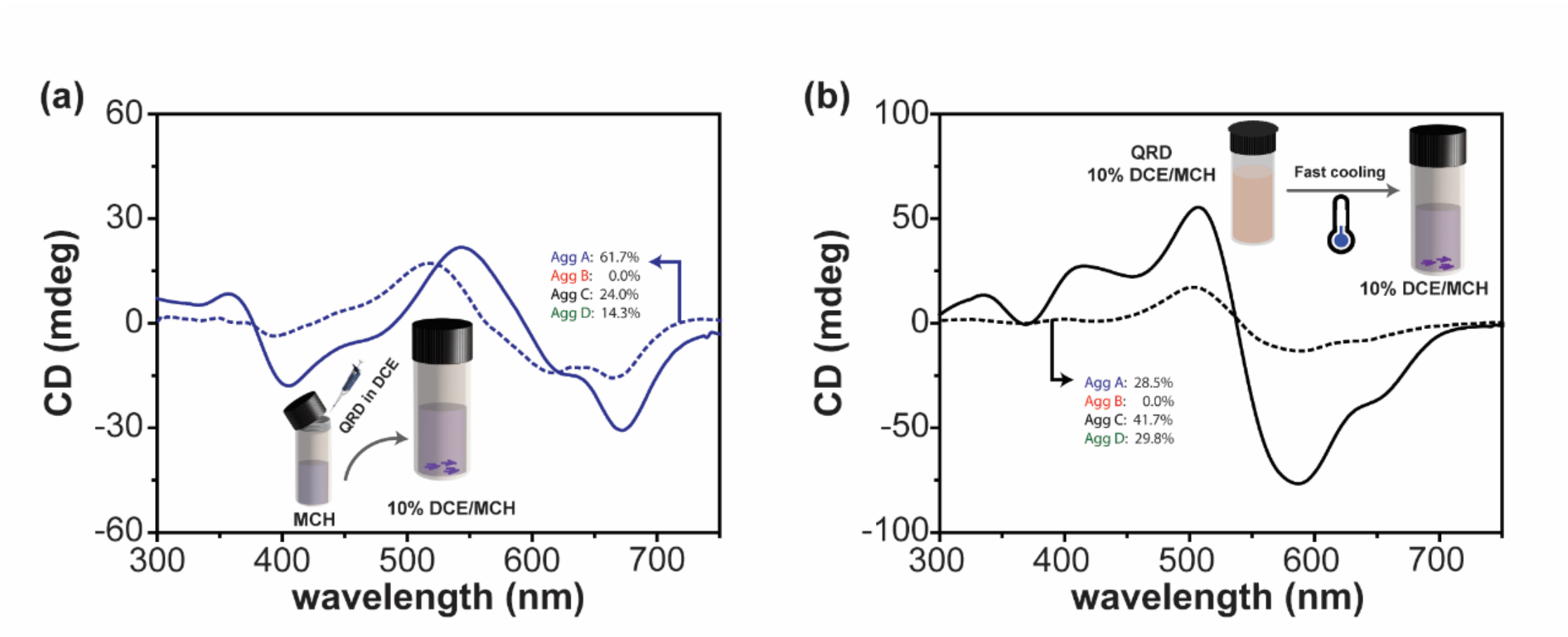

Figure for Reviewer. a) CD spectrum of **QRD** (20 µM, 10% DCE/MCH) by injection of **QRD** diluted in DCE into MCH without further treatment (i.e. Sonication, dotted blue line), b) CD spectrum of **QRD** (20 µM, 10% DCE/MCH) after heating (monomeric state) and fast cooling in an ice bath without further treatment (i.e. Sonication, dotted black line). The original **Agg A** and **Agg C** spectra (with sonication, solid blue and black lines) are added for comparison.

We agree that the protocol of formation of **Agg B** is not very common. With this protocol we favor alternative nucleation pathways that can lead to states which are not attained through more usual cooling rate variation protocol. In our case, both fast (10 K min^{-1}) and slow (1K min^{-1} or below) yielded **Agg D** at the original concentrations explored. As discussed in the current version of the manuscript in the formation of **Agg D** secondary nucleation plays a role, which explains why that is the main product formed with this usual protocol (aside from its higher thermodynamic stability). Tuning of concentration and application of a slow cooling rate only provides **Agg C**. In the end, with sonication at higher temperatures the kinetic barrier through the monomer from **Agg C** is overcome, which lays out the usefulness of this novel preparation method.

5. In Figures 2a and 2b, why does the CD signal around 600 nm increase from day 3 to day 7, even though the fraction of Agg B is at its lowest on day 7? Please clarify this apparent discrepancy between the CD signal intensity and the aggregate composition.

Answer: We attribute this small discrepancy to experimental errors within the measurement. When following the spontaneous conversion of **Agg B** to **Agg D**, we found roughly the same conversion percentage at the three concentrations tested.

6. It is recommended that the cross-on-dot symbols in Figures 2b (15 µM concentration) and 2d (20 µM concentration) be modified, as they are difficult to distinguish from solid dots. Please consider using more distinct markers to improve clarity.

Answer: We have modified the symbols and line to be more distinguishable in new Figure 2. Since we have incorporated additional data including **Agg A**, panels b and d of Figure 2, are now panels c and f.

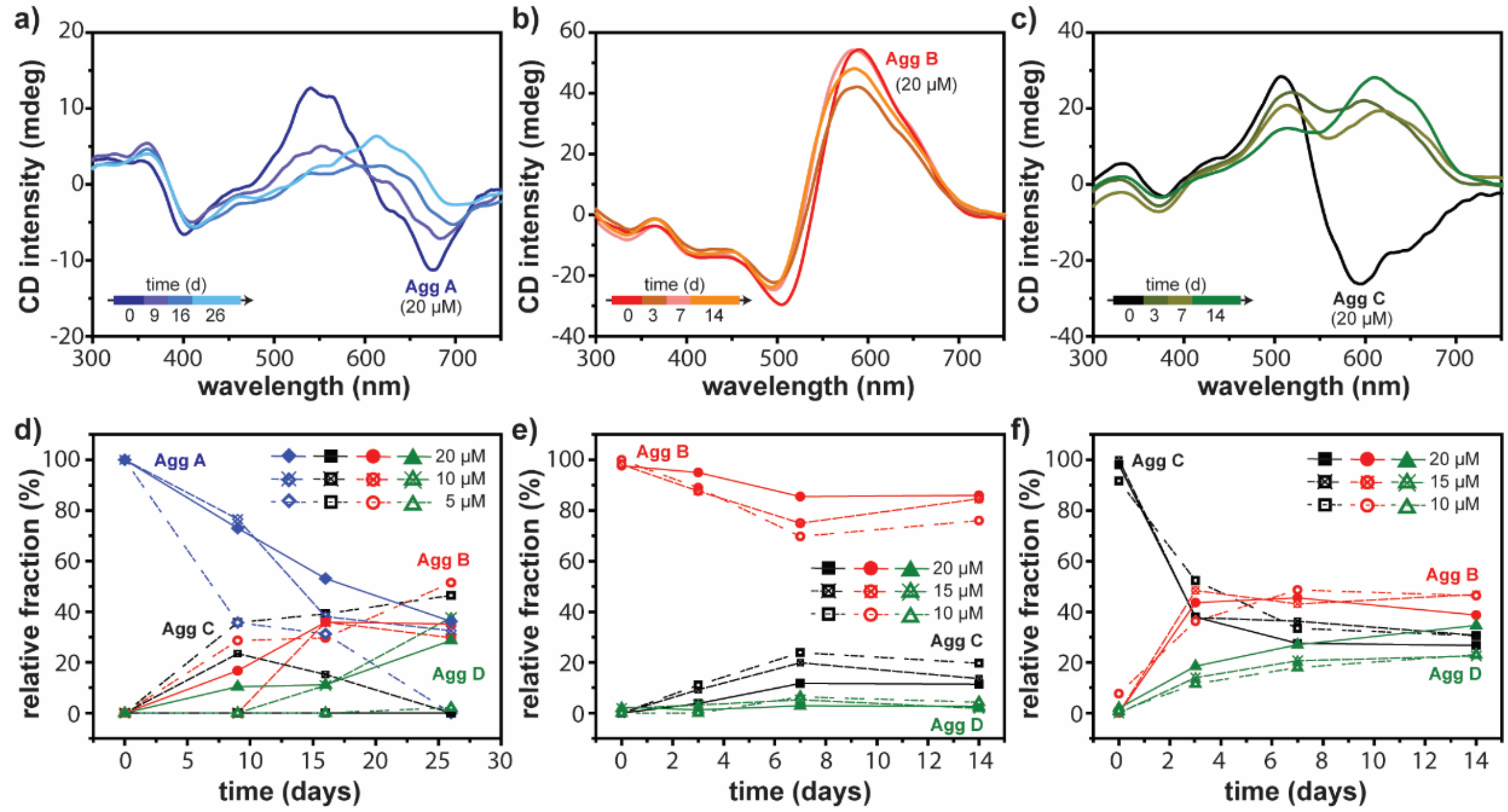

New Figure 2. On-pathway spontaneous conversions. CD spectra of a) **Agg B** (20 µM, 1/9 DCE/MCH) and c) **Agg C** (20 µM, 1/9 DCE/MCH) over the course of 14 days at room temperature. The relative aggregate fractions obtained by spectral deconvolution (see Supporting Information section 5) at different concentrations for b) **Agg B** and d) **Agg C**.

7. On page 8, the manuscript states, "Agg A should be a superhelix of Agg B, but cannot be formed from it since it is energetically uphill." Is there any evidence supporting this argument? If Agg A is directly related to Agg B as its superhelix form, why does the energy of Agg A differ so significantly compared to Agg D as the superhelix of Agg C?

Answer: We thank the reviewer for the valuable comment, since it also arose during the preparation of the manuscript. **Agg A** is formed under conditions which highly favor kinetic trapping and secondary nucleation (injection of the monomer in a good solvent into the bad solvent at relatively high concentration). New experiments of the evolution of **Agg A** with time show that the main product formed is **Agg B**, which could result from the debundling of **Agg A** into **Agg B** due to energetic reasons (see New Figure 2a, 2d above). The differences in energy between **Agg D** and **Agg A** account for the formation of the "wrong" superhelix, as dictated by the intrinsic chirality of QRD.

8. The manuscript claims, "Agg B—unable to convert to Agg D—acts as a monomer reservoir, accelerating Agg C conversion into superhelix Agg D." Is there any evidence supporting this process, particularly the disassembly of Agg B? The deconvolution analysis was only performed for equilibrium solutions; please provide additional data or discussion to substantiate this claim.

Answer: It is true that this point does not result as evident, especially when compared to the reported examples in the literature or on-pathway and off-pathway mechanisms between aggregates. With all the evidence we have now, it is more likely that **Agg B** is off-pathway towards **Agg C**. The slow and not very efficient evolution with time of **Agg B** towards **Agg C**, as well as the fact that this conversion is even slower at higher concentrations, would agree with **Agg B** being an almost kinetically- trapped, off pathway species towards **Agg C**. Furthermore, and substantiated by the inversion of chirality seen in the literature between helices and superhelices, it makes sense that **Agg B** is unable to form **Agg D** by itself. Finally, the formation of **Agg B** from **Agg D** in the sonication experiments at 25 ºC make feasible to think that the combination of temperature and ultrasonication permit to overcome the barrier between

Agg B and **Agg C** via monomer easily. All in all, although we do not have evidence from temperature or concentration dependance behavior of this transition that further support this claim, we consider that this sum of data makes reasonable to think than the conversion of **Agg B** to **Agg D** must be via monomer (and towards **Agg C**) and hence, in the seeding experiments **Agg B** can be considered as a monomer reservoir towards the formation of **Agg C**.

9. Minor Typographical Errors

• Page 5: In the sentence "… confocal microscopy (Figure S2) Indeed, …" a period is missing after the parenthesis.

• Page 9: In the sentence "Sonication at 10 °C for 30 min. provoked the conversion …" there is an unnecessary period after "min".

Answer: We apologize for the typographical errors. They have been corrected in the main text and the changes highlighted in yellow, now in pages 9 and 12 in the revised version.

Reviewer #3 (Remarks to the Author):

Title: Pathway selection into 4 isolatable helical supramolecular aggregates

This manuscript describes the self-assembly of a π-conjugated chiral quinquethiophene–rhodanine system driven by π–π stacking and hydrogen bonding, resulting in a complex energy landscape. By employing four preparation protocols involving mechanical and thermal perturbations, the authors report the formation and isolation of four chiral supramolecular aggregates with distinct chiroptical, hierarchical, and morphological features. Measurements of assembly enthalpies by isothermal titration calorimetry, together with spontaneous, induced, and seeded interconversion experiments, are used to construct an energy landscape comprising on- and off-pathway relationships among the aggregates. The results have the potential to advance the area of chiral supramolecular materials. However, the manuscript is not suitable for publication in Nature Communications in its current form, as several issues need to be addressed to substantiate the mechanistic claims and improve clarity.

The seeding experiments are an important part of the interpretation of the energy landscape. Given the metastability and interconvertibility of the aggregates, the preparation, purity, and stability of the seeds are crucial points. However, the manuscript does not describe the preparation protocol and characterization of the seeds. The authors should provide the seed preparation protocol and include AFM images, size distributions, and time-dependent CD data of the seed solutions to ensure their purity and stability over the timescales relevant to the experiments.

Answer: We thank the reviewer for this valuable input. In fact, our seeds are the real isolated aggregates, not the conceptual "seeds" usually found in the current literature of seeding supramolecular polymerization. This accounts for the fact that sonication plays an important role in the preparation of our aggregates, which restricts the formal preparation of seeds in analogy to the reported seeds in our system. The lengths of **Agg B** and **Agg C** are around 600 nm and 300 nm, respectively, which exceed approximately, in an order of magnitude the size of those usually reported (in the first reports of seeded supramolecular polymerization, with representative examples like *Nat. Chem*. **2014**, 6, 188, *J. Am. Chem. Soc.* **2015**, 137, 3300, *J. Am. Chem. Soc*. **2016**, 138, 670 or *Angew. Chem. Int. Ed*. **2018**, 57, 2339 to cite a few, the seed lengths are around 30-100 nm, whereas in recent manuscripts like *J. Am. Chem. Soc*. **2022**, *144*, 22479, *Chem. Commun*, **2023**, *59*, 7375 or *Nat Commun*. **2024**, *15*, 3672, among many others, these values can be even lower, with values between 10-90 nm). Other examples found in literature, such as *J. Am. Chem. Soc*. **2022**, 144, 11306 and *Angew. Chem. Int. Ed*. **2025**, *64*, e202413747 show secondary nucleation processes using seeds of 800 nm in size approximately, which is in line with our results. Still, since here we do not intend to control growth but to understand the transition between aggregates, we think that this point is not crucial in our system. Actually, these sizes can also help to trigger secondary nucleation events, which, as discussed lately, we demonstrate is paramount in the formation of **Agg D**.

Furthermore, we have demonstrated that our "seeds" behave like the usually found seeds in literature and therefore, we had referred to them as seeds, even though they are aggregates in reality.

To clarify this point, we have included this discussion in the first paragraph of the section "seeding experiments, secondary nucleation and thermal and mechanical treatment" on page 10 (highlighted in yellow): "In our system, given the effect that sonication exerts in the preparation of the different aggregates, the formation of seeds through ultrasonication, as usually reported in the literature, was not feasible. Thus, here we refer to "seeding" with the addition of aliquots of **Agg B-D** as prepared per the protocols explained above (See Section 6 of the SI for details). The lengths of **Agg B** and **Agg C** (around

300 nm and 600 nm, respectively) are higher than most of the seeds employed in the literature (30-100 nm). However, notable exceptions with longer lengths have also been utilized (> 800 nm)[48,49], which have actually helped triggering other assembly phenomena, such as secondary nucleation".

Additionally, a specific section dedicated to seeded experiments has been included in the revised version of the SI as section 6. The changes are highlighted in yellow in the revised version of the SI.

The text added in the revised SI is as follows:

6. Seeding conversion experiments

1) General experimental conditions

To perform the seeding conversion experiments, Agg B and Agg C solutions were prepared by following the protocols described in Section 2 of the supplementary information. As summarized in table S6, Agg B and Agg C were mixed at different volumetric ratios.

It should be noted that both Agg B and Agg C solutions were prepared solely according to the standard protocols, and no additional treatment was applied to generate seeds before it was mixed. This is because sufficient sonication had already been applied during their respective preparation procedures, ensuring the presence of seed structures in both Agg B and Agg C solutions.

After adding the "seeds" to the solution containing specific aggregates, the circular dichroism (CD) spectra of the resulting solutions were monitored after 24 hours to evaluate the seeding induced conversion behaviors.

Seed		0.5% (v/v)	1%	2%	5%	10%
Exp 1.	Agg C	12.5	25	50	125	250
	Agg B	2487.5	2475	2450	2375	2250
Exp 2.	Agg B	12.5	25	50	125	250
	Agg C	2487.5	2475	2450	2375	2250

Exp 1. is the seeding conversion experiment with varying amount of Agg C(seeds)

Exp 2. Is the seeding conversion experiment with varying amount of Agg B(seeds)

The following additional points should also be addressed properly to improve the quality of the manuscript further.

(i) The introduction section does not adequately convey the significance of the work to the readers. Given that the title emphasizes the achievement of "4 isolatable chiral aggregates", the introduction should clearly state the fundamental challenges associated with isolating multiple (4 here) chiral aggregates from a single monomer, explain why this has not been achieved previously, and clarify how the present work strategically overcomes these limitations, beyond empirical protocol variation. Addressing these points would substantially strengthen the motivation and framing of the study.

Answer: We thank the reviewer for the insightful comments about our work. We agree with the referee on this point and therefore, we have changed the introduction to clarify how our work overcomes the

limitations of achieving several chiral aggregates from a single monomer. Furthermore, after the suggestions and comments from the referees we have completed the aggregates interconversion processes, and we have added the data that demonstrate the formation of structures with higher levels of hierarchy (helices to superhelices) through secondary nucleation processes. Furthermore, we have also changed the title from "**Pathway selection into 4 isolatable helical supramolecular aggregates"** to "**From Helices to Superhelices: Hierarchical Assembly across Competing Pathways"** to clarify better the novelty of the work.

In the previous version, the introduction primarily discussed supramolecular helicity and pathway complexity in a broad context, with emphasis on examples of helical structures in natural systems and the general importance of chirality in supramolecular assemblies. In the revised manuscript, we reposition the study within the current framework of pathway complexity and seeded supramolecular polymerization, explicitly outlining the unique aspects of the QRD system and how it advances beyond previously reported systems. In particular, the new introduction emphasizes: (i) the rare experimental observation and isolation of four distinct supramolecular aggregates originating from a single molecular building block, (ii) the quantitative reconstruction of the energetic landscape of these assemblies through calorimetric measurements, enabling direct comparison of their thermodynamic stability, (iii) the existence of interconnected on-pathway and off-pathway relationships between aggregates with different hierarchical organizations, and (iv) the central role of secondary nucleation processes in enabling the transformation of primary helical aggregates into higher-order superhelical structures. By explicitly introducing these aspects, the revised Introduction clarifies how the QRD system provides mechanistic insight into pathway selection, hierarchical organization, and secondary nucleation in supramolecular polymerization. Importantly, this combination of multiple experimentally isolatable states, quantitative thermodynamic analysis, and secondary nucleation–driven hierarchical transitions provides a level of mechanistic resolution that remains uncommon in supramolecular polymer systems. These revisions therefore better highlight the broader significance of our work for understanding and controlling complex supramolecular energy landscapes.

We include here the new text for the revised version of the introduction.

New Introduction: Helical organization is a fundamental structural motif in Nature, where molecular chirality is amplified across length scales to generate functional architectures such as the α-helix in proteins and the DNA double helix.[1,2] In synthetic supramolecular systems, hierarchical helicity likewise gives rise to emergent properties including circularly polarized luminescence (CPL),[3,4] chiral charge transport,[5] and chiral-induced spin selectivity (CISS).[4,6,7] A central objective in supramolecular chemistry is therefore to understand and control how molecular chirality propagates through self-assembly into higher-order structures.[8]
Over the past decade, it has become clear that supramolecular polymerization is inherently pathway-dependent.[9] Competing kinetic and thermodynamic routes can give rise to distinct aggregates from the same molecular building block, and significant progress has been made in identifying the molecular designs and experimental conditions required to control these pathways. While pathway complexity is now widely recognized, experimental access to multiple, well-defined and stable supramolecular states is still limited. In most systems, alternative assemblies are transient, partially overlapping, or only accessible through chemical modification, photoisomerization, or drastic solvent changes.[10–12] Consequently, supramolecular energy landscapes are often inferred indirectly, and mechanistic relationships between competing aggregates particularly across different hierarchical levels, remain difficult to resolve.
Chiral supramolecular systems[13,14] offer unique opportunities to expose hidden assembly pathways,[15] as excitonic coupling and chiroptical signatures provide sensitive structural reporters. Helix-to-superhelix transitions have been also observed, frequently accompanied by inversion of supramolecular chirality in both biological and synthetic contexts.[16–18] However, isolating more than two long-lived chiral supramolecular aggregates from a single enantiopure monomer under identical chemical

conditions remains exceptionally rare.[19–21] One promising strategy to expand the number of accessible states is to combine pathway complexity with hierarchy in the self-assembly process. In this context, experimental tools that connect assemblies of different dimensionality such as cross-seeding processes remain scarce,[22–24] and the controlled use of secondary nucleation to generate hierarchical chiral superstructures is still in its infancy.[25–29]

Here we report a chiral π-conjugated quinquethiophene–rhodanine derivative (QRD, Figure 1a) that, through controlled thermal and mechanical protocols, gives access to four distinct and isolatable helical supramolecular aggregates (Agg A–D) from a single enantiopure monomer. Each aggregate exhibits a unique combination of chiroptical signature, morphology, and hierarchical organization, yet all are formed in the same solvent environment without chemical modification. The long-lived nature of these states enables direct calorimetric determination of their assembly enthalpies, allowing construction of a quantitative enthalpic energy landscape.

By integrating spectroscopy, concentration-dependent studies, cross-seeding experiments, and thermal/mechanical pathway switching, we experimentally resolve the relationships between these aggregates. We identify kinetically trapped hierarchical states, on-pathway interconversions between one-dimensional helices, and helix-to-superhelix transitions governed by concentration-dependent secondary nucleation. Remarkably, cross-seeding accelerates transformations between assemblies of different dimensionality, revealing an unusually interconnected energy landscape.

Together, these findings demonstrate that pathway selection alone can program supramolecular chirality and hierarchy, enabling four isolatable chiral structures to emerge from a single molecular building block. Beyond establishing a unique example of mechanistically resolved supramolecular polymorphism, this work provides general principles for mapping and manipulating complex energy landscapes in chiral self-assembly.

(ii) The AFM images provided lack sufficient resolution to clearly visualize the aggregates. Improved/higher-magnification AFM images should be included to enable a more reliable structural assessment. In addition, the analysis is currently limited to fiber heights and lengths of the aggregates. Given that all four isolatable aggregates are described as helical in nature, their helical pitch distribution should be included for comparison among the aggregates.

Answer: We have included additional AFM images of **Agg A-Agg D** (New Figures S7-S8) where the helical pitch distribution is shown. In this case, the samples were deposited by spin-coating at 1000 rpm at 10 μM (lower concentration than the images shown in the main text and SI currently) to achieve thinner layers and better imaging of the aggregates.

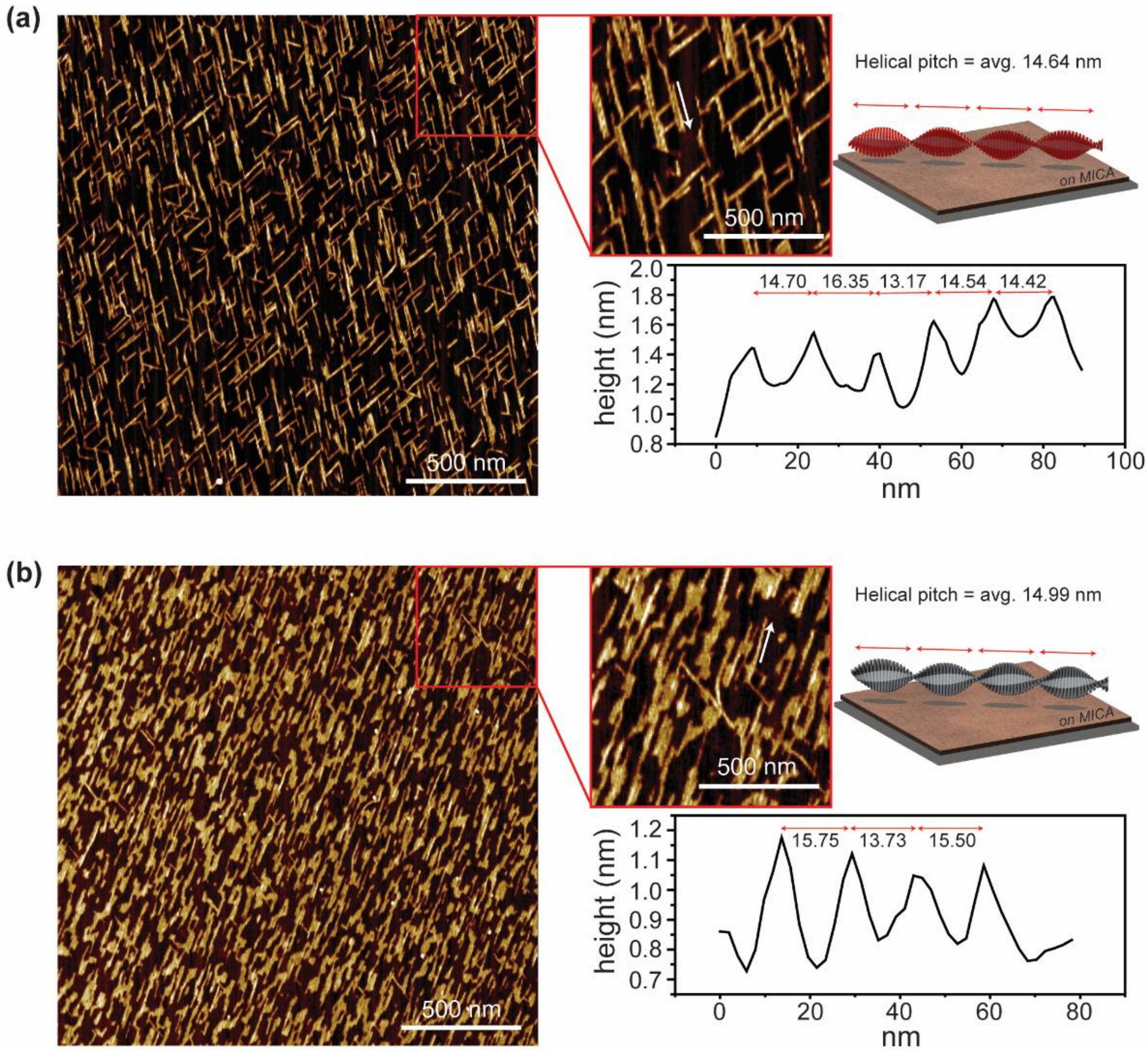

New Figure S7. AFM images and helical pitch analysis of a) **Agg B** and b) **Agg C** on samples deposited on mica substrates by spin coating 10 μM 1/9 DCE/MCH solutions at 1000 rpm for 1 minute.

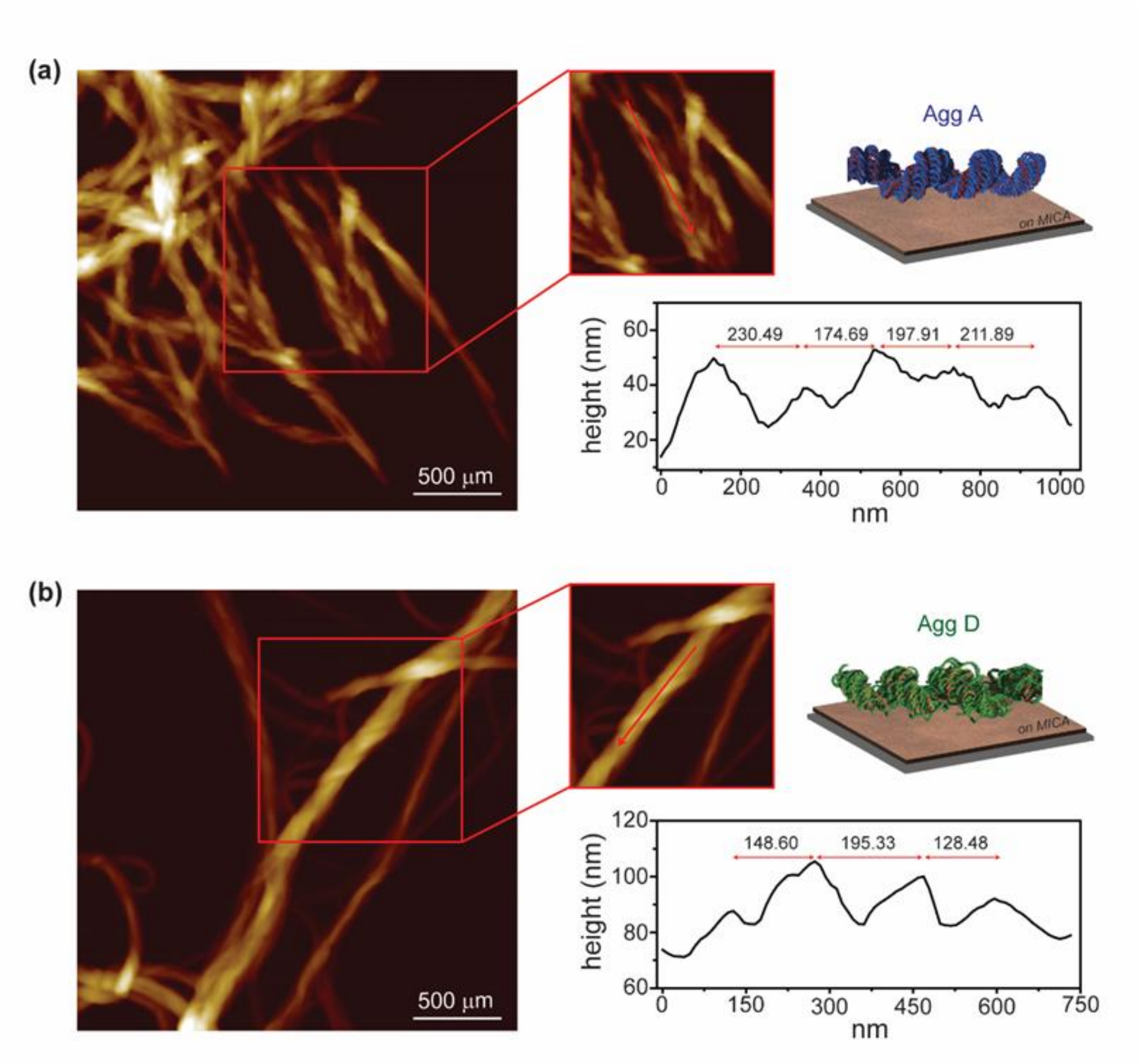

Figure S8 (new). AFM images and helical pitch analysis of a) **Agg A** and b) **Agg D** on samples deposited on mica substrates by spin coating 20 μM 1/9 DCE/MCH solutions at 1000 rpm for 1 minute.

(iii) During the preparation of the bundled aggregates (Agg A and Agg D), it remains unanswered when and how bundling occurs. Does bundling arise at the initial or later stage of assembly? The manuscript does not provide any evidence to clarify this mechanism. What kind of interactions mediate the association of individual helical fibers into bundled or superhelical structures? To confirm bundling as a hierarchical process, 1D and 2D X-ray diffraction experiments are recommended to probe whether there is any subtle difference in internal molecular packing between the helical and superhelical aggregates.

Answer: We acknowledge the reviewer for this valuable input, since it is certain that this point remained unanswered in the previous version of the manuscript. We have attempted to perform 1D and 2D X-ray diffraction of **Agg A-Agg D**. However, the results obtained are not conclusive and do not permit us to extract further conclusions (See Figure for Reviewer 3.1 below). However, a closer inspection of **Agg A** and **Agg D** formation has led us to conclude that in their formation (formally, via "bundling" or helix to helix interaction) there is a strong contribution of secondary nucleation events. **Agg A** is formed in conditions that strongly favor secondary nucleation, i.e., supersaturation conditions, since we directly inject the monomeric solution in a bad solvent (solvophobic quenching in supersaturation conditions). At the same time, **Agg D** is formed more efficiently when slow cooling rates (1K/min or less in this system) are applied, which further support this claim, in analogy to other reports (see *Chem. Eur. J* **2019**, *25*, 13008 or *Nat. Chem*. **2025**, *17*, 477 for a comprehensive review on the topic). What results

particularly enlightening is that at lower concentrations (i.e., less "supersaturated" regimes) and applying the same conditions of formation than for **Agg D**, **Agg C** is formed. (In fact, with time, **Agg C** in these conditions partially evolves to **Agg D** (with lag time of approximately 1 hour). Altogether, these observations suggest that, in the formation of **Agg A** and **Agg D** following the protocols described in the main text, the bundling of **Agg B** and **Agg C** occurs at the early steps of self-assembly and triggers superhelix formation of **Agg A** and **Agg D**, respectively, through secondary nucleation. However, this bundling can be observed at slower rates with time by controlling the concentration of the sample. Finally, the time evolution of **Agg B** to **Agg A** is not seen due to the higher thermodynamic stability of **Agg B** when compared to **Agg A**.

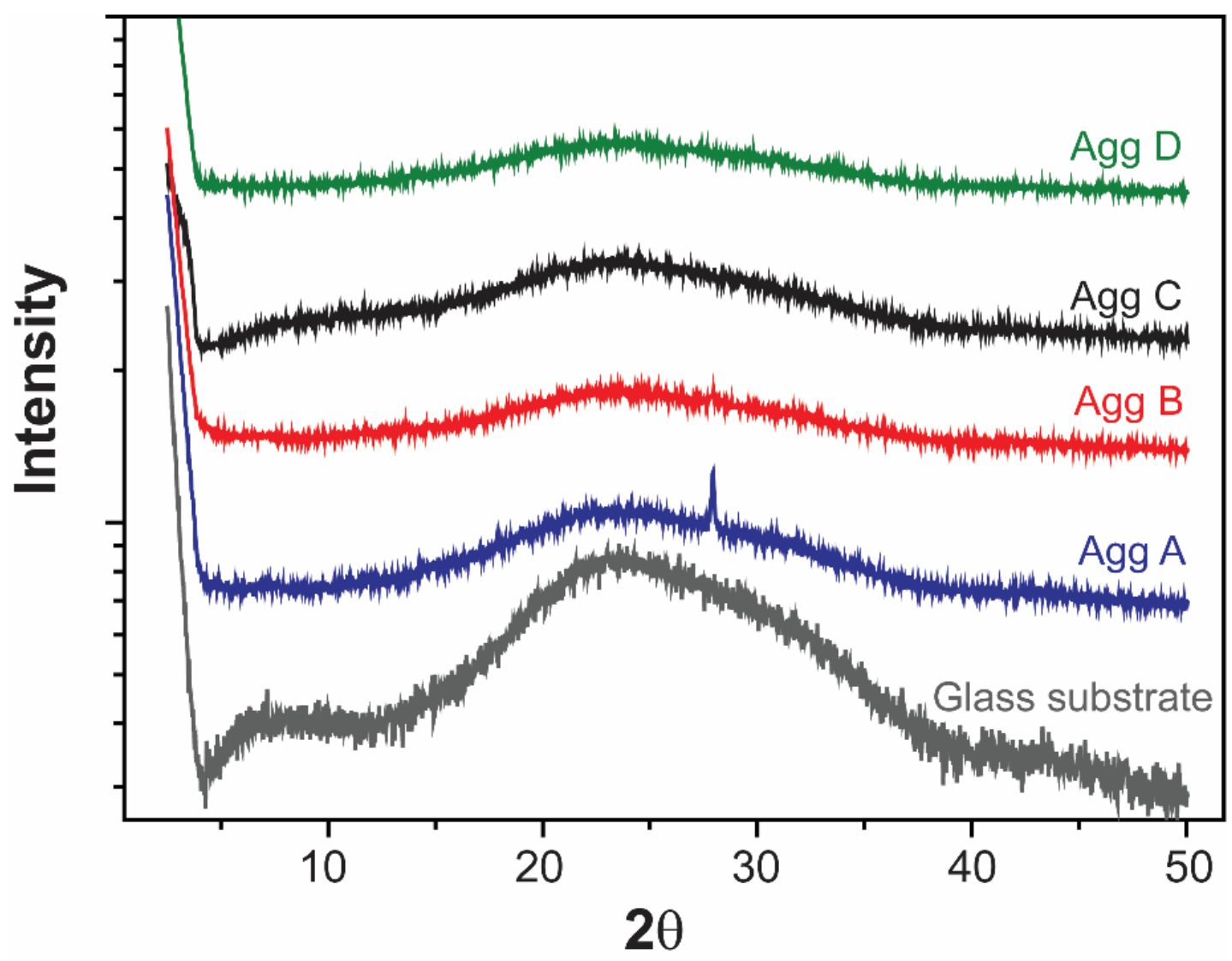

Figure for Reviewer 3.1. PXRD spectra of **Agg A-D** prepared onto glass substrate.

(iv) Under external perturbation—such as mechanical agitation or changes in solvent composition—do the bundled aggregates undergo stepwise debundling followed by molecular disassembly? In particular, the authors should clarify whether the conversion of Agg D into Agg B or Agg C under sonication proceeds via partial debundling (fragmentation) or via complete disassembly into monomers followed by reassembly. If disassembly into monomers is involved, why does kinetically trapped Agg A not form? To get insight into the formation of Agg A, it is essential to characterize the species formed in 1:9 dichloroethane/methylcyclohexane solution by injection at room temperature. This point is critical, as sonication of this solution produces Agg A, whereas all other aggregates are obtained starting from the monomeric solutions prepared at elevated temperature.

Answer: We thank the reviewer for this valuable comment. This point is not trivial, resulting difficult to prove. As shown in Figure 3f, the conversion of **Agg C** from **Agg D** with sonication and lower temperatures (5-10 ºC) corresponds to the direct debundling of **Agg D**, since **Agg C** is the main component of **Agg D**, and working at these temperatures ensures that no further evolution to the more kinetically trapped **Agg B** occurs. When the same debundling experiment is performed at higher temperatures (25-30 ºC), **Agg B** is also formed, and this must be via monomer from **Agg C** (i.e. **Agg B** is off-pathway towards **Agg C** despite being formally on-pathway to **Agg D**). If **Agg B** were on-pathway to **Agg C**, we would expect a more marked kinetic evolution of **Agg B** towards **Agg C** and that this transition happened easily at lower temperatures (conditions in which we only see **Agg C**). Despite we

form **Agg B** via monomer, we know that the formation of **Agg A** is obtained under conditions which seem to favour kinetically trapped states and secondary nucleation (injection of the monomer from good solvent into the bad solvent, i.e., solvophobic quenching) and, at the same time, is the less stable species of the energy landscape. As discussed further in point (iii) this difference in stability as well as the very particular supersaturation conditions required in the formation of **Agg A** make almost impossible to form it under these perturbation conditions.

(v) In Fig. 3c, Agg B (91%) exhibits lower CD intensity and different spectral features compared to Agg B (81%) in Fig. 3d, although the solutions have identical total concentrations. The origin of this discrepancy should be clarified.

Answer: Even though the solutions have identical concentrations, the starting solutions in figures 3c (starting with a solution at 30 ºC) and figures 3d (starting with **Agg D** at room temperature) are different, as well as the temperatures at which the final spectra are taken. Therefore, we were not expecting the same percentages of the different aggregates formed.

(vi) In the time-dependent evolution of CD of Agg B and Agg C, is there an observable lag phase at the initial stage? If so, how does the lag phase depend on concentration?

Answer: We thank the reviewer for addressing this point. The time evolution from **Agg B** and **Agg C** to the other species takes too much time (> 7 days to appreciate any slight change) and we did not measure it directly. What we have done is to follow the evolution of formation of **Agg D** from a monomeric state at different concentrations. This monomeric state was achieved by applying a quick cooling rate (10 K / min) from 70 to 15 ºC, following the kinetics afterwards. As seen in Figure S23, there is a slight lag phase and an abrupt change when **Agg D** is formed. The representation of log t50 vs log C monomer already unravels a lineal trend with a slope higher than one as shown by the Amylofit software (γ = -2.777, not shown). The fitting of these curves with the Amylofit software showed that this behavior is more accurately described by the secondary nucleation model, given the smaller mean residual error obtained in these fittings when compared to the other models provided in the software (see New Figures S22, S23 and S24).

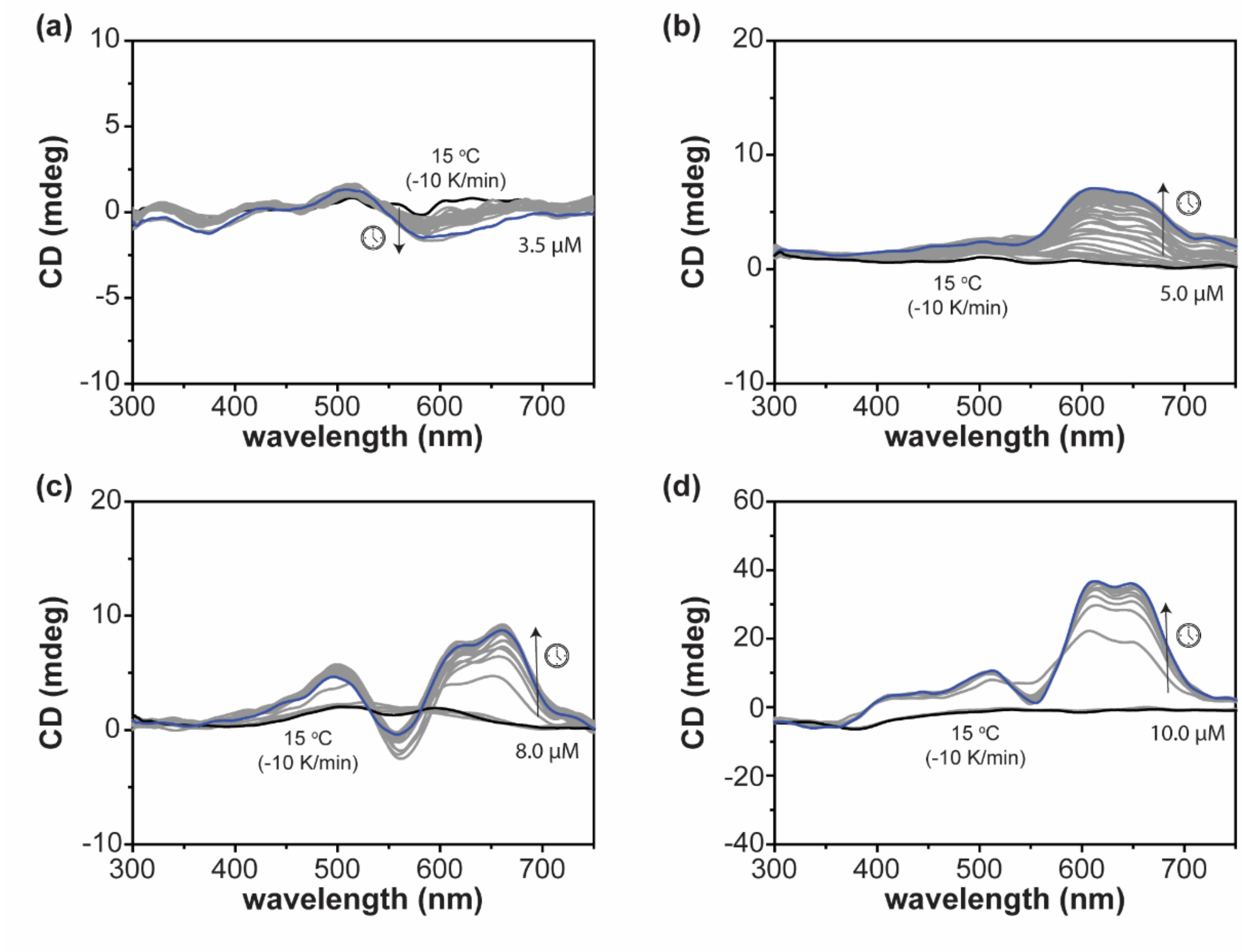

New Figure S22. Time dependent CD spectral changes of **QRD** at 15 °C after cooling down from 45 °C (10 K/min) at different concentrations; a) 3.5 µM, b) 5.0 µM, c) 8.0 µM, and d) 10.0 µM recorded in 10% DCE/MCH.

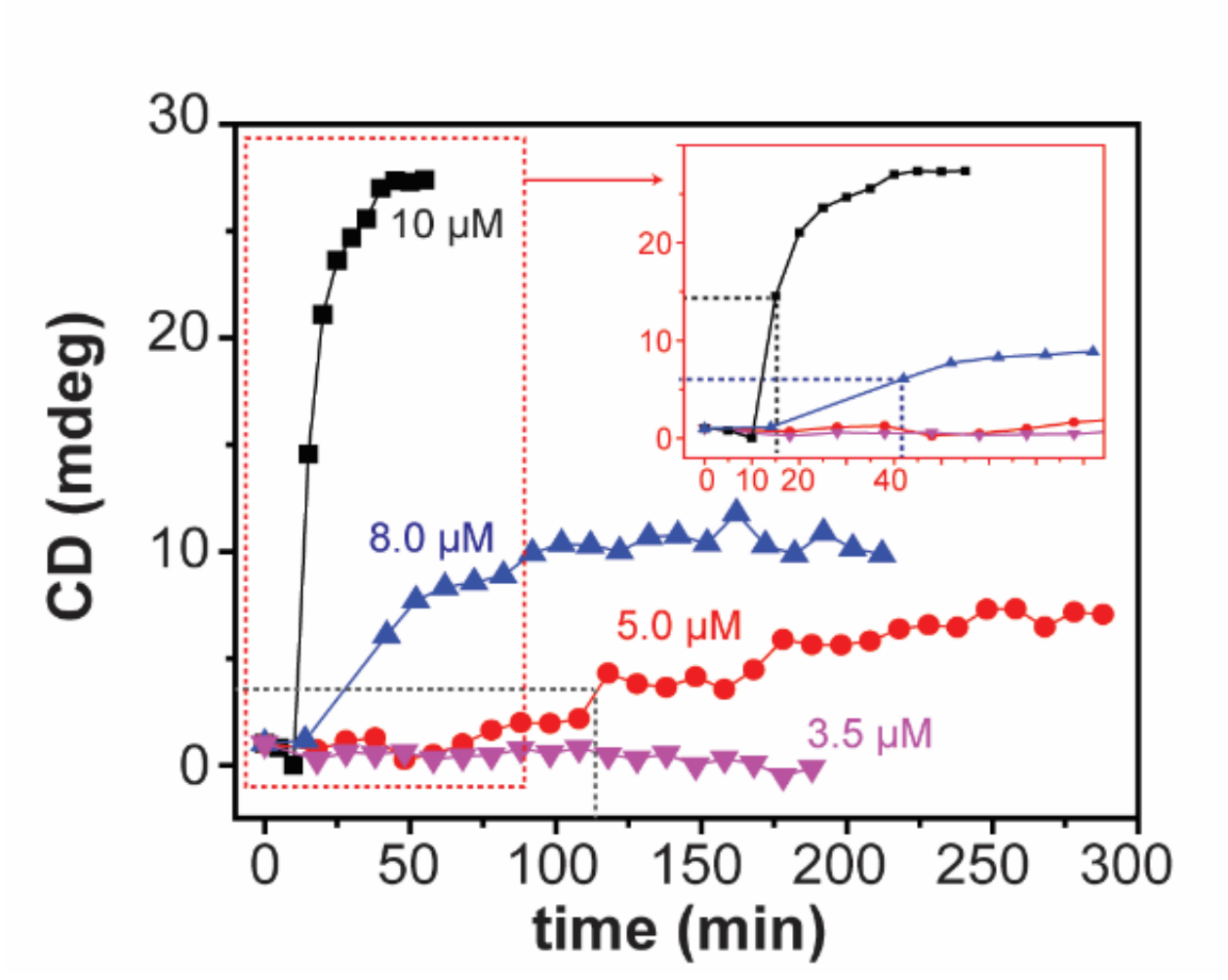

New Figure S23. Time dependent CD intensity changes of QRD monitored at 653 nm upon cooling down to 15 °C at different concentrations.

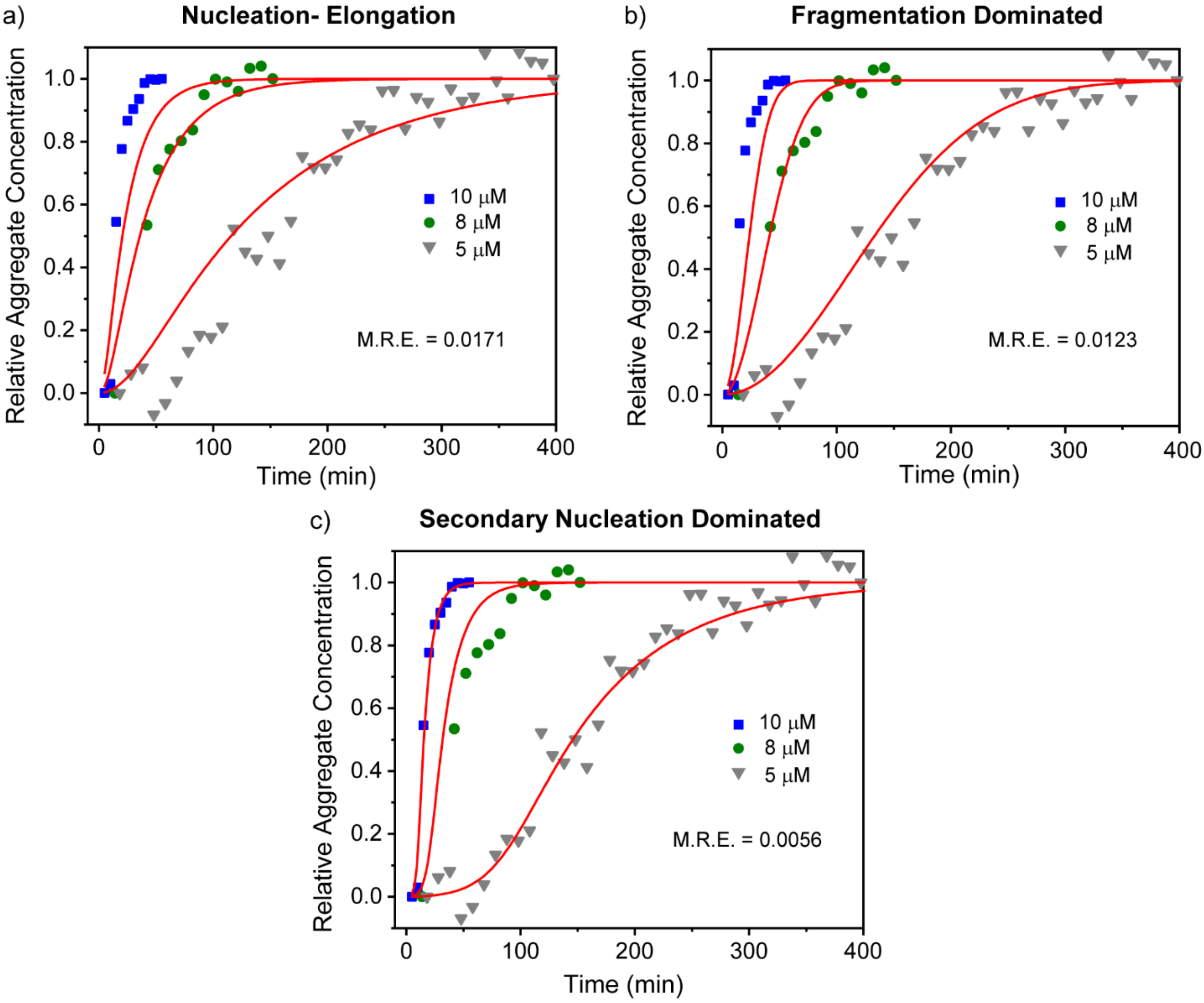

New Figure S24. Kinetics of formation of **Agg D** from the monomer at different concentrations (blue squares 10 μM, green circles 8 mM and grey inverted triangles 5 mM). Fittings of the curves to the (a) nucleation-elongation model (b) Fragmentation dominated model and (c) secondary nucleation model of the Amylofit software.

(vii) The heating-cooling curves of Agg D show a slightly negative value of the CD signal at high temperature instead of zero (Fig. S14). Does the solution contain some residual aggregates?

Answer: We have checked our data and we think it is a small baseline error.

Changes to the main text or SI are highlighted in YELLOW.

Reviewer #1 (Remarks to the Author):

I am fully satisfied with the modifications concerning my technical questions. However, the authors have not replied to my main concern. It is still difficult to see how the specific results described can be of general value and how they would translate in terms of functionality (besides CD signals). I therefore cannot support publication in this journal.

Answer: We thank the reviewer for acknowledging that the technical concerns have been satisfactorily addressed. We also appreciate the reviewer's important comment regarding the broader significance and functional relevance of the system.

Besides CD signals, we have provided an experimental framework to directly determine the energy of assembly of each aggregate without relying on models, and we have performed seeding experiments between aggregates of different orders of hierarchy and elucidated the role of secondary aggregation in the interconversion between aggregates. We strongly believe, these results go further than CD signals and that are of interest for the supramolecular chemistry and chirality communities.

It is well-known that biopolymers such as proteins and DNA undergo transitions from lower-order structures to higher-order ones to achieve more precision, selectivity and efficiency in their respective functions. In our case, it is possible to achieve such level of complexity in artificial systems, with a single molecular scaffold, targeting emergent applications.

While pathway complexity and hierarchical chirality have each been widely studied independently, experimentally resolved systems in which both phenomena can be directly connected through thermodynamic and kinetic relationships remain extremely rare. In this context, our system provides a model platform to understand how assembly pathway selection alone can program higher-order chirality and hierarchical organization without requiring molecular redesign or changes in chemical composition.

Importantly, the broader relevance of such control extends beyond circular dichroism itself. Recent studies have demonstrated that pathway complexity and hierarchical organization can strongly influence emergent functional properties in supramolecular materials, including circularly polarized luminescence and chiral-induced spin selectivity (CISS). For example, secondary nucleation and hierarchical helicity have recently been shown to modulate spin-selective charge transport and chiroptical amplification in supramolecular polymers (*Nat. Nanotechnol*. **2025**, *20*, 825; *Chem. Sci*. **2022**, *13*, 11577). In addition, we previously reported differences in spin selectivity between related aggregates in this system (*J. Chem. Phys*. **2023**, *159*, 114903).

To better emphasize this broader context and the potential functional implications of pathway-controlled hierarchical assembly, we have revised the introduction accordingly. The changes consisting of additional short paragraphs have been highlighted in yellow:

Specifically, we now discuss how pathway complexity and secondary nucleation can influence emergent optical, electronic, and spin-selective properties in chiral supramolecular materials, as in the recent *Nat. Chem.* (2026). https://doi.org/10.1038/s41557-026-02151-4, showing how catalysis depends on pathway control.

Paragraph 1 (page 3): "Importantly, recent studies have shown that such functional responses are not determined solely by molecular structure, but also by the supramolecular pathway through which chiral architectures emerge. In particular, pathway complexity and secondary nucleation processes have been demonstrated to influence spin-selective charge transport and chiroptical amplification in supramolecular polymers.[8,9] These observations suggest that controlling hierarchical self-assembly may

provide access to multiple functional states from a single molecular scaffold, without requiring chemical redesign".

Paragraph 2 (page 4): "Such hierarchical transitions are particularly attractive because the emergence of superhelical organization can strongly modify excitonic coupling, optical activity, and collective electronic behaviour relative to the primary helical state.[32] For example, helix-to-superhelix transformations have recently been associated with enhanced chiroptical responses and tunable circularly polarized emission in π-conjugated supramolecular systems.[33]"

Furthermore, we clarify how the present work establishes mechanistic design principles for accessing structurally and potentially functionally distinct states from a single molecular building block. This change can be found in the closing paragraph of the introduction (page 4): "Beyond establishing a unique example of mechanistically resolved supramolecular polymorphism, this work highlights how pathway selection can be used to program structurally and functionally distinct chiral states from a single molecular building block. More broadly, these results establish design principles for responsive supramolecular materials in which optical, electronic, or spin-selective properties may be tuned through control over the assembly pathway rather than molecular redesign".

We have included the above-mentioned references, which are now references 8 (*Nat. Nanotechnol*. **2025**, *20*, 825), 9 (*Chem. Sci*. **2022**, *13*, 11577), 32 (*Angew. Chem. Int. Ed*. **2019**, *58*, 17994) and 33 (*Chem. Commun*. **2024**, *60*, 6631).

Reviewer #2 (Remarks to the Author):

After the current revision, the originality, novelty, and contribution of this work were presented more clearly in the updated introduction. The authors have demonstrated the incorporations of secondary nucleation into the pathway complexity, in generating distinguishable and isolatable structures supported by detailed CD discussions. The insightful view of the helix formation, especially the newly added experiments regarding pathway of Agg A to Agg B, is fruitful for understanding the energy landscape of the current system. In considering the improved experimental results and discussion, the reviewer suggests addressing some remaining issues before considering acceptance in the in Nat. Commun., as mentioned below.

Answer: We thank the referee for the careful review of our work and for the insightful comments that are helping us a lot to improve the quality of the manuscript.

For XRD measurements which can obtain crucial information for aggregation patterns, the authors did not provide deep insight into the structures. In PXRD experiments, it is suggested that a thinner glass substrate would be used to reduce background signals, and extended exposure time the increase the sample signals with reduced background signal.

Importantly, the authors should provide detailed experiment setups (X-ray source, exposure time, etc.) and sample preparation protocols for clear understanding of the provided data, which may give direct hint on molecular arrangements.

Answer: We agree with the referee that we did not provide deep insight into the structures regarding aggregation patterns due to the poor signals obtained. We have followed the referee´s suggestions and we have optimized the PXRD measurements.
Agg A-D were prepared following the described protocols in section 2 of the supporting information (20 μM, 10% DCE/MCH) and the CD signal was confirmed prior measuring.

The samples were deposited onto a zero-background silicon holder (Silicon low background sample holder for small sample amounts, Ø 51.5mm, Ø 25mm Si cristal, Bruker link). Powder X-ray diffraction (PXRD) patterns were collected over the 2θ range of 5–40° using a step size of 0.016° and a counting time of 1.5 s per step. Measurements were performed on a Bruker D8 Advance powder diffractometer equipped with Cu Kα radiation and a LynxEye position-sensitive (PSD) rapid detector.

Compared to the previous measurements onto glass substrates, the PXRD measurements on the zero-background silicon holder provided different PXRD patterns for all the aggregates (Figure 2.1 for referee). PXRD patterns of **Agg A-D** showed broad peaks due to the amorphous nature of the samples. All of them show a common broad reflection at 12.4° ($d \approx 7.1$ Å), which can tentatively be assigned to short-range correlations involving the amide-containing side chain domains, in agreement with previous reports on amide-functionalized π-conjugated supramolecular systems (Macromolecules 2018, 51, 950-961, figure below).

[REDACTED]

Interestingly, the helical aggregates **Agg B** and **Agg C** display an additional sharp reflection at 31.7° ($d \approx 2.8$ Å), suggesting a higher degree of local molecular order in these structures. These two aggregates have similar morphologies observed by microscopy (confocal super resolution and dry AFM), and their assembly energies are also very close (calculated by ITC). These results point out to very similar structures of opposite handedness. In contrast, the superhelical aggregates **Agg A** and **Agg D** do not exhibit this sharp reflection, but instead show broad diffuse features, consistent with lower long-range positional order and increased structural heterogeneity arising from hierarchical bundling of the helical fibers.

Moreover, **Agg A** and **Agg B** share several common diffraction features, which are rather diffuse, broad and with low intensity in the range of 14° to 25°, supporting the proposed structural relationship between the helical and superhelical states.

It is important to note that the only π-stacking reflection observed at 24.3° ($d \approx 3.7$ Å) was for **Agg B**. It seems that this broad peak is also present in **Agg C** pattern, but it is difficult to distinguish due to the low intensity and broad nature of the signal.

We have added figure b in the SI (new Figure S10) together with a description with details of how PXRD was acquired on page S2. The changes are highlighted in yellow.

For the SI:

Powder X-Ray Diffraction (PXRD)

Agg A-D were prepared following the described protocols in section 2 of the supporting information (20 µM, 10% DCE/MCH) and the CD signal was confirmed prior measuring. The samples were deposited onto a zero-background silicon holder (Silicon low background sample holder for small sample amounts, Ø 51.5mm, Ø 25mm Si cristal, Bruker). Powder X-ray diffraction (PXRD) patterns were collected over the 2θ range of 5–40° using a step size of 0.016° and a counting time of 1.5 s per step. Measurements were performed on a Bruker D8 Advance powder diffractometer equipped with Cu Kα radiation and a LynxEye position-sensitive (PSD) rapid detector.

We have added a short description in the main text and figure b in the SI as new Figure S10. The changes are highlighted in yellow on page 7: "**Agg B** and **C** have a higher local order as evidence by a sharp peak with d-spacing 2.8 Å (Figure S10), whereas **Agg A** and **D** have broad undefined Bragg reflections pointing to a more amorphous packing."

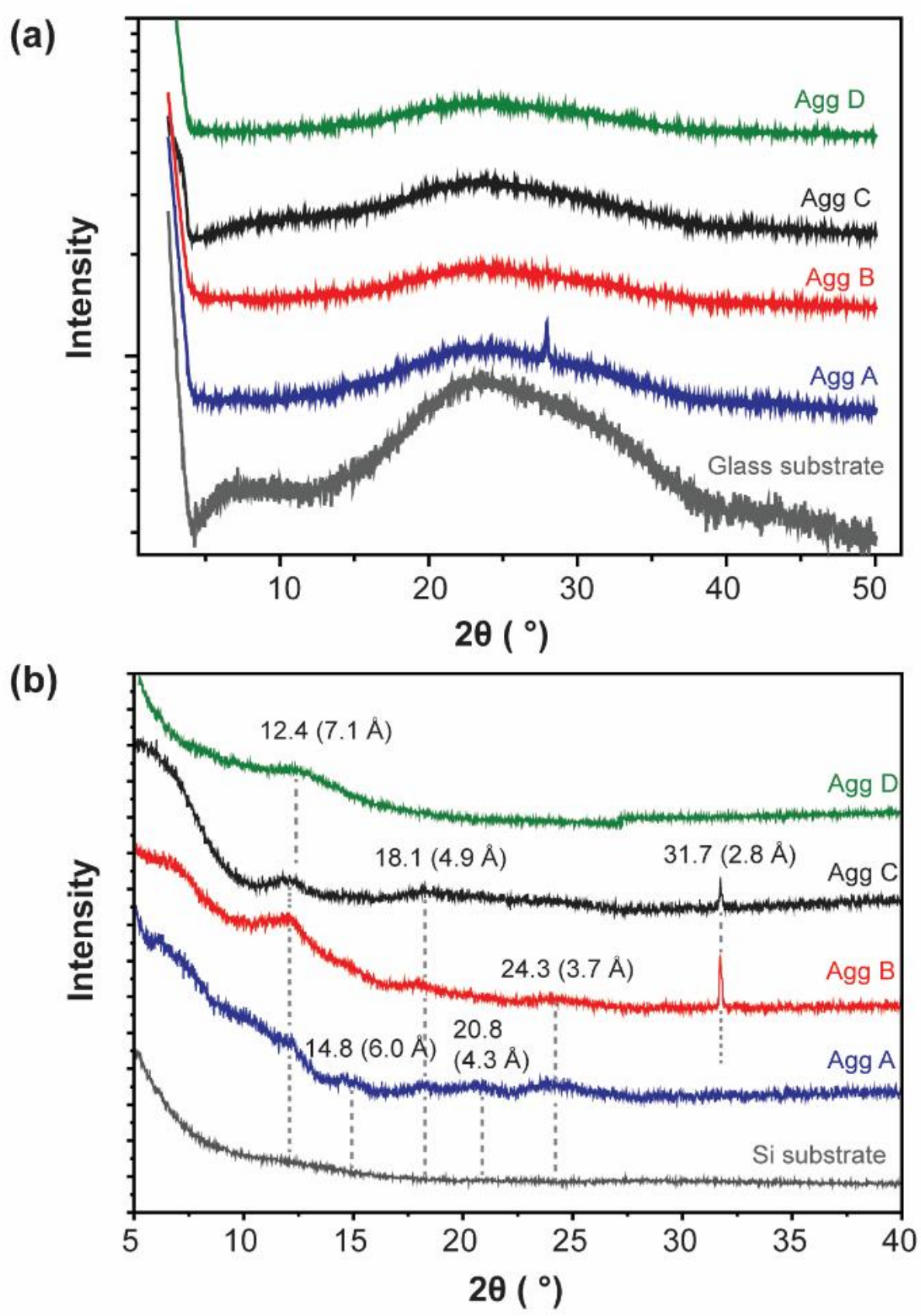

Figure 2.1 for referee. Powder x-ray diffraction patterns measured on different substrates: a) measurements performed on glass substrates, b) new measurements performed on zero background Si-holder.

The SAXS results presented are inadequate. A rapid attenuation of signal even below region of q < 0.1 Å-1 suggests poor structure detected, which is in sharp contrast to the well-defined morphologies reported by microscopies (for reference see Nat. Commun. 2025, 16, 9316). The authors should re-examine the experiment setup and sample preparation method, and provide detailed fitting of their improved data.

Answer: We fully agree with the referee´s comment since the well-defined morphologies are in sharp contrast with the SAXS results. We would like to note, however, that SAXS and microscopy probe different structural aspects of the assemblies. While AFM and confocal microscopy reveal the existence of well-defined helical and superhelical morphologies at the mesoscale, SAXS is sensitive to electron-density correlations and internal structural periodicity in solution. In supramolecular polymers with limited long-range internal order, broad size distributions, and flexible hierarchical organization, well-defined morphologies may coexist with weak or poorly resolved SAXS features.

Following the referee´s suggestion, we addressed this concern, and additional SAXS measurements were performed for **Agg A–C** (**Agg D** could not be measured due to sample limitations) using a Xenocs Xeuss Pro instrument at multiple detector distances to cover a broad q range. The experiments and sample preparation were carried out in the group of Prof. Ronit Bitton (Department of Chemical Engineering, Ben-Gurion University of the Negev, Israel), who has extensive experience in SAXS characterization of supramolecular systems.

The raw scattering profiles showed detectable differences between the aggregate samples and the solvent at low range (Figure 2.2 for referee), confirming the presence of large supramolecular aggregates. However, after solvent subtraction (Figure 2.3 for referee), the resulting SAXS curves mainly provided information associated with aggregate dimensions larger than ~100 nm, while no well-defined oscillations or correlation peaks suitable for reliable fitting or structural modeling could be identified.

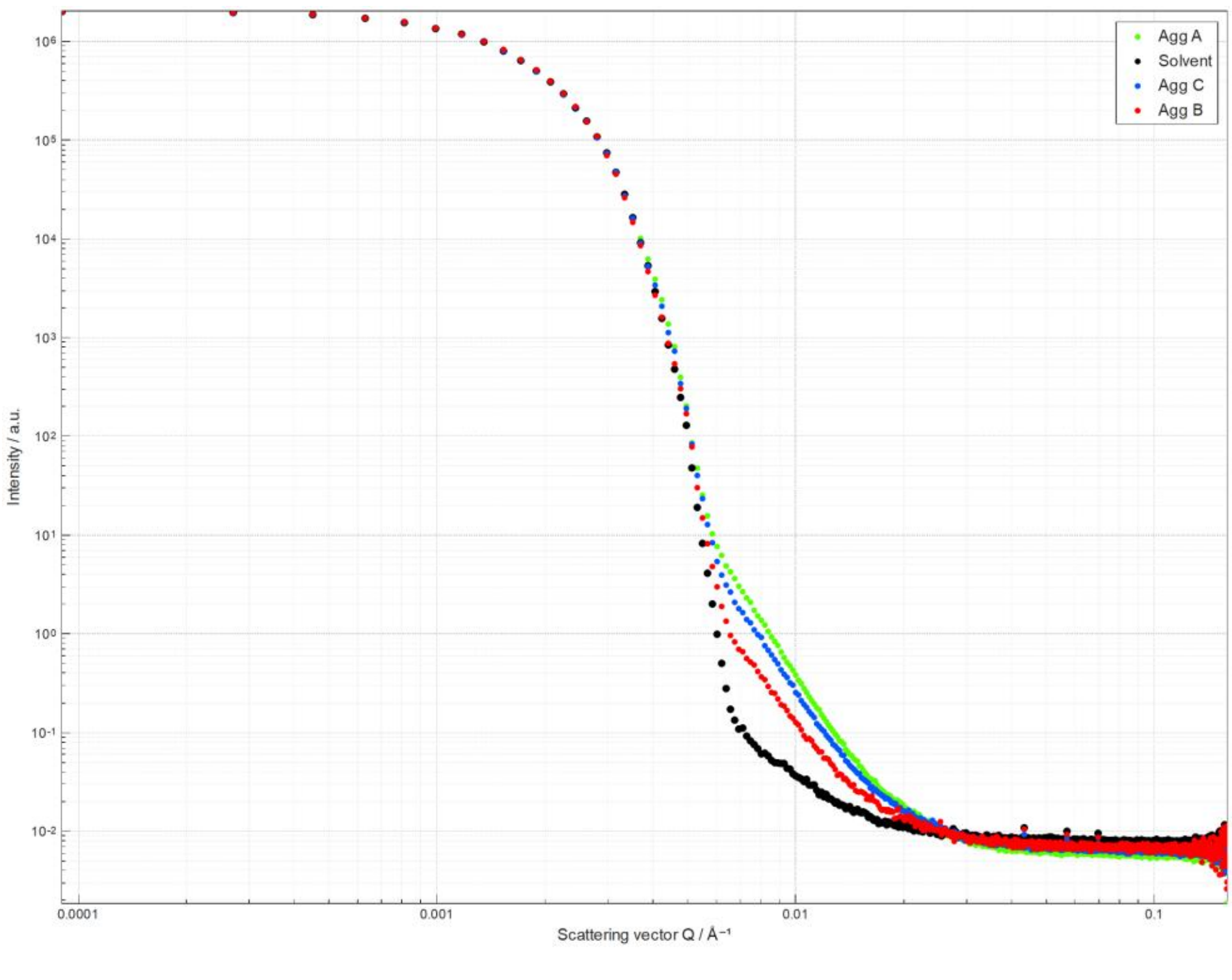

Figure 2.2 for referee. SAXS raw data of **AggA-C** and the solvent (1:9 v/v mixture)

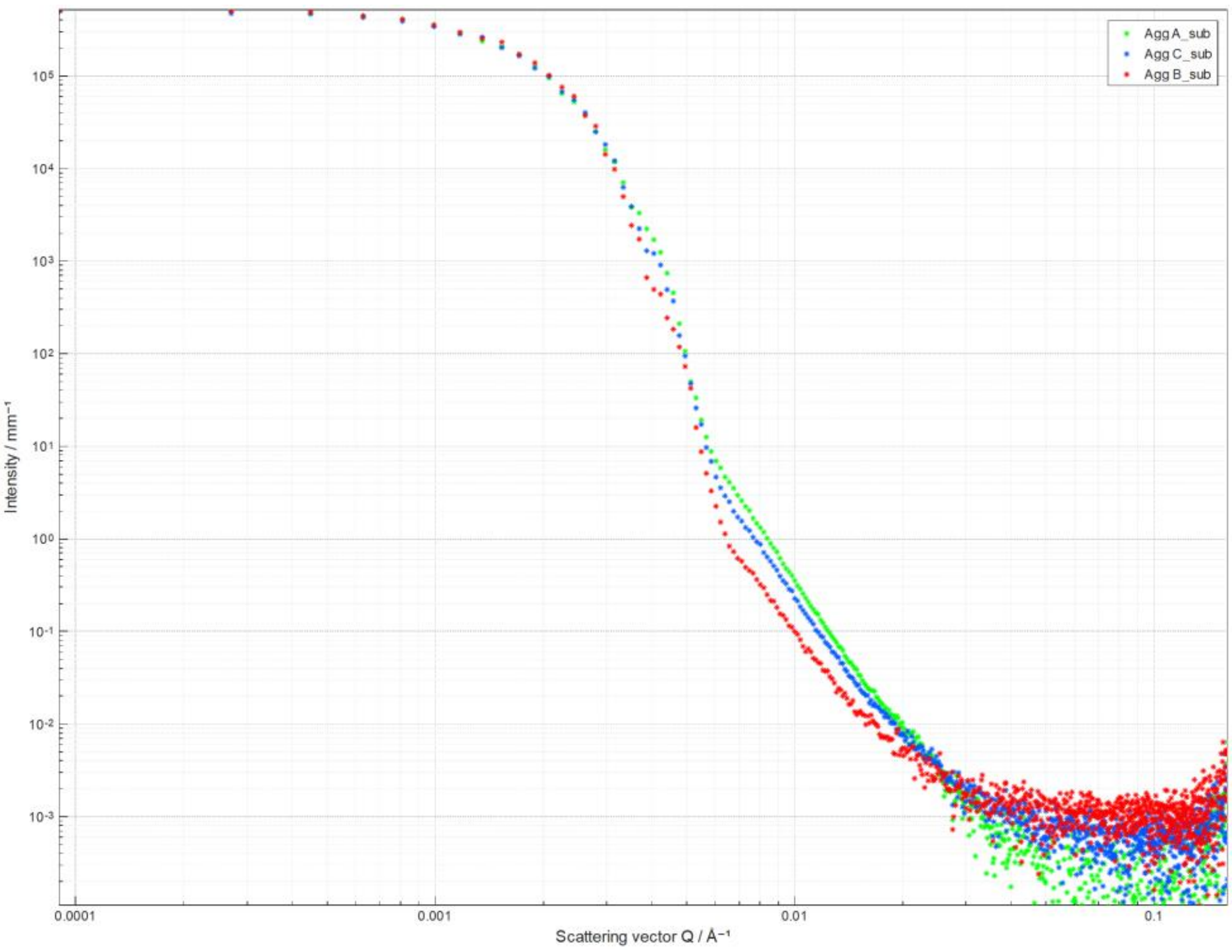

Figure 2.3 for referee. SAXS curves after solvent subtraction

In particular, the characteristic oscillatory features typically observed for ordered nanohelical assemblies, such as those previously reported by the group of Prof. Bitton for related supramolecular systems (*J. Am. Chem. Soc.* **2018**, *140*, 14945−14951, Figure 2.4 for referee) were absent. After repeated measurements and analyses, we concluded that the combination of large aggregate size, the presence of many different structural features, and highly amorphous internal organization prevented extraction of meaningful structural information from SAXS.

[REDACTED]

Figure 2.4 for referee. a) The best fit (solid black line) of the helix model described by Eq.1 (below) to the SAXS curve of K_sEEK_s. Inset is a schematic illustration of the helix model. b) TEM micrograph of K_sEEK_s

[REDACTED]

Equation 1 for referee (From the SI of *J. Am. Chem. Soc.* **2018**, *140*, 14945−14951)

Importantly, these observations are also consistent with the PXRD results obtained for the aggregates. The PXRD patterns do not show sharp Bragg reflections indicative of long-range molecular ordering, but instead display broad and weak diffraction features characteristic of poorly crystalline or predominantly amorphous supramolecular assemblies. Therefore, although microscopy clearly reveals defined mesoscale morphologies, both SAXS and PXRD suggest that the internal molecular packing lacks sufficient long-range periodic order to generate well-resolved structural data.

In FT-IR results presented, why different concentrations of aggregates were used for different solvent mixtures? Also, a 20 μM solution may be too dilute to afford a good signal-to-noise ratio. In summary, detailed structural characterizations can illustrate the intrinsic properties of distinguishable helical structures, and can further resolve the bundling and debundling processes of superhelix observed.

Answer: We added the following FT-IR graphs in the previous round of revisions as “Figure for referee 2.1”. We explained that there were no differences in IR spectra among the different aggregates even after measuring at higher concentration. Nevertheless, we did not explain why we modified the solvent mixture and we apologize for the possible confusion.

Therefore, we would like to address this issue: we performed FT-IR measurements in solution preparing the aggregates according to the protocols described in the SI (section 2). At the concentrations used to perform the spectroscopy studies (10-20 μM), such as CD or UV, in the 1/9 DCE/MCE mixtures, no signals were found and we could not increase the concentration further in this solvent mixture due to poor solubility and precipitation. Therefore, we looked for other conditions where we could increase the concentration to obtain FT-IR signals, but where we could still reproduce the CD signal of each aggregate. We replaced DCE in the mixture by chloroform ($CHCl_3$). In this case, we can reproduce the CD signals of three of the aggregates (Figure 2.5 for referee), while increasing the concentration up to 100 μM. Increasing even further the concentration to 200 μM, allowed us to reproduce the CD signals as well, but we obtained saturated CD signals. Still, the main features of each signal were found and we

proceeded to measure FT-IR at this concentration in a 1/9 $CHCl_3$/MCH mixture. We could not measure **Agg A** under these conditions due to precipitation at this higher concentration.

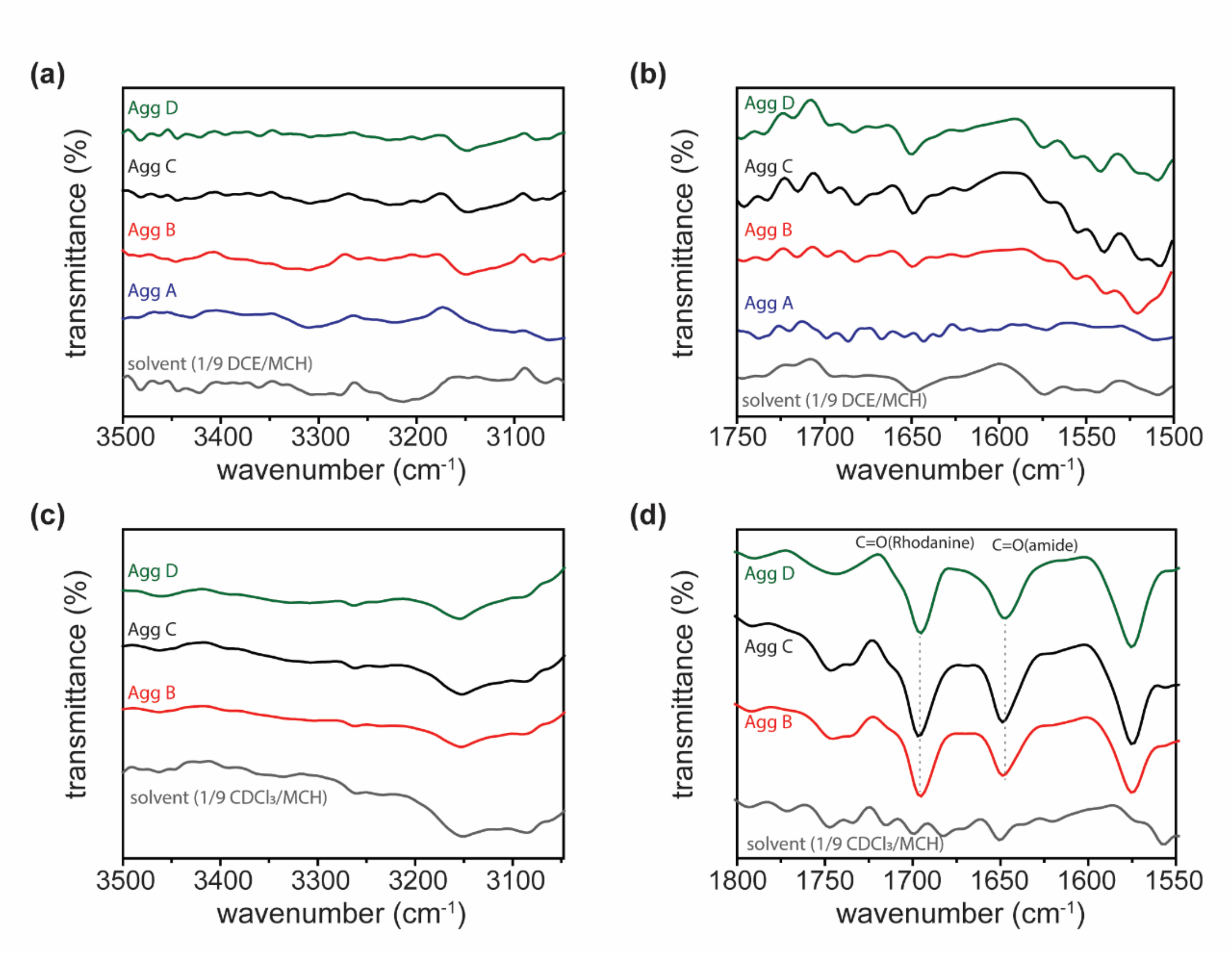

Figure for referee 2.1 (from the previous round of revisions). Partial FT-IR spectra of **Agg A-D** prepared in 1/9 DCE/MCH (20 µM); a) N-H region and b) C=O region, partial FT-IR spectra of **Agg B-D** prepared in 1/9 $CHCl_3$/MCH (200 µM); c) N-H region and d) C=O region.

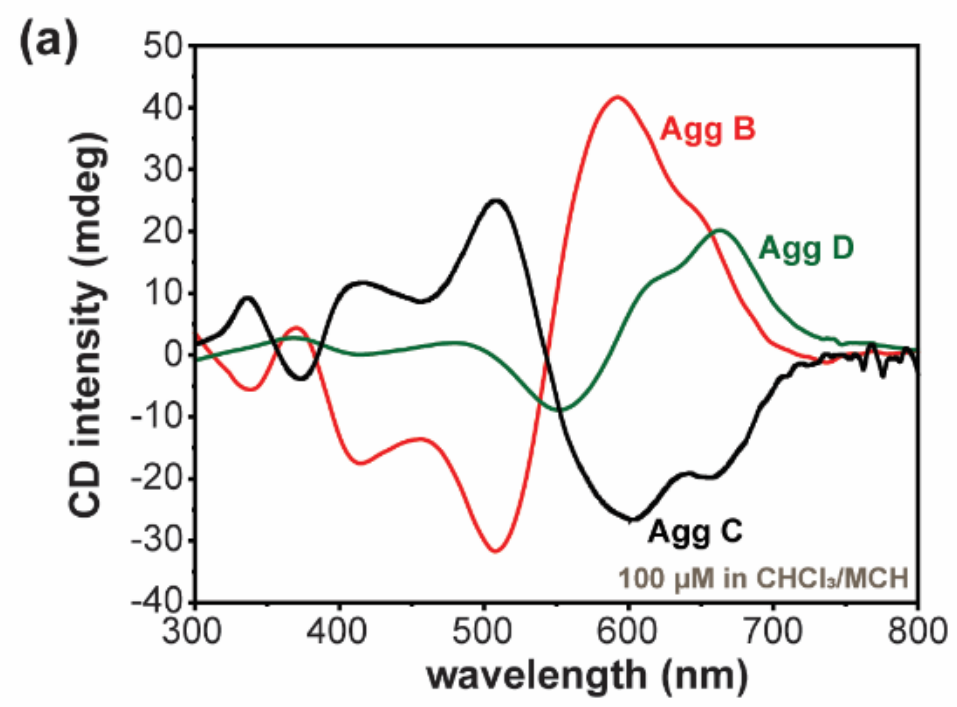

Figure 2.5 for referee. CD spectra of **Agg B, Agg C**, and **Agg D** recorded in 1/9 $CHCl_3$/MCH (100 µM)

Reviewer #3 (Remarks to the Author):

Title: From Helices to Superhelices: Hierarchical Assembly across Competing Pathways
General Assessment: I appreciate the authors' effort in revising the manuscript entitled "From Helices to Superhelices: Hierarchical Assembly across Competing Pathways" in response to my previous comments. The revised version shows noticeable improvement, particularly in the introduction section, which now presents a clearer and more relevant context for the study. As encouraged in my previous report, the authors have undertaken a more detailed investigation into the role of secondary processes in the hierarchical assembly mechanism, and the results suggest that secondary nucleation plays a dominant role in this system. However, several major discrepancies remain in the revised manuscript. The following points should be carefully addressed to strengthen the credibility of the work further.

Specific Comments:

(i) The authors state that the conditions leading to the formation of Agg D are consistent with hierarchical growth processes favoring secondary nucleation and subsequent fiber bundling. However, kinetic analysis is provided only for Agg D, while analogous analyses for the other aggregates are avoided. A comparative scaling analysis is necessary to elucidate the relative contributions of primary and secondary nucleation pathways governing the formation of distinct hierarchical morphologies (helices vs. superhelices i.e., Agg A vs. Agg B and Agg C vs. Agg D). The corresponding scaling plots and a schematic illustration of the secondary nucleation mechanism-mediated hierarchical self-assembly mechanism should be clearly shown in the main manuscript.

Answer: We thank the referee for this valuable input. We also intended to assess the contributions of primary and secondary nucleation for **Agg A-Agg C**. However, there are two issues: i) the Amylofit software evaluates the transition of the monomer to the aggregate state (with or without seeding)—therefore, the available models are not suitable to analyse all of our aggregates; and ii) due to the unique combination of sonication, temperature and cooling rates required to prepare most of the aggregates, we could only monitor the transition to **Agg D** in real time, but not for the other aggregates.

We have included this information in the main text. The changes are highlighted in yellow on page 11: "The Amylofit software evaluates the transition of the monomer to the aggregate state (with or without seeds) and therefore, the available models are not suitable to analyse the rest of the aggregates. Furthermore, due to the unique combination of sonication, temperature and cooling rates required to prepare most of the aggregates, we could only see the transition from the monomer to **Agg D**, making the evaluation of the kinetics of formation of **Agg A-Agg C** with the Amylofit software not accurate".

(ii) The authors rationalize chirality inversion during helix-to-superhelix transitions in terms of stress relaxation of the resulting helical structure (energetic reasons). This interpretation is consistent with the transition from helices Agg C ($\Delta H_{assem, C}$ = –176.7 ± 3.5 kJ/mol) to superhelices Agg D transition (superhelices: $\Delta H_{assem, D}$ = –221.2 ± 13.3 kJ/mol), where a clear enthalpic stabilization is

observed. However, this rationale is not consistently supported across the system. The transition from helices Agg B (ΔHassem, B = –159.1 ± 1.7 kJ/mol) to superhelices Agg A (ΔHassem, A = –139.9 ± 3.2 kJ/mol) is associated with an enthalpic penalty. This inconsistency should be addressed to substantiate the authors' interpretation.

Answer: This is a great point raised by the reviewer. Our rational is that **Agg D** is the lowest in energy considering the molecular chirality of the monomer **QRD**. That is, the helicity of superhelix D is enthalpically favorable considering the chiral center in monomer **QRD**. We experimentally validated this by checking the mirror imaged molecule **QRL**. For the latter, all helicities of the aggregates are opposite as can be seen in Figure 3.1 for referee below. Therefore, a match between molecular chirality and superhelicity of **Agg D** is the most straightforward explanation. We opted to not include this in the study, as the 2 opposite monomers with each 4 chiral aggregates would surely confuse the reader.

We have included an additional paragraph in the main text to comment on the difference in ΔH_{assem} between the superhelices. The changes are highlighted in yellow on page 12: "Nevertheless, the transition from helices of **Agg B** ($\Delta H_{assem,\ B}$ = –159.1 ± 1.7 kJ/mol) to superhelices of Agg A ($\Delta H_{assem,\ A}$ = –139.9 ± 3.2 kJ/mol) is associated with an enthalpic penalty. We rationalize this result considering the molecular chirality of the monomer **QRD**. The helicity of **Agg D** is enthalpically favorable considering the chiral center in monomer **QRD**. We experimentally validated it by checking the mirror imaged derivative **QRL** (Figure S27). For the latter, all the helicities of the aggregates are opposite to those shown in Figure 3.1. Therefore, a match between molecular chirality and superhelicity of **Agg D** is the most plausible explanation."

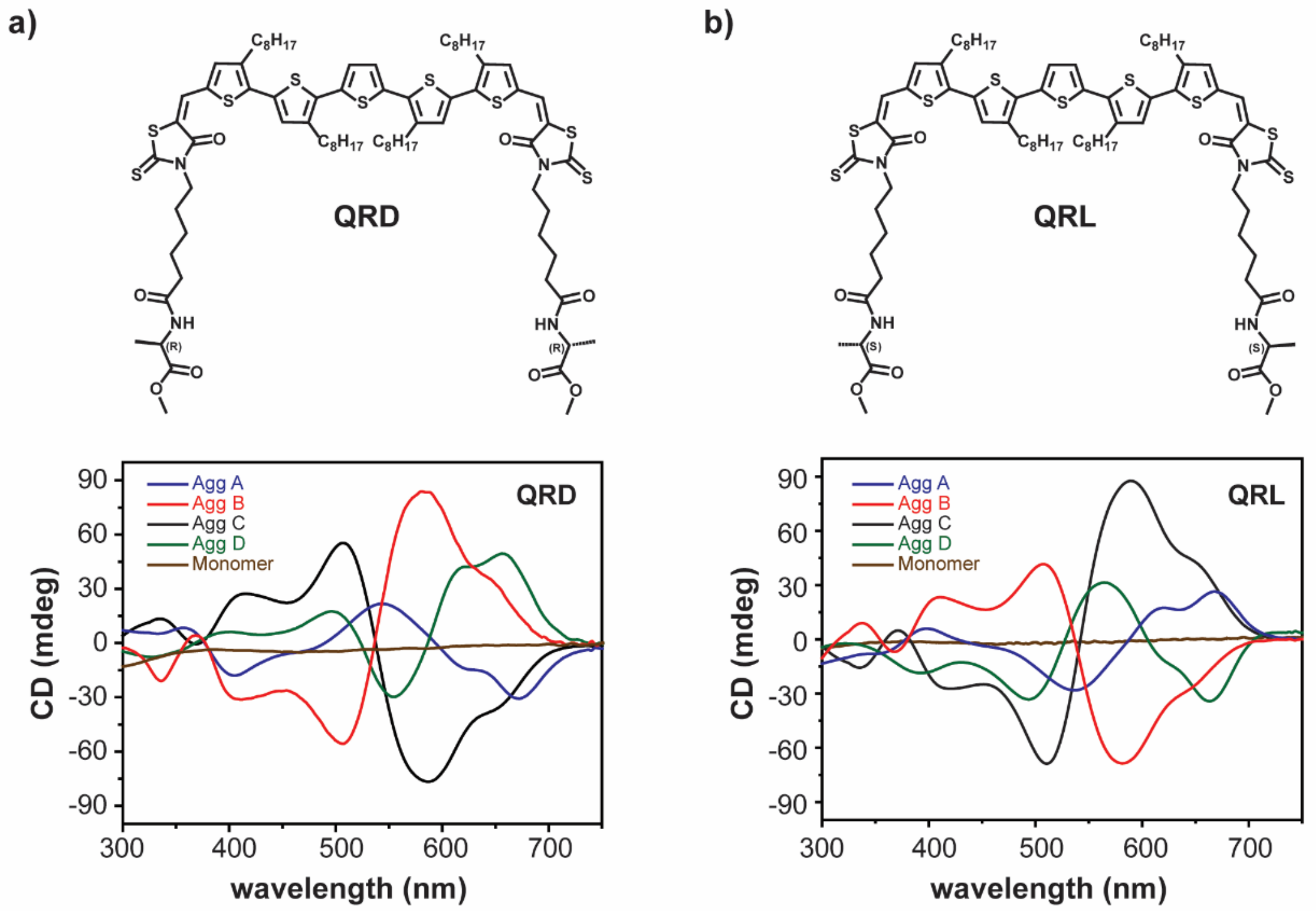

Figure 3.2 for reviewer. CD signals of **QRD** and **QRL** prepared under the same conditions.

Additionally, we have included the CD spectra of **QRL** in the SI as the new Figure S27.

(iii) The authors claim that the transition from Agg B to Agg C is an off-pathway process occurring via the monomer, a conclusion supported by the observed enhancement of the transition rate at lower concentrations. However, the energy landscape presented in Figure 1d shows a direct connectivity between these two states, implying an on-pathway relationship. This inconsistency between the mechanistic interpretation and its graphical representation is problematic. If the interconversion indeed occurs via monomer, the energy landscape should explicitly reflect this by depicting a pathway that traverses the monomeric state rather than a direct connection between Agg B and Agg C. The authors should revise Figure 1d accordingly to ensure consistency with their proposed mechanism.

Answer: We apologize for this mistake and we agree with the referee. This was in fact a point of discussion among the co-authors during the prior submission. Figure 1d has been corrected as stated by the reviewer (see also below), placing the monomer between **Agg B** and **Agg C**, which is indeed the representation which more accurately describes our system.

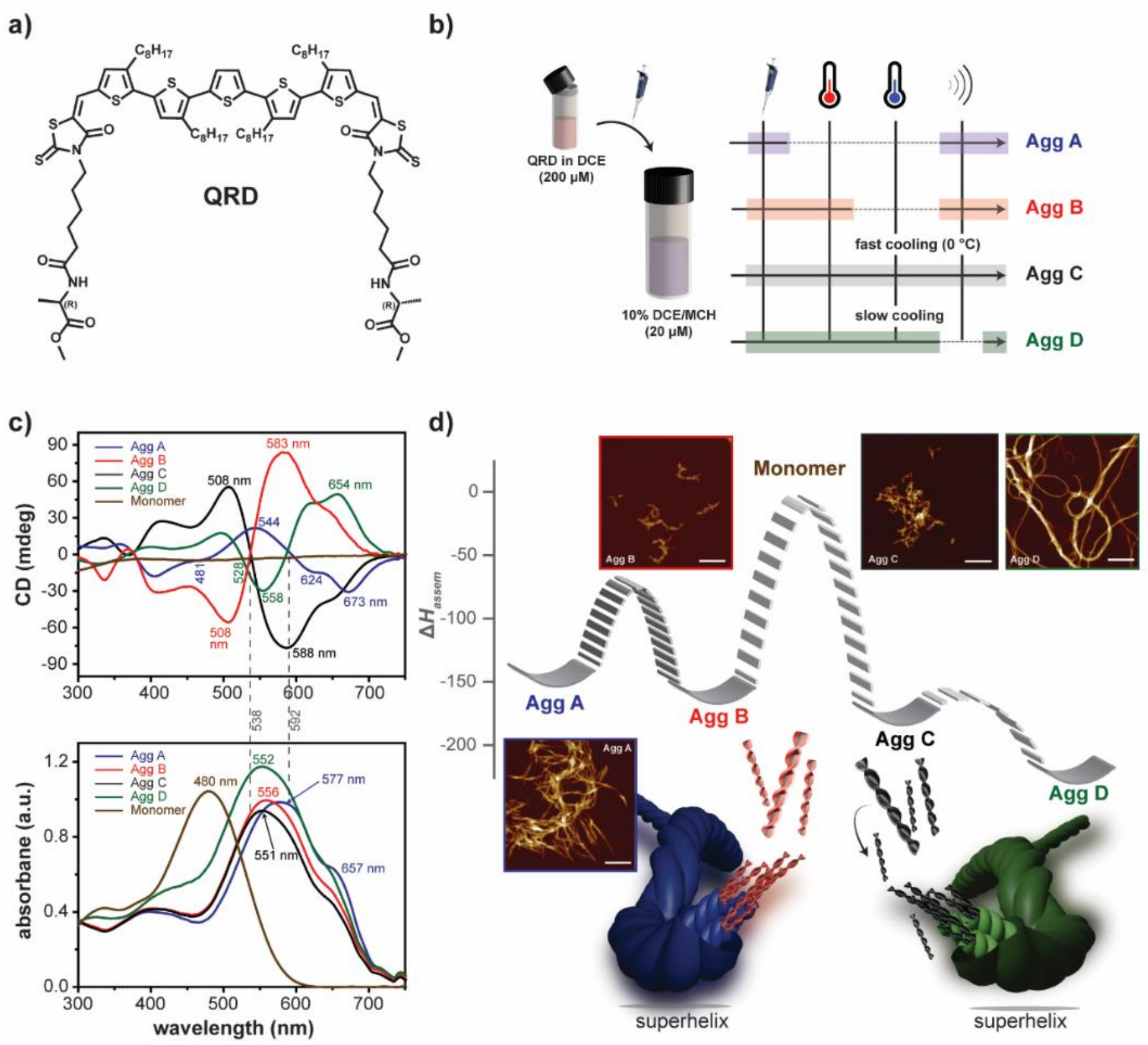

(iv) The manuscript highlights the "long-lived nature" of the isolated aggregates as a key feature that enables direct calorimetric determination of their assembly enthalpies and the construction of a quantitative enthalpic energy landscape. While this represents an impressive experimental achievement, the authors do not provide any discussion regarding the molecular origin of this behavior. It remains unclear what specific features of the molecular design or self-assembly endow such kinetic stability, allowing multiple distinct states to persist under these conditions. The authors should therefore provide a rationale or at least a plausible hypothesis addressing the origin of this long-lived behavior. Such a discussion

would significantly enhance the broader impact of the work and offer valuable insights for the future design of systems with similarly well-defined and long-lived metastable states.

Answer: We thank the referee for this insightful comment. We totally agree this point would enhance the impact of the work, which is one of the main current research topics in our team. We are expecting results from pending funding calls on this matter.

While at this moment we do not have a proven explanation of the molecular origin of this behavior, we have parallel results in analogue derivatives based on different π-conjugated systems (as diketopyrrolopyrrole: https://doi.org/10.1021/acs.jpcc.2c03105 for the structures) and in other QR molecules with different pending chiral units (*Chem. Commun.*, 2022, **58**, 529-532 from our team). In the latter, we did not explore the energy landscape or chirality inversion mechanism, but we reported what at the time was surprising to us, which was the chirality inversion upon changing temperature and solvents. In that case, the two different chiral structures identified were not mirror images and they were isolatable as well.

Interestingly, all the derivatives above-mentioned have in common that the amide unit is separated from the conjugated segment by a flexible linker (4-6 methylene units).

Regarding the molecular design, the A-D-A system π-conjugated segment (shared in DPP and QR derivatives in particular) strongly promotes the formation of J-aggregates in order to maximize the donor-acceptor interactions (see e.g. *Organic Materials* **2020**, 2, 47–63). In addition, the amide functionality enhances the cooperative character of the assembly process, favoring the formation of helical supramolecular structures. Despite the fact that this effect has been barely studied, we hypothesize that locating the chiral center relatively far from the π-conjugated core likely reduces the efficiency of the transfer of asymmetry, making the two primary helical conformations relatively close in energy. Combined with the presence of a relatively long and flexible alkyl spacer (this is also the case of achiral derivatives synthesized by us, such as the DPP example previously mentioned) this may facilitate the formation of kinetically trapped structures, as previously investigated (see e.g., *Chem. Mater.* **2014**, 26, 1, 576–586 or more recent *Chem. Soc. Rev.*, **2025**,54, 11659-11698).

In conclusion, the A-D-A design in the conjugated core, the competition between strong directional H-bonding and π-stacking, together with the presence of flexible linkers favours the formation of aggregates with different competing structures that become long-lived metastable species. We intend to investigate in-depth distinct molecular designs in order to further correlate which molecular requirements are needed to control the formation of isolatable, well-defined and long-lived species.

Following the referee´s advice, we have included a short discussion paragraph in the main text to provide a plausible hypothesis addressing the molecular origin of this long-lived behaviour. The changes have been highlighted in yellow on the main text on page 14 in the conclusions section: "In this sense, there are several design features potentially responsible for the molecular origin of such interesting behaviour. The A-D-A (acceptor-donor-acceptor) of the rhodanine-quinquethiophene conjugated segment promotes the formation of J-type aggregates to maximize the D-A interactions.[62] In addition, the amide functionalities enhance the cooperative character of the assembly process, favouring the formation of helical supramolecular structures. Furthermore, we hypothesize that locating the chiral center relatively far from the π-conjugated core likely reduces the efficiency of the transfer of asymmetry, making the two primary helical conformations relatively close in energy. Combined with the presence of a relatively long and flexible alkyl spacer that promotes the competition between highly directional H-bonding and π-

stacking, resulting in the formation of competing structures that lead to long-lived, metastable species.[63]"

Two additional references, new references 62 and 63 have been included.

Minor comment: γ is the scaling exponent. It should be mentioned with the scaling equation.

Answer: we thank the reviewer for indicating the scaling exponent. We have mentioned it with the scaling equation. The change is highlighted in yellow on the main text on page 10: "The slope, corresponding to the scaling factor γ, obtained after representing log t50 versus log monomer was of γ = -2.777 (not shown)."

Changes to the main text are highlighted in YELLOW. The SI is unchanged.

Reviewer #2 (Remarks to the Author):

The reviewer is satisfied with the efforts of authors concerning xrd-related results. Broadened peaks can be distinguished from PXRD experiments, and SAXS data with absence of oscillation features may indicate the poor long-range order within the assembled structures. It is still obscure to the reviewer how an amorphous system can adopt precise pathway selection in generating isolable and long-live species, with limited structural identifications only accessing the morphologies.

Answer: We thank the reviewer for the constructive comments and for recognizing our efforts regarding the PXRD and SAXS characterization. We appreciate the reviewer's concern regarding how pathway selection can emerge in amorphous systems. We would like to clarify that by "amorphous" we refer to the absence of detectable long-range crystalline order rather than the absence of structural organization. Although atomic-level structural assignment is beyond the scope of the current work, multiple orthogonal observations support the existence of distinct assembled states: (i) each aggregate displays reproducible and distinct CD signatures, (ii) different assembly enthalpies were experimentally determined, (iii) aggregate morphologies were characterized both on surfaces and in solution using super-resolution confocal methods, and (iv) the interconversion behavior depends systematically on the preparation protocols. These observations support the presence of well-defined structures and pathway selection despite the absence of crystallinity. Similar behavior has been reported in pathway-complex supramolecular polymer systems, where kinetically trapped and metastable states emerge without long-range order.

Even the authors claimed that secondary nucleation is a dominating process, the correlation of sample preparation procedures and the assembly pathway selection should be discussed, considering general impact for orchestrating complex but controllable systems.

Answer: We have followed the reviewer´s suggestion on including a discussion on the correlation of sample preparation procedures and the assembly pathway selection to orchestrate complex and controllable systems. In this sense, in the previous version of the manuscript we had described in detail each preparation protocol in the introduction section, we have on purpose not given a correlation with the assembly pathway in this part of the manuscript in order not to give the results away at such an early point. Instead, we included brief discussion paragraphs at different parts of the results section when we described the mechanistic details and interconversions. See for instance:

- End of page 10 and beginning of page 11: "Thus, we first attempted to accelerate the conversion of **Agg C** into **Agg D** with different (**Agg D**) cross-seed amounts, but no rate change was observed (Figure S22). At this moment, we suspected that in **Agg D** formation there was a large contribution of secondary nucleation due to the protocol applied (slow cooling rates and plausible supersaturation conditions)."
- Last paragraph of page 12: "The conditions that produce **Agg D** (i.e., supersaturation combined with slow cooling) consistent with hierarchical growth processes that favour secondary nucleation and subsequent fiber bundling.

Similarly, the formation of **Agg A** under rapid solvophobic quenching conditions suggests that this superstructure may originate from secondary nucleation processes involving **Agg B** and **Agg C**."

Nevertheless, we agree with the reviewer that the discussion should be clearer. Therefore, we have included a conclusion paragraph at the end of the results section, on page 13, emphasizing how each preparation protocol leads to the assembly pathway selection.
The changes are marked in yellow in the revised manuscript:

- Page 13: "More broadly, the preparation protocols themselves act as pathway-selection variables. Cooling rate, thermal treatment, and mechanical perturbation modify local supersaturation and nucleation kinetics, thereby leading to competing assembly routes. Under slow cooling, gradual equilibration favors hierarchical growth and secondary nucleation processes that ultimately yield **Agg D**. In contrast, rapid sonication-assisted protocols combined with thermal treatment (**Agg A-C**) likely generate alternative nucleation environments and debundling that stabilize **Agg B–C** before hierarchical reorganization occurs, emphasizing the crucial role of the preparation protocols in achieving complex but controllable systems.

Reviewer #3 (Remarks to the Author):

I suggested the evaluation of the relative contributions of primary and secondary nucleation processes in the formation of the different aggregates (Agg A–D). The authors have clarified that experimental constraints associated with the preparation protocols prevented kinetic analyses of Agg A–C. Therefore, I do not wish to comment further on this point.

However, my original comment also included a schematic illustration of the proposed secondary nucleation-mediated hierarchical assembly pathway leading to the double-helical Agg D structure. There should be no limitation preventing the inclusion of such a schematic, for example, below Figure 1D. This would help readers visualize the proposed assembly mechanism and strengthen the discussion of Agg D formation.

Answer: We thank the reviewer for the constructive comments and for acknowledging our clarification regarding the limitations associated with evaluating the relative contributions of primary and secondary nucleation in Agg A–C formation.

We apologize for not implementing the schematic illustration of the proposed secondary nucleation-mediated hierarchical assembly pathway mechanism leading to Agg D. As the reviewer points out, there is no limitation preventing the inclusion of such scheme. Therefore, we have followed the reviewer´s recommendation and we have incorporated the illustration about the proposed secondary nucleation assembly pathway as an inset within Figure 1d (see below). We have modified the figure caption accordingly.

The illustration shows how the monomer (represented as brown rods) forms either **Agg B** or **Agg C**, which are close in energy, depending on the preparation protocol. Then, **Agg B** serves as a monomer reservoir, where the monomers assemble through secondary nucleation onto **AggC** to form superhelix **AggD**.

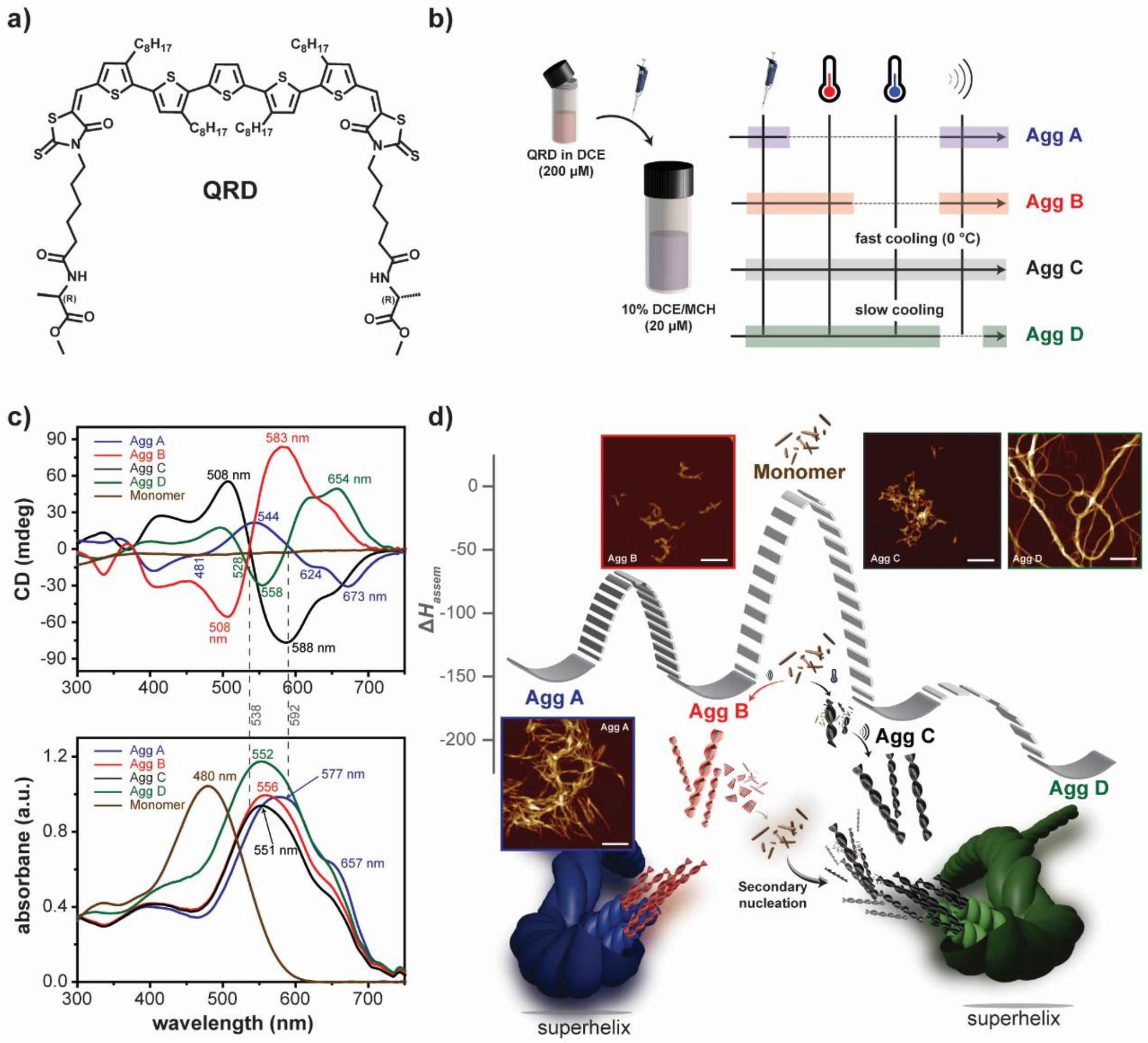

Figure 1. Overview of the QRD system with 4 chiroptical states. a) Molecular structure of the building-block **QRD**. b) Preparation protocols of **Agg A**, **B**, **C** and **D**. c) Top: CD spectra of **Agg A–D** in solution. [**QRD**] = 20 µM 1:9 DCE/MCH (l = 1 cm). Bottom: corresponding UV-Vis spectra at the same conditions. d) Energy landscape with measured ΔH_{assem} of each aggregate determined by ITC. AFM images of **Agg A–D** on mica substrates (scale bars are 1 µm). The cartoons labeled "secondary nucleation" emphasize the hierarchical assembly pathway leading to **Agg D** (superhelix).

We have as well included references to figure 1d mechanistic illustration inset in different parts of the manuscript (all the changes are marked in yellow in the revised version of the manuscript), as for example:

- Page 8: "Thus, these measurements indicate that **Agg D** is much more stable than **Agg A**, whereas **Agg C** is slightly more stable than **Agg B**, with differences in energy that disclose potential transitions between these aggregates (Figure 1d, cartoons), as well as the presence of competing pathways."
- Page 10: "So far, these spontaneous conversions suggest that **Agg B** is *off-pathway* to **Agg C** (this transition occurs *via* the monomer, Figure 1d, cartoons) given the slow transition between these aggregates and the fact that this conversion is slightly enhanced at lower concentrations."
- Page 11: "Taken together, the (un-)seeded experiments suggest the following: *i*) **Agg D** is a superhelix of **Agg C** (Figure 1d, cartoons)."

- Page 11: "*ii*) therefore, **Agg B**—unable to convert to **Agg D**—acts as monomer reservoir accelerating **Agg C** conversion into superhelix **Agg D** (Figure 1d, cartoons and Figure 2c,d); *iii*) this explains why the conversion from **Agg B** to **Agg D** is very fast in presence of seeds"
- Page 12: "Considering the distinct level of organization in the self-assembly of **Agg C** and **Agg D** and the seeding experiments results, it is reasonable to propose that **Agg D** is a superhelical structure formed through secondary nucleation of **Agg C** (Figure 1d, cartoons)".
